# The Regulation of *para*-Nitrophenol Degradation in *Pseudomonas putida* DLL-E4

**DOI:** 10.1371/journal.pone.0155485

**Published:** 2016-05-18

**Authors:** Qiongzhen Chen, Hui Tu, Xue Luo, Biying Zhang, Fei Huang, Zhoukun Li, Jue Wang, Wenjing Shen, Jiale Wu, Zhongli Cui

**Affiliations:** 1 Key Laboratory of Agricultural Environmental Microbiology, the Ministry of Agriculture, Nanjing Agricultural University, Nanjing, People’s Republic of China; 2 State Key Laboratory of Biosafety, Nanjing Institute of Environmental Sciences (NIES), Ministry of Environmental Protection of China, Nanjing, Jiangsu, People’s Republic of China; 3 School of Life Sciences and Biotechnology, Shanghai Jiao Tong University, Shanghai, People’s Republic of China; MJP Rohilkhand University, INDIA

## Abstract

*Pseudomonas putida* DLL-E4 can efficiently degrade *para*-nitrophenol and its intermediate metabolite hydroquinone. The regulation of *para*-nitrophenol degradation was studied, and PNP induced a global change in the transcriptome of *P*. *putida* DLL-E4. When grown on PNP, the wild-type strain exhibited significant downregulation of 2912 genes and upregulation of 845 genes, whereas 2927 genes were downregulated and 891 genes upregulated in a *pnpR*-deleted strain. Genes related to two non-coding RNAs (*ins1* and *ins2*), *para*-nitrophenol metabolism, the tricarboxylic acid cycle, the outer membrane porin OprB, glucose dehydrogenase Gcd, and carbon catabolite repression were significantly upregulated when cells were grown on *para*-nitrophenol plus glucose. *pnpA*, *pnpR*, *pnpC1C2DECX1X2*, and *pnpR1* are key genes in *para*-nitrophenol degradation, whereas *pnpAb* and *pnpC1bC2bDbEbCbX1bX2b* have lost the ability to degrade *para*-nitrophenol. Multiple components including transcriptional regulators and other unknown factors regulate *para*-nitrophenol degradation, and the transcriptional regulation of *para*-nitrophenol degradation is complex. Glucose utilization was enhanced at early stages of *para*-nitrophenol supplementation. However, it was inhibited after the total consumption of *para*-nitrophenol. The addition of glucose led to a significant enhancement in *para*-nitrophenol degradation and up-regulation in the expression of genes involved in *para*-nitrophenol degradation and carbon catabolite repression (CCR). It seemed that *para*-nitrophenol degradation can be regulated by CCR, and relief of CCR might contribute to enhanced *para*-nitrophenol degradation. In brief, the regulation of *para*-nitrophenol degradation seems to be controlled by multiple factors and requires further study.

## Introduction

*para*-Nitrophenol (PNP) is listed as a priority environmental pollutant by the United States Environmental Protection Agency [1]. PNP is a toxic and bio-refractory molecule that is released into the environment via industrial waste and agricultural application of chemical pesticides. PNP is hazardous to humans and a number of animal models [1–3]. Various microorganisms have been reported to degrade PNP, including *Arthrobacter*, *Rhodococcus*, *Bacillus*, *Burkholderia*, and *Pseudomonas* [4–10]. PNP degradation in both Gram-positive and -negative bacteria has been extensively characterized [11–14]. The microbial degradation of PNP has been intensively investigated and two major degradation pathways have been described: the hydroquinone (HQ) pathway and the hydroxyquinol (BT) pathway. Although the effects of some aromatic compounds on mRNA expression profiles have been studied in some organisms [15–18], the effects of PNP on the transcriptome profiles of *P*. *putida* remain unknown.

**Fig 1:**
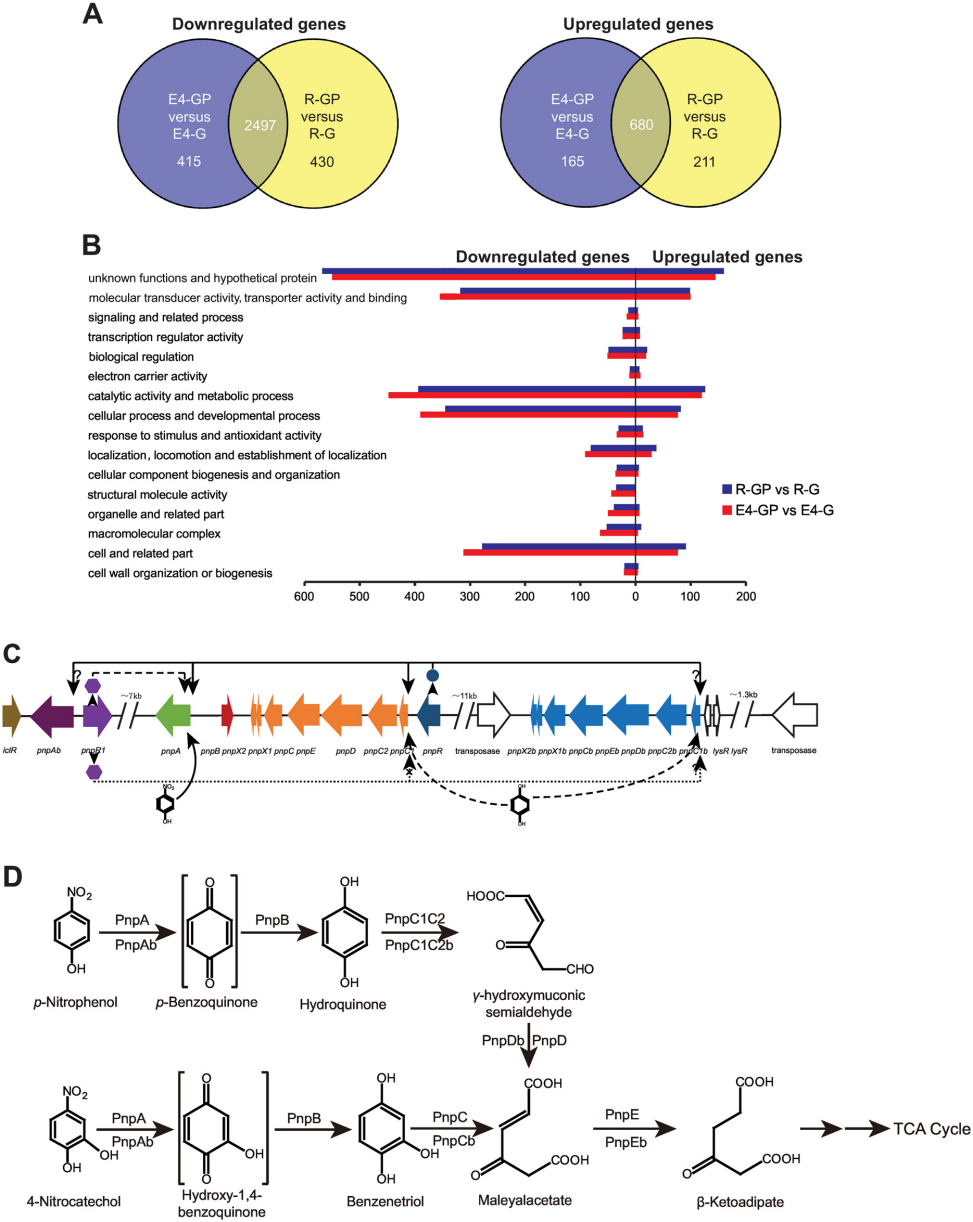
(A) Number of differentially expressed genes under different conditions. Genes downregulated or upregulated in *P*. *putida* DLL-E4 and DLL-Δ*pnpR* grown on glucose plus PNP compared to glucose. Venn diagrams show the overlap between downregulated genes and between upregulated genes for each pairwise comparison. (B) Functional classification of differentially expressed genes under different conditions. Each plot indicates the type of physiological role and the total number of genes with increased or decreased expression within that category in cells grown on the relevant conditions (also see S3 and S4 Tables in the Supporting Information). R-GP refers to *P*. *putida* DLL-Δ*pnpR* grown on 0.25% glucose plus 0.5 mM PNP, R-G refers to strain DLL-Δ*pnpR* grown on 0.25% glucose, E4-GP refers to *P*. *putida* DLL-E4 grown on 0.25% glucose plus 0.5 mM PNP, and E4-G refers to strain DLL-E4 grown on 0.25% glucose. (C) Organization and proposed regulatory circuit of the PNP degradation genes of *P*. *putida* DLL-E4 (figure not drawn to scale). The large open arrows indicate the approximate size of each gene and its direction of transcription. Genes within the same operon are indicated by the same color. Two forward slashes stand for a gap in the genome. The circle and hexagons stand for the products of *pnpR* and *pnpR1*, respectively. The thick arrow stands for the transcription of *pnpR* and *pnpR1*. The thin solid arrow stands for PnpR’s positive action on the two operons *pnpA* and *pnpC1C2DECX1X2*. The thin solid question-marked arrow stands for the unclear regulation of operons *pnpAb* and *pnpC1bC2bDbEbCbX1bX2b* by PnpR. The folded arrow stands for PnpR1’s positive action on *pnpA* operon. The dotted question-marked arrow stands for the unambiguous regulation of the *pnpC1bC2bDbEbCbX1bX2b* operon by PnpR1. The dotted x-marked arrow indicates that PnpR1 does not regulate the *pnpC1C2DECX1X2* operon. The curved solid arrow stands for PNP’s positive action on *pnpA*. The curved folded arrow stands for HQ’s positive action on operons *pnpC1C2DECX1X2* and *pnpC1bC2bDbEbCbX1bX2b*. (D) Proposed pathway for PNP catabolism in *P*. *putida* DLL-E4 with the catabolic reactions catalyzed by PNP degradation gene products.

The regulation of genes involved in PNP degradation has been investigated in *Pseudomonas* [19]. The putative regulator-encoding gene *pnpR* has been identified as involved in PNP degradation [9,19], and the LysR-type transcriptional regulator (LTTR) activates the expression of genes in response to the specific inducer PNP [19]. *Pseudomonas putida* DLL-E4 efficiently degrades PNP and its intermediate metabolite hydroquinone (HQ) [9,20]. Two PNP catabolic gene clusters, *pnp* (*pnpRC1C2DECX1X2BA*) and *pnp1* (*pnpC1bC2bDbEbCbX1bX2b* and *pnpAb*) (Fig 1C and 1D), have been identified in the genome of *Pseudomonas putida* DLL-E4; however, the function of these clusters in the degradation of PNP is unknown.

In *Pseudomonas*, carbon source metabolism is regulated by a complex gene regulatory network at different levels; this network is important in aromatic compound degradation and substrate utilization [21,22]. Carbon catabolite repression (CCR) in the regulation of carbon source utilization is controlled by a global regulator, the Crc protein, in *Pseudomonas* [22]. Although aromatic compounds can be utilized as substrates, they also caused serious stress during the growth of *Pseudomonas* [23]. Aromatic compounds are therefore considered stressors rather than nutrients.

In contrast to the extensive study of the metabolic regulation of PNP degradation, the transcriptional regulation of pollutant degradation has been largely neglected. *Pseudomonas putida* DLL-E4 utilizes PNP as a sole source of carbon, nitrogen, and energy. PNP degradation is PNP inducible. Because microorganisms can adapt to different environments, the study of the regulation of PNP degradation in the PNP-degrading strain *P*. *putida* DLL-E4 is necessary. In the present study, the transcriptome profiles of the wild-type strain DLL-E4 and the *pnpR*-deleted mutant strain DLL-Δ*pnpR* were subjected to comparative analysis by RNA-Seq. Based on the transcriptome analysis results, PNP gene clusters and crucial genes involved in PNP degradation were further investigated. The results of this study are meaningful for the elucidation of the regulation of PNP degradation.

## Materials and Methods

### Primers, strains, plasmids, and culture conditions

The oligonucleotide primers, bacterial strains, and plasmids used in this study are listed in S1 and S2 Tables, respectively. *P*. *putida* strains were grown at 30°C in Lysogeny Broth (LB) medium including tryptone (10 g/L), yeast extract (5 g/L), and NaCl (10 g/L), pH 7.2, or in minimal medium containing K_2_HPO_4_ (1.5 g/L), KH_2_PO_4_ (0.5 g/L), (NH_4_)_2_SO_4_ (1.0 g/L), MgSO_4_ (0.03 g/L), and NaCl (1.0 g/L), pH 7.0. *E*. *coli* strains were grown in LB medium at 37°C. Ampicillin, kanamycin, gentamicin, and chloramphenicol were added to the medium at concentrations of 100, 50, 30, and 30 μg.mL^-1^, respectively. For RNA extraction, glucose utilization, PNP degradation, and HQ degradation assays, *P*. *putida* strains were pregrown overnight in LB medium or minimal medium, collected, and washed twice with minimal medium without any added C source, concentrated to an optical density at 600 nm (OD_600_) of *ca*. 2, and inoculated into fresh minimal medium containing 0.25% (w/v) glucose, 0.5 mM PNP, or 0.5 mM HQ as the sole carbon source or containing 0.25% (w/v) glucose plus 0.5 mM PNP or 0.5 mM HQ. Each culture was then incubated at 30°C and 180 r/min until the substrate was degraded thoroughly.

### RNA manipulation and deep sequencing of transcripts

Cells were harvested at 4 h (half of PNP was degraded) for the extraction of total RNA. Total RNA was extracted using a High Pure RNA isolation kit (Roche Co., Ltd.), and RNase-free DNase (TaKaRa Co., Ltd.) treatment was performed to eliminate any residual DNA. The quality of the RNA samples was evaluated in an Agilent 2100 bioanalyzer (Agilent Technologies). RNA library construction and sequencing were performed by Hanyu Bio-Tech (Shanghai, China) using a NEBNext^®^ UltraTM RNA library prep kit and an Illumina HiSeq^™^ 2500 system (Illumina). Reads generated by the sequencing machines were cleaned and mapped to the genome of strain DLL-E4 (NCBI reference sequence NZ_CP007620.1). Comparative analysis of the transcriptomes was performed as described by Nikel *et al*. [24]. Genes with false discovery rates ≤ 0.001 and absolute fold change larger than two were considered differentially expressed.

### Quantitative reverse transcription polymerase chain reaction

Eight genes (*pnpR*, *pnpC1*, *pnpB*, *pnpA*, *pnpC1b*, *pnpC2b*, *pnpDb*, and *pnpAb*) were chosen to confirm the transcriptomes results by quantitative reverse transcription polymerase chain reaction (qRT-PCR). The culture conditions for the strains are described below. DLL-A-*aph* (*pnpR*, *pnpC1*, and *pnpC1b*) was grown on 0.25% (w/v) glucose or on 0.5 mM PNP overnight; DLL-E4 (*pnpC1*, *pnpC1b*, *crc*, *crcY*, and *crcZ*) was cultivated with 0.5 mM HQ or 0.25% (w/v) glucose plus 0.5 mM HQ; DLL-E4 (*pnpA*, *pnpAb*, *crc*, *crcY*, and *crcZ*) was grown on 0.5 mM PNP or on 0.25% (w/v) glucose plus 0.5 mM PNP. Total RNA extraction and genomic DNA elimination were performed as described above. First-strand cDNAs were obtained from 3 milligram of total RNA using a RevertAid First Strand cDNA synthesis kit (Fermentas). qRT-PCR was performed with Power SYBR^®^ Green PCR master mix (Invitrogen) according to the manufacturer’s instructions using a StepOne^™^ Real-Time PCR system (Applied Biosystems). Each reaction was performed in triplicate. The relative expression levels of selected genes were calculated via the 2^-ΔΔCt^ method using the 16S rRNA gene as an internal control.

### PNP degradation, HQ catabolism, and glucose utilization assays

*P*. *putida* strains were prepared as described above and inoculated into minimal medium containing different substrates: 0.5 mM PNP (DLL-E4, DLL-Δ*pnpR*, DLL-Δ*ins2*, DLL-Δ*pnpRR1*, DLL-d*pnpR1*, DLL-A-*aph*, DLL-A-*aph* (pBBA)), 0.5 mM HQ (DLL-E4, DLL-Δ*pnpR*, DLL-Δ*pnpRR1*, DLL-d*pnpR1*, DLL-Δ*pnpRC1*, DLL-Δ*pnpRC1b*, DLL-A-*aph*), 0.25% glucose (DLL-E4, DLL-Δ*pnpR*, DLL-A-*aph*), 0.25% glucose plus 0.5 mM PNP (DLL-E4, DLL-Δ*pnpR*), and 0.25% glucose plus 0.5 mM HQ (DLL-E4). The strains were then incubated at 180 r/min and 30°C. PNP and HQ concentrations and OD_600_ values were determined as described by Shen *et al*. [9], and glucose concentrations were determined using an improved dinitrosalicylic acid reagent every 3 h [25].

### Construction of *ins2* deletion and *pnpR1*, *pnpC1*, and *pnpC1b* disruption mutant strains

The deletion mutant strain and disruption mutant strains were constructed as described by Shen *et al*. [9]. A 1.1kb fragment was constructed to delete the entire sequence of the *ins2* gene. This fragment was excised from a PCR product with XbaI and SacI and then inserted using the same restriction sites into the suicide plasmid pJQ200SK, producing pJQ-*ins2*. Plasmid pJQ-*ins2* was transferred into *P*. *putida* DLL-E4 by homologous recombination. Colonies with single recombination events were selected based on resistance to ampicillin and gentamicin on LB plates, and colonies with double recombination events were selected after growth on LB plates containing 10% sucrose (w/v). The *ins2* deletion mutant strain was then verified multiple times by PCR. The construction of the *pnpR1*, *pnpC1* and *pnpC1b* disruption mutant strains was similar to the construction of the *ins2* deletion mutant strain, but only colonies with single recombination events were selected.

### Expression, purification and enzymatic assays of PnpA, PnpAb, PnpC1C2 and PnpC1C2b

The strains and plasmids used in this study are listed in S2 Table. *E*. *coli* BL21 (DE3) cells containing pET-*pnpA*his, pET-*pnpAb*his, pET-*pnpC1C2*his, or pET-*pnpC1C2b*his vectors were grown in LB medium at 37°C to an OD_600_ of ca. 0.6, induced for 24 h at 18°C by addition of 0.2 mM IPTG, then harvested, washed, resuspended in buffer A (20 mM Tris–HCl pH 8.5, 100 mM sodium chloride, 10 mM imidazole, 10% (v/v) glycerol), and lysed by ultrasonication. The subsequent purification and enzymatic assays of PnpA, PnpAb, PnpC1C2, and PnpC1C2b were performed as described by Shen *et al*. [9].

### Statistical analysis

All experiments reported here were independently repeated at least three times, and the mean value of the corresponding parameter ± standard deviation is presented. The level of significance of the differences when comparing results was evaluated by means of a one-way analysis of variance (ANOVA) statistical test, with α = 0.05, or through the false discovery rate values as noted above.

### Accession numbers

The transcriptome data of the four samples including E4-G, E4-GP, R-G, and R-GP have been deposited in the NCBI Sequence Read Archive database under accession numbers SRP058123, SRP058124, SRP058125, and SRP058126, respectively. *Pseudomonas putida* DLL-E4 have been deposited in China Center for Type Culture Collection under collection number CCTCC AB 2015264.

## Results and Discussion

### The transcription of both metabolic and non-metabolic genes was altered during PNP metabolism

To investigate the regulation of PNP degradation, the strains DLL-E4 and DLL-Δ*pnpR* were grown on glucose and on glucose plus PNP, and RNA-Seq was used to examine their transcriptomes. Both strains showed similar expression profiles under identical conditions, and overall expression profiles changed substantially when PNP was supplied. When cells were grown on glucose plus PNP, the expression of intergenic sequences was significantly altered, and the reads of these intergenic sequences accounted for approximately 60% of the total sequenced reads (Table 1). The huge increase in intergenic sequence expression seemed to be an adjustment to help cells survive under stressful growth condition. The expression of functional genes also changed greatly in response to PNP. (i) A total of 2912 genes were significantly downregulated in DLL-E4 grown on PNP plus glucose (represented by E4-GP) compared to growth on glucose only (represented by E4-G), whereas 845 genes were upregulated in E4-GP compared to E4-G. (ii) Strain DLL-Δ*pnpR* showed a similar expression profile when grown on PNP plus glucose (represented by R-GP) compared to growth on glucose only (represented by R-G) (Fig 1A). The functional genes up- or downregulated with a 2-fold cutoff were classified and summarized in Fig 1B (see S3 and S4 Tables in the Supporting Information for a complete list). The largest group of genes was hypothetical proteins and proteins with unknown function, which was followed at a distance by genes involved in catalytic activity, metabolic processes, cellular processes, developmental processes, molecular transducer activity, transporter activity, and binding. The RNA-Seq results indicated that PNP induced a global change in the transcriptome of *P*. *putida* DLL-E4 (Fig 1B). A large number of genes involved in regulation, ribosome and RNA biosynthesis, motility and vitamin B12 synthesis were significantly down-regulated, whereas genes involved in the response to stress were up-regulated (S3 Table). Similar changes were observed in *P*. *putida* KT2440 during growth on aromatic compounds [23]. In the subsequent sections, the expression profiles of genes encoding two significantly upregulated ncRNAs, PNP degradation gene clusters, central carbon metabolism, and catabolite repression distilled from the RNA-Seq data are analyzed in detail.

**Table 1:** Mapping rates of transcriptomes to the genome of *P*. *putida* DLL-E4.

	E4-G^a^	E4-GP^b^	R-G^c^	R-GP^d^
**Genes**	79.93%	26.00%	82.19%	33.88%
**Intergenic sequnces**	10.85%	65.43%	9.51%	56.73%
**Total reads map to genome**	90.78%	91.43%	91.70%	90.61%

^a^ E4-G represents strain DLL-E4 grown on 0.25% glucose.

^b^ E4-GP represents strain DLL-E4 grown on 0.25% glucose plus 0.5 mM PNP.

^c^ R-G represents strain DLL-Δ*pnpR* grown on 0.25% glucose.

^d^ R-GP represents strain DLL-Δ*pnpR* grown on 0.25% glucose plus 0.5 mM PNP.

### The transcripts of ncRNAs significantly changed during PNP metabolism

**Fig 2:**
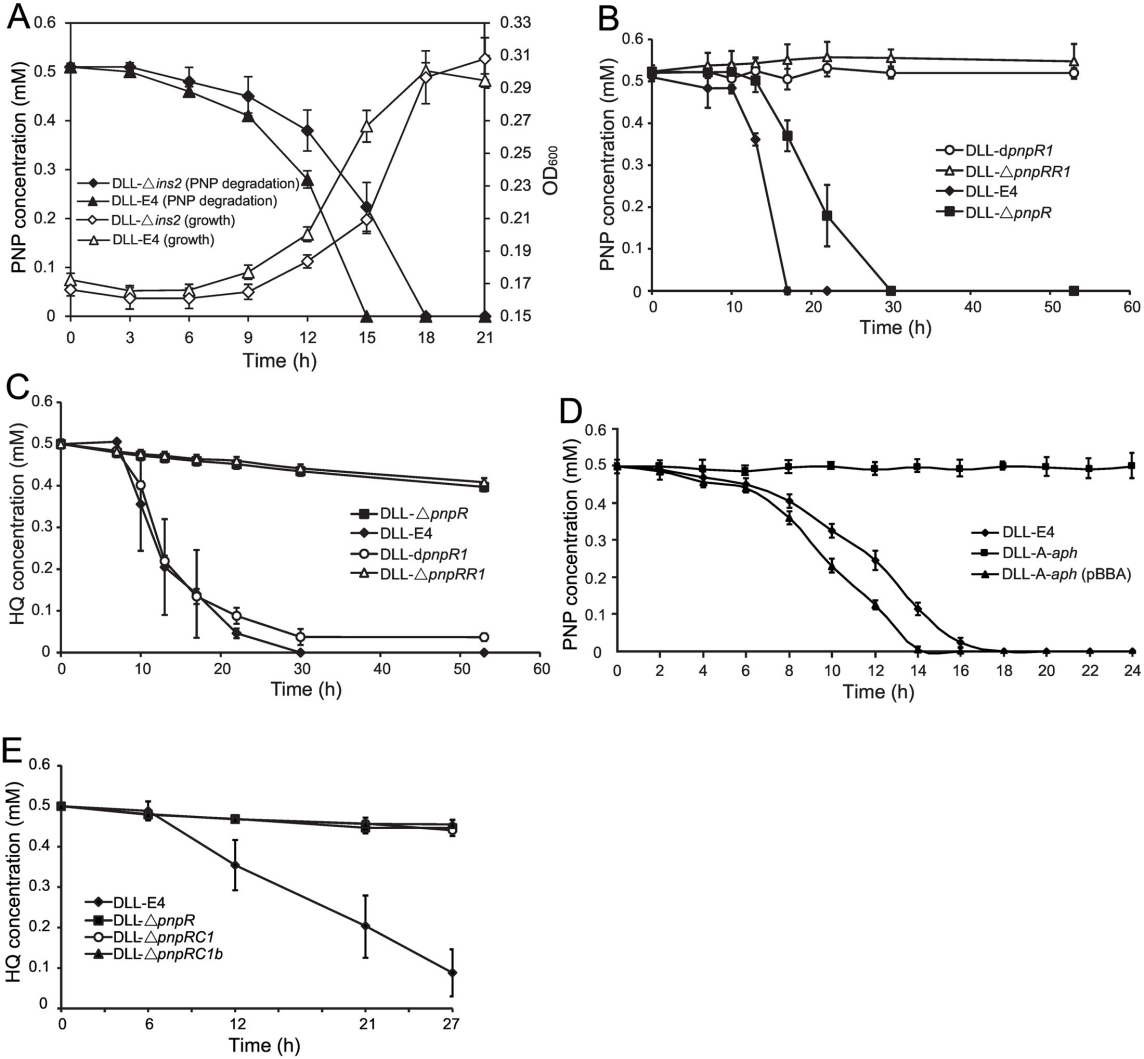
PNP degradation and HQ degradation in different *Pseudomonas putida* strains. (A) Growth curves and PNP degradation in *P*. *putida* DLL-E4 and DLL-Δ*ins2* when grown in minimal medium containing PNP as the sole carbon source. ANOVA analysis indicates different degradation rates between the tested strains at 12 h and 15 h. (B) PNP degradation in *P*. *putida* DLL-E4, DLL-Δ*pnpR*, DLL-d*pnpR1*, and DLL-Δ*pnpRR1*. (C) HQ Degradation in *P*. *putida* DLL-E4, DLL-Δ*pnpR*, DLL-d*pnpR1*, and DLL-Δ*pnpRR1*. (D) PNP degradation in *P*. *putida* DLL-E4, DLL-A-*aph*, and DLL-A-*aph* (pBBA). Strain DLL-A-*aph* (pBBA) is the complementation strain of strain DLL-A-*aph*. (E) HQ degradation in *P*. *putida* DLL-E4, DLL-Δ*pnpR*, DLL-Δ*pnpRC1*, and DLL-Δ*pnpRC1b*. Error bars represent standard deviations.

Intergenic sequences were a majority of the transcribed sequences when cells were grown on glucose plus PNP (Table 1). Two ncRNAs (named *ins1* and *ins2*) were found to account formost of these transcribed intergenic sequences (S5 Table) and were conspicuously upregulated in response to PNP (Table 2). We tried to delete *ins1* and *ins2* by homologous recombination to identify their roles on the physiological responses of strain DLL-E4 to PNP. However, *ins1* could not be deleted. *ins2* appeared to be unnecessary for cell growth and PNP degradation. Deletion of *ins2* caused slight delay of the cell growth and PNP degradation (Fig 2A). *ins1* and *ins2* were annotated to be class A bacterial ribonuclease P RNA (RNase P RNA) and transfer-messenger RNA (tmRNA), respectively. RNase P is a ubiquitous endoribonuclease that has been found in archaea, bacteria, and eukarya as well as chloroplasts and mitochondria. RNase P removes 5′ leader sequences from tRNA precursors to generate mature tRNAs for translation and is essential for cell survival [26,27]. The mechanism of RNase P RNA upregulation when cells were exposed to PNP needs further research. tmRNA is a bacterial RNA molecule with dual tRNA-like and mRNA-like properties. It recycles stalled ribosomes, adds a proteolysis-inducing tag to unfinished polypeptides, and facilitates the degradation of aberrant mRNAs during *trans*-translation. Bacteria require it to survive under stressful growth conditions [28,29]. The translation machinery of *P*. *putida* DLL-E4 was significantly decreased in the presence of PNP (S3 Table). We deduced that substantially increased expression of *ins2* allowed the cells to remedy the increased aberrant translation caused by PNP exposure. Although the two ncRNAs seemed unnecessary for PNP degradation, *ins1* and *ins2* were upregulated in response to PNP.

**Table 2:** Expression profiles of genes involved in two ncRNAs and catabolite repression in *P*. *putida* DLL-E4 and DLL-Δ*pnpR*.

Gene ID	Gene name	Function	E4-GP vs E4-G^a^ Fold change (log_2_)	R-GP vs R-G^b^ Fold change (log_2_)
**ncRNA-214**	*ins1*	class A bacterial ribonuclease P RNA (RNase P RNA)	2.83	3.71
**ncRNA-223**	*ins2*	a transfer-messenger RNA (tmRNA)	3.33	3.37
**DW66_5711**	*crc*	catabolite repression control protein Crc, exert catabolite repression	1.12	0.82
**ncRNA-134**	*crcY*	Crc-antagonist sRNA crcY, bind to and sequester Crc	3.74	5.86
**ncRNA-219**	*crcZ*	Crc-antagonist sRNA crcZ, bind to and sequester Crc	4.78	5.95

^a^ Fold changes in expression levels in strain DLL-E4 grown on 0.25% glucose plus 0.5 mM PNP compared to 0.25% glucose.

^b^ Fold changes in expression levels in strain DLL-Δ*pnpR* grown on 0.25% glucose plus 0.5 mM PNP compared to 0.25% glucose.

Values below -1 represent downregulation between the tested conditions, values above 1 represent upregulation between the tested conditions, and values between -1 and 1 indicate no differential expression between the tested conditions.

### The transcription of genes involved in PNP degradation was complex in response to PNP and *pnpR* deletion

First, we focused on transcriptional changes in genes involved in PNP degradation. In *P*. *putida* DLL-E4 [12], PNP degradation genes are made of two HQ degradation operons (*pnpC1C2DECX1X2* and *pnpC1bC2bDbEbCbX1bX2b*) and the 4 independent genes *pnpR*, *pnpA*, *pnpB*, and *pnpAb* (S1 Fig, Fig 1C and 1D). The *pnpR* and *pnpC1C2DECX1X2* operons have been shown to be involved in HQ degradation, and PnpR is a LysR-type transcription factor regulating the expression of the *pnpC1C2DECX1X2* operon [9]. *pnpR* deletion prevents strain DLL-E4 from degrading HQ [9]. The *pnpR* and *pnpC1C2DECX1X2* operons play key roles in the degradation of HQ in *P*. *putida* DLL-E4. The *pnpC1bC2bDbEbCbX1bX2b* operon is adjacent to several mobile elements, suggesting that it might have arisen from lateral gene transfer (Fig 1C). Except for *pnpR*, other PNP degradation genes were significantly upregulated during PNP degradation, which was confirmed by qRT-PCR. Nonetheless, the transcriptional increase for each gene was different. *pnpA* and *pnpB* induction was the highest and second highest, respectively, and the transcript increase from the *pnpC1C2DECX1X2* operon was higher than the *pnpC1bC2bDbEbCbX1bX2b* operon. *pnpR* and *pnpAb* levels were unaffected. However, *pnpB* upregulation was highest and *pnpA* was lowest when *pnpR* was deleted, and the transcriptional increase from the *pnpC1C2DECX1X2* operon was less than from *pnpC1bC2bDbEbCbX1bX2b* operon. Furthermore, *pnpAb* was upregulated 26-fold after *pnpR* deletion (Table 3).

**Table 3:** Expression profiles of the two PNP degradation gene clusters in *P*. *putida* DLL-E4 and DLL-Δ*pnpR*.

Gene ID	Gene name	Function	E4-GP vs E4-G fold change (ratio^a^) RNA-Seq qPCR	R-GP vs R-G fold change (ratio^b^) RNA-Seq qPCR	R-GP vs E4-GP fold change (ratio^c^) RNA-Seq qPCR
**DW66_3572**	*pnpA*	*para*-nitrophenol 4-monooxygenase	519.1 152.7	7.1 2.1	0.02 0.003
**DW66_3573**	*pnpB*	1,4-benzoquinone reductase	306.1 47.6	610.8 298.2	6.9 17.7
**DW66_3577**	*pnpE*	maleylacetate reductase	32.3	12.7	0.4
**DW66_3578**	*pnpD*	4-hydroxymuconic semial-dehydrogenase	23.2	12.7	0.6
**DW66_3579**	*pnpC2*	hydroquinone hydroxylase beta subunit	35.4	30.7	0.5
**DW66_3580**	*pnpC1*	hydroquinone dioxygenase alpha subunit	25.7 10.3	21.6 17.2	0.4 0.6
**DW66_3581**	*pnpR*	putative LysR family transcriptional regulator	1.0 1.0	-^d^-	- -
**DW66_3565**	*pnpAb*	monooxygenase, FAD-binding	1.4 1.2	26.2 9.9	23.1 36.4
**DW66_3594**	*pnpEb*	putative maleylacetate reductase	6.5	7.9	1.6
**DW66_3595**	*pnpDb*	*gamma*-hydroxymuconic semialdehyde dehydrogenase	5.2 2.1	11.4 9.0	2.9 5.4
**DW66_3596**	*pnpC2b*	hydroquinone dioxygenase large subunit	16.3 2.5	43.8 13.5	2.8 6.8
**DW66_3597**	*pnpC1b*	hydroquinone dioxygenase small subunit	26.2 12.4	65.3 36.4	2.8 5.2

^a^ Fold changes in expression levels in strain DLL-E4 grown on 0.25% glucose plus 0.5 mM PNP compared to 0.25% glucose.

^b^ Fold changes in expression levels in strain DLL-Δ*pnpR* grown on 0.25% glucose plus 0.5 mM PNP compared to 0.25% glucose.

^c^ Fold changes in expression levels in strain DLL-Δ*pnpR* grown on 0.25% glucose plus 0.5 mM PNP compared to strain DLL-E4 grown on 0.25% glucose plus 0.5 mM PNP.

^d^ Hyphens indicate that gene is not transcribed.

Values below 0.5 represent downregulation between the tested conditions, values above 2 represent upregulation between the tested conditions, and values between 0.5 and 2 indicate no differential expression between the tested conditions.

When grown on glucose plus PNP, *pnpA* and *pnpB* genes, which encode enzymes that transform PNP into HQ, responded most robustly to induction, with increases of 519- and 306-fold, respectively. However, the expression of *pnpA* was only increased 7-fold under identical conditions when *pnpR* was deleted, indicating that *pnpR* is involved in the positive regulation of *pnpA*. Insertional inactivation of another LysR-type regulator, PnpR1 (DW66_3566), led to complete repression of PNP degradation (Fig 2B); however, the degradation rate of HQ was unaffected (Fig 2C). PnpR1 shares 85% identity with the *pnpA* regulator PnpR_wbc_ from *Pseudomonas* sp. strain WBC-3 [19]. It has been suggested that *pnpA* might be regulated by multiple transcriptional factors.

The expression levels of the *pnpC1C2DECX1X2* and *pnpC1bC2bDbEbCbX1bX2b* operons were significantly increased in both DLL-E4 and DLL-Δ*pnpR* strains during PNP degradation (Table 3). However, they showed opposite responses to *pnpR* deletion. The expression levels from the *pnpC1C2DECX1X2* operon in strain DLL-Δ*pnpR* were half of the expression in strain DLL-E4, whereas the expression levels from *pnpC1bC2bDbEbCbX1bX2b* operon were approximately 2-fold higher in strain DLL-Δ*pnpR* than in strain DLL-E4. It was proposed that the *pnpC1C2DECX1X2* operon was positively regulated by *pnpR* and that the *pnpC1bC2bDbEbCbX1bX2b* operon might be negatively regulated by *pnpR* or other unknown factors.

### *pnpA* rather than *pnpAb* was the key gene in the initial step of PNP degradation

**Fig 3:**
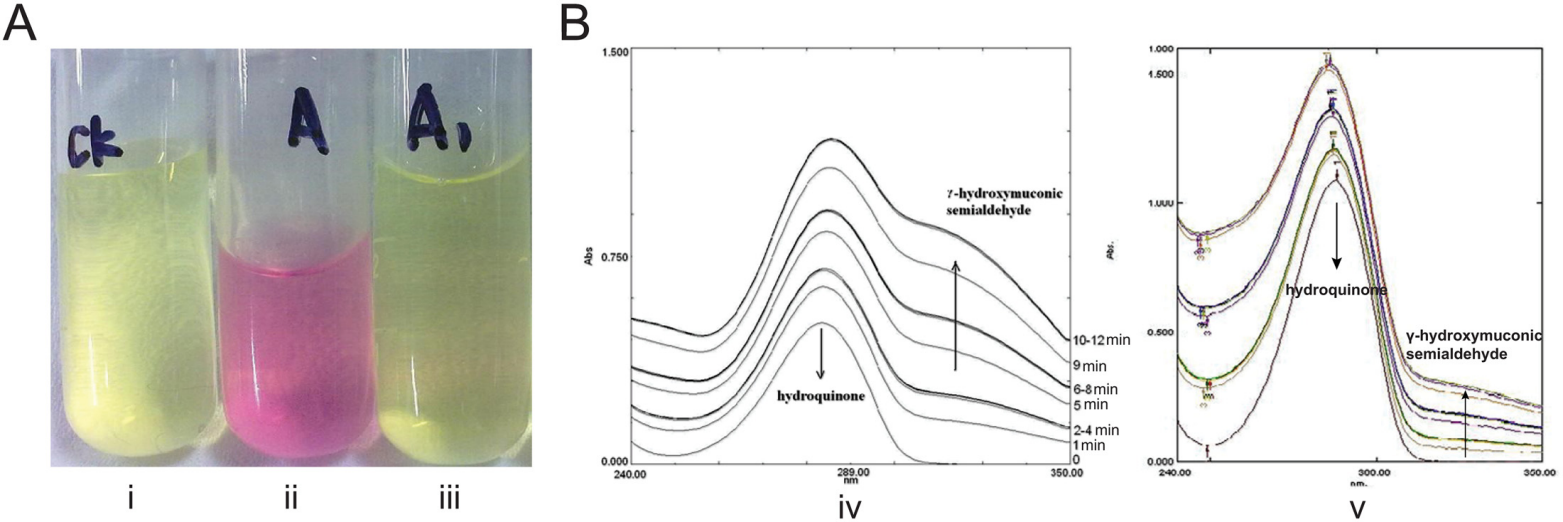
Enzymatic assays for PnpA, PnpAb, PnpC1C2, and PnpC1C2b. (A) Enzymatic assays for PnpA and PnpAb. Yellow represents PNP that was not oxidized, pink represents PNP that was oxidized to other compounds. i: Negative control; ii: PNP degraded by PnpA; iii: PNP degraded by PnpAb. (B) Enzymatic assays for PnpC1C2 and PnpC1C2b. HQ has an absorption peak at 288 nm, and γ-hydroxymuconic semialdehyde shows absorption at 288–320 nm and an absorption peak at 320 nm. When HQ is transformed into γ-hydroxymuconic semialdehyde, the absorption at 320 nm increases. iv: Spectral changes associated with the transformation of HQ by PnpC1C2. v: Spectral changes associated with the transformation of HQ by PnpC1C2b.

*pnpA* exhibits 59.2% identity with *pnpAb*, and both of them encode a flavin adenine dinucleotide-dependent single-component PNP 4-monooxygenase that converts PNP to *para*-benzoquinone. By expressing and purifying the PnpA and PnpAb proteins and performing enzymatic assays, we discovered that only PnpA had the ability to oxidize PNP and that PnpAb did not contribute to PNP oxidation (Fig 3A). A *pnpA* disruption mutant strain, DLL-A-*aph*, encodes inactivated *pnpA* and a wild-type *pnpAb* and cannot degrade PNP but can still degrade HQ. When the complete *pnpA* sequence was expressed in strain DLL-A-*aph*, the ability to degrade PNP was recovered, and the degradation rate was close to that of the wild-type strain DLL-E4 (Fig 2D). All of these results indicated that *pnpA* is the key gene in the initial step of PNP degradation.

### The operon *pnpC1bC2bDbEbCbX1bX2b* does not degrade HQ in vivo

*pnpC1C2* and *pnpC1C2b* were heterologously expressed in *E*. *coil* BL21(DE3), and the C-terminal His-tagged recombinant proteins PnpC1C2 and PnpC1C2b were purified using Ni^2+^-nitri-lotriacetic acid (NTA) resin (Qiagen, Valencia, CA, USA) according to the manufacturer’s instructions. The activity of the purified PnpC1C2 and PnpC1C2b was detected using HQ as the substrate. Recombinantly expressed PnpC1C2 and PnpC1C2b both transformed HQ into *γ*-hydroxymuconic semialdehyde in vitro (Fig 3B), and PnpC1C2b transformed HQ at a much lower rate. The *pnpC1C2DECX1X2-*disrupted and *pnpR*-deleted mutant strain DLL-Δ*pnpRC1* was constructed by homologous recombination. The ability of strain DLL-Δ*pnpRC1* carrying the inactivated operon *pnpC1C2DECX1X2* and the wild-type operon *pnpC1bC2bDbEbCbX1bX2b* to degrade HQ was also measured. Strain DLL-Δ*pnpRC1* was unable to degrade HQ (Fig 2E). The *pnpC1bC2bDbEbCbX1bX2b* operon shares 67.3–84.6% identity with the *pnpC1C2DECX1X2* operon and much higher identity (99%) with the *pnpCDEFG* operon, which is responsible for HQ degradation in *Pseudomonas* sp. strain WBC-3 [10]. The *pnpC1bC2bDbEbCbX1bX2b* operon encodes a complete set of genes for HQ degradation but cannot degrade HQ in vivo, indicating that a specific type of regulation inhibits its function in vivo.

### The transcriptional regulation of PNP degradation was controlled by multiple factors

**Fig 4:**
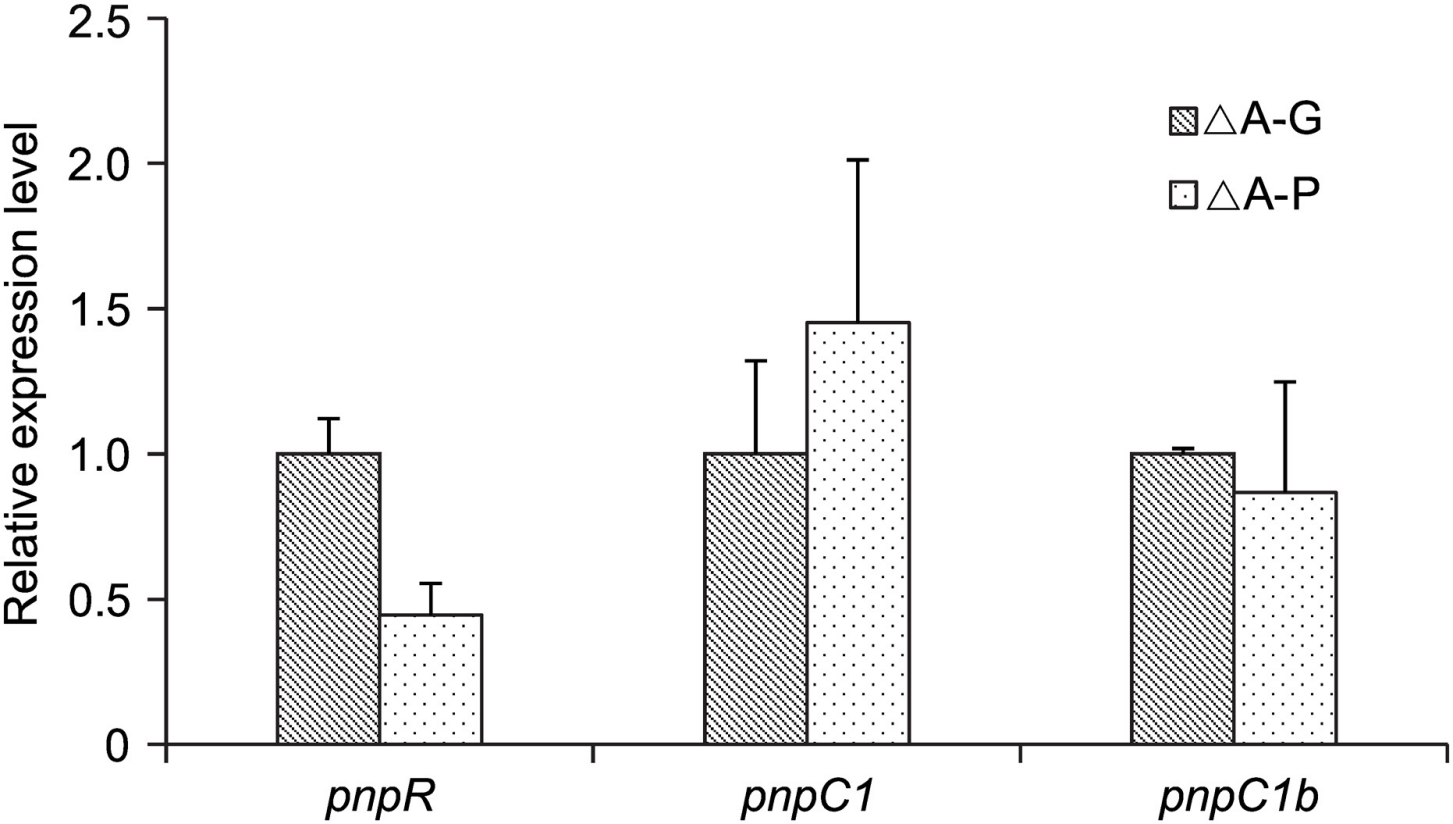
Expression of *pnpR*, *pnpC1*, and *pnpC1b* in *P*. *putida* DLL-A-*aph*. Cells were grown for 24 h and then harvested to extract total RNA. The expression levels of the three genes in treated cells grown on PNP were measured by qRT-PCR using the 16S gene as a reference gene and calculating via the 2^-ΔΔCt^ method; The expression level of each gene in reference cells grown on glucose was regarded as 1. ANOVA analysis indicates no differential expression between the tested conditions. The values are averages of results from three independent experiments, error bars represent the standard deviations. ΔA-G refers to a reference sample grown in minimal medium containing 0.25% glucose, while ΔA-P refers to a treated sample grown in minimal medium containing 0.5 mM PNP.

The expression levels of *pnpR*, *pnpC1*, and *pnpC1b* in the *pnpA* disruption mutant strain DLL-A-*aph* were measured by qRT-PCR when grown on PNP or glucose as the sole carbon source (Fig 4). The qRT-PCR results indicated that the selected genes were expressed at basal levels without the addition of PNP and that their expression levels were altered slightly when PNP was supplied. The expression of the *pnpR*, *pnpC1C2DECX1X2*, and *pnpC1bC2bDbEbCbX1bX2b* operons were not induced by PNP when *pnpA* was inactivated, suggesting that the effector of the *pnpR*, *pnpC1C2DECX1X2*, and *pnpC1bC2bDbEbCbX1bX2b* operons is not PNP and that the significantly upregulated expression of *pnpC1C2DECX1X2* and *pnpC1bC2bDbEbCbX1bX2b* by RNA-Seq results might be a response to HQ generated by PNP oxidation.

The transcriptional regulation of PNP degradation in strain DLL-E4 was complex (Fig 1C) and different from strain WBC-3 [19]. The *pnpR*, *pnpA*, *pnpB*, and *pnpCDEFG* operons were induced by PNP in the WBC-3 strain. However, PNP was the effector of *pnpA* and HQ was the effector of *pnpC1C2DECX1X2*, and *pnpC1bC2bDbEbCbX1bX2b* operons in strain DLL-E4. The transcriptional level of *pnpA* was enhanced dramatically in the presence of PNP, and *pnpC1C2DECX1X2* and *pnpC1bC2bDbEbCbX1bX2b* were unaltered when *pnpA* was inactivated even in the presence of PNP (Fig 4). A LysR-type transcriptional regulator, PnpRwbc, shares 45% identity with PnpR and positively regulates the expression of the *pnpA*, *pnpB*, and *pnpCDEFG* operons in strain WBC-3. *pnpA*, *pnpB*, and *pnpCDEFG* in strain WBC-3 shared 81%, 84%, and 99% identity with *pnpA*, *pnpB*, and *pnpC1bC2bDbEbCbX1bX2b*, respectively in DLL-E4. In strain DLL-E4, *pnpA* was positively regulated by PnpR and PnpR1, and *pnpC1C2DECX1X2* was positively regulated by PnpR and not regulated by PnpR1. However, the transcriptional regulator of *pnpAb* and *pnpC1bC2bDbEbCbX1bX2b* remains unknown.

*P*. *putida* DLL-E4 has two gene clusters for PNP degradation. However, only *pnpA*, *pnpR*, *pnpC1C2DECX1X2*, *and pnpR1* were key genes in PNP degradation. Although the *pnpAb* and *pnpC1bC2bDbEbCbX1bX2b* operons lost the ability to degrade PNP, they were still upregulated in the presence of PNP, indicating that other unknown factors regulate them.

### Glucose utilization was first enhanced and then inhibited when PNP was provided

**Fig 5:**
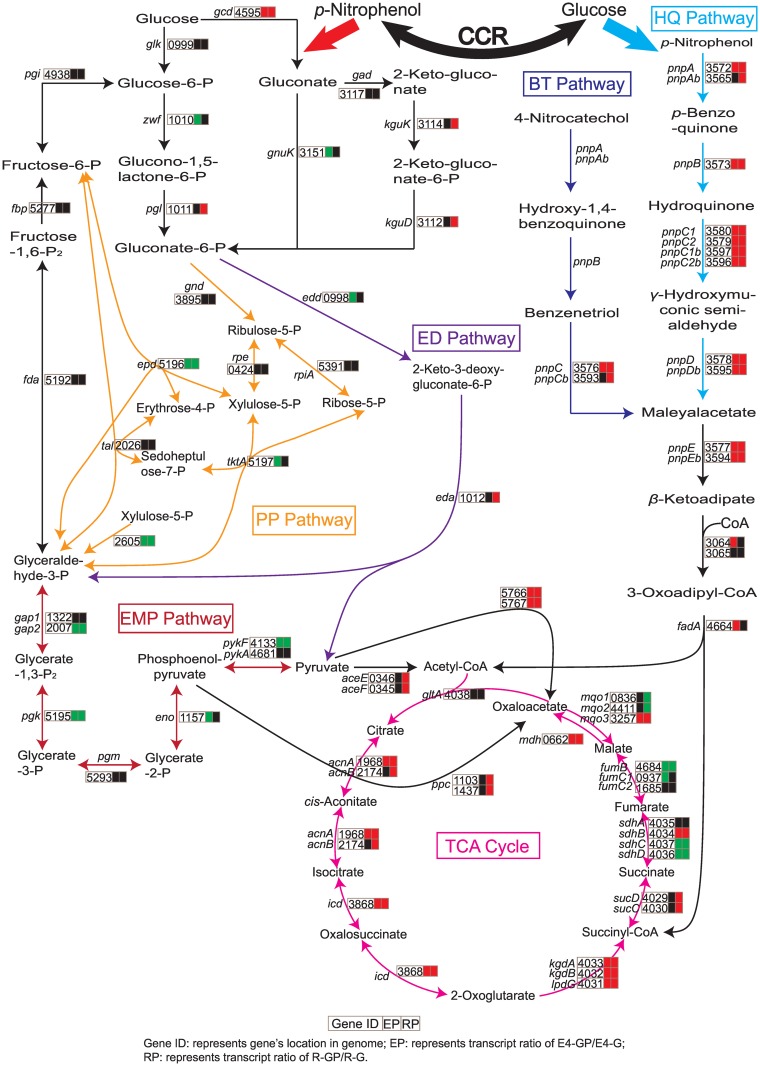
Differential expression of genes involved in upstream central carbon metabolism and PNP catabolism according to the comparative transcriptome analysis. The biochemical network schematically sketches the main bioreactions involved in C processing and PNP hydrolysis. The conditions mentioned in this figure are identical to the previous conditions used for the RNA-Seq samples (E4-GP vs E4-G, and R-GP vs R-G, as described in the legend to Fig 1). Genes encoding enzymes involved in these bioreactions are highlighted in different colors according to whether they were significantly upregulated (red) or downregulated (green). The red heavy arrow represents that glucose utilization could be enhanced when PNP was present. The blue heavy arrow represents that glucose prompts the degradation of PNP. The black heavy double-arrow indicates that PNP degradation and glucose catabolism are regulated by carbon catabolite repression.

A network chart is presented here according to the comparative transcriptome analysis and reported carbon metabolism of *P*. *putida* [30] (Fig 5): (i) PNP is converted to *β*-ketoadipate through the HQ pathway and then enters into tricarboxylic acid cycle (TCA cycle). Glucose is metabolized by three simultaneous pathways that converge at 6-phosphogluconate and then enters the Entner/Doudoroff pathway or the pentose phosphate pathway to the TCA cycle [31,32]. The TCA cycle links PNP degradation and central carbon metabolism. (ii) Transcripts of genes involved in central carbon metabolism and PNP degradation changed conspicuously when cells were grown on glucose plus PNP.

**Fig 6:**
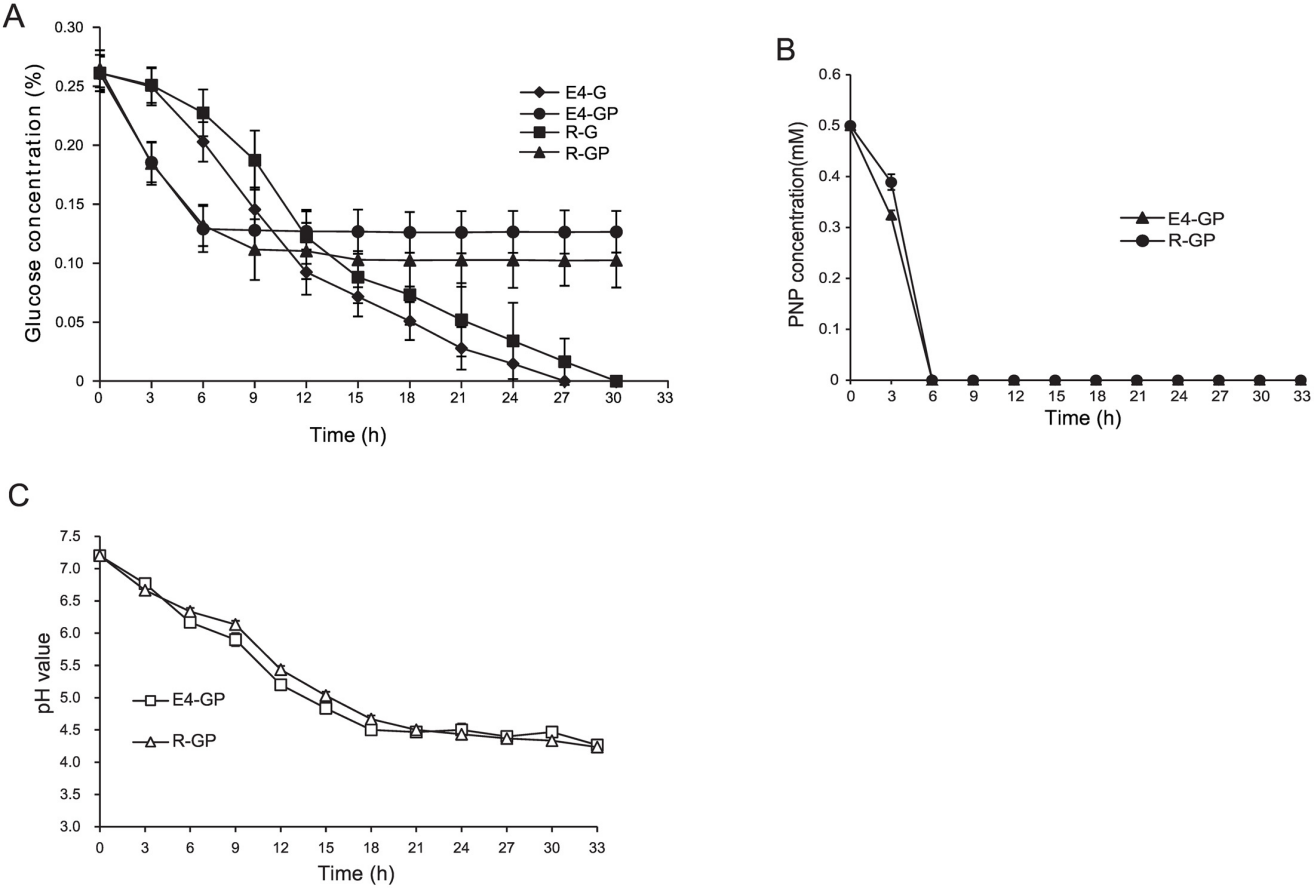
RNA-Seq samples for glucose utilization and PNP degradation. (A) Glucose utilization in each RNA-Seq sample. The concentration of residual glucose was measured with the DNS method. (B) PNP degradation in samples E4-GP and R-GP. (C) pH variation in *P*. *putida* DLL-E4 and DLL-Δ*pnpR* grown in minimal medium containing 0.5 mM PNP plus 0.25% glucose. The conditions for samples E4-G, R-G, E4-GP, and R-GP were identical to those of the RNA-Seq samples. Error bars represent standard deviations.

Transcripts from genes encoding enzymes for PNP metabolism and the TCA cycle significantly increased, whereas those from genes involved in ribosomal protein synthesis, rRNA synthesis, and RNA polymerase sigma factors clearly decreased (S6 Table) during PNP degradation. Meanwhile, genes encoding the outer membrane porin OprB and glucose dehydrogenase Gcd were upregulated (S7 Table), and genes involved in the Embden-Meyerhof pathway and the pentose phosphate pathway were unaffected or downregulated in the presence of PNP (Fig 5). Glucose utilization assays (Fig 6A) showed that the addition of PNP led to an increase in glucose utilization at early stages. The increased expression of the outer membrane porin OprB and the glucose dehydrogenase Gcd (S7 Table) might enhance glucose transport and glucose oxidation, leading to enhanced glucose utilization at early stages. However, glucose utilization ceased after the PNP was completely consumed (Fig 6A and 6B). It was proposed that the inhibition of glucose utilization might result from the downregulated expression of genes involved in ribosomal proteins synthesis, rRNA synthesis, and RNA polymerase sigma factors (S6 Table) or from the low pH (Fig 6C). The residual glucose was completely used when the pH of the medium was adjusted to its initial value (7.0), indicating that the low pH played an important role in inhibition of glucose utilization. Nevertheless, the mechanism causing the drastic drop in medium pH after the addition of PNP remains unknown and will be interesting to research further.

### PNP degradation might be regulated by carbon catabolite repression

**Fig 7:**
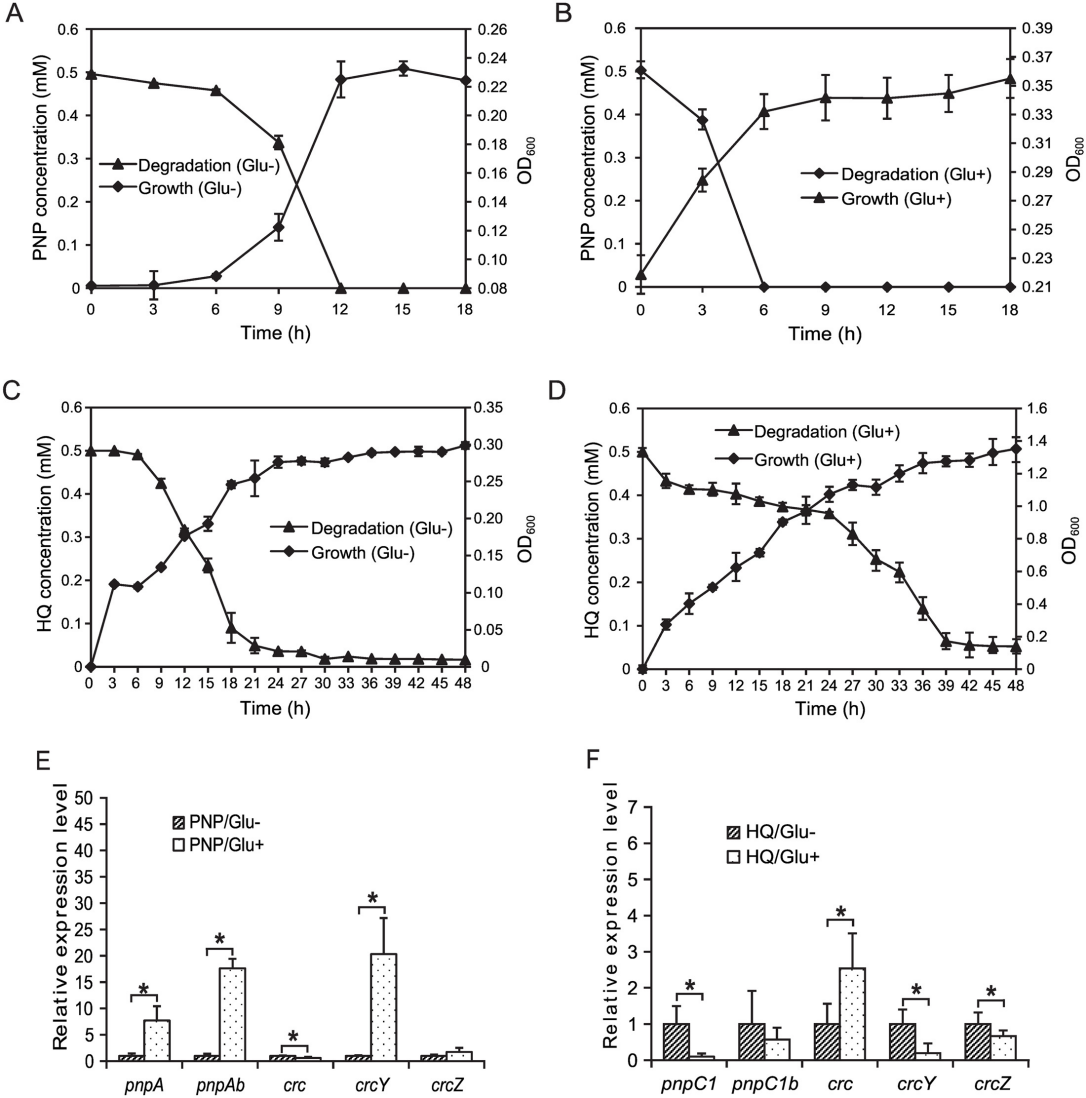
PNP and HQ degradation by *P*. *putida* DLL-E4 and the expression profiles of related functional genes involved in the process of degradation. (A) PNP degradation and cell growth in cultures of *P*. *putida* DLL-E4 without addition of glucose. (B) PNP degradation and cell growth in cultures of *P*. *putida* DLL-E4 with addition of glucose. (C) HQ degradation and cell growth in cultures of *P*. *putida* DLL-E4 without addition of glucose. (D) HQ degradation and cell growth in cultures of *P*. *putida* DLL-E4 with addition of glucose. The effects of glucose on the expression levels of functional genes in *P*. *putida* DLL-E4 during PNP degradation (E) and HQ degradation (F). The expression levels of *pnpA*, *pnpAb*, *pnpC1*, *pnpC1b*, *crc*, *crcY*, and *crcZ* were analyzed by qRT-PCR. The values are averages of the results from three independent experiments, and the error bars represent the standard deviation. *P<0.05 compared to the reference sample. Glu- denotes no glucose supplementation, whereas Glu+ denotes glucose supplementation.

When the preferred carbon source is present at a sufficient concentration, the assimilation of a non-preferred carbon source is inhibited by a complex regulatory process. This process is known as carbon catabolite repression (CCR) [21], in which the *crc*, *crcY*, and *crcZ* genes play important roles [21,22,33] in *Pseudomonas*. Transcripts from CCR-related genes changed substantially when cells were exposed to PNP and glucose (Table 2). Therefore, the expression profiles of genes involved in CCR, PNP degradation, and HQ degradation were investigated in the strain DLL-E4 with or without glucose. When glucose was supplied, PNP degradation (Fig 7A and 7B) was enhanced greatly, and HQ degradation was inhibited (Fig 7C and 7D) (the HQ assessed for HQ degradation assay was not produced by PNP degradation but was supplied artificially as a single substrate at the experimental onset). Meanwhile, the expression levels of certain genes changed (Fig 7E and 7F). *pnpA*, *pnpAb*, *crcY* and *crcZ* were upregulated, and *crc* was downregulated when grown on PNP plus glucose, whereas the expression of *pnpC1*, *pnpC1b*, *crcY* and *crcZ* decreased and that of *crc* increased when exposed to HQ and glucose. *Pseudomonas* strains optimize their growth by selectively assimilating one specific compound and avoiding less-preferred carbon sources. CCR is a common mechanism regulating the degradation of aromatic compounds in *Pseudomonas* [21,22]. The upregulation of *crcY* and *crcZ* and downregulation of *crc* relieve CCR, whereas downregulation of *crcY* and *crcZ* and upregulation of *crc* strengthen CCR [22,34]. We propose that the relief of CCR results in enhanced PNP degradation and that the upregulation of *crc* in the presence of HQ and glucose strengthens CCR and further inhibits HQ degradation. The potential regulation of PNP degradation by CCR requires further study.

## Conclusions

In the present study, the expression profile of *P*. *putida* DLL-E4 induced by PNP was investigated. *pnpA*, *pnpR*, *pnpC1C2DECX1X2*, and *pnpR1* are key genes in PNP degradation, whereas *pnpAb* and *pnpC1bC2bDbEbCbX1bX2b* have no function in the process of PNP degradation. Based on the results of RNA-Seq and qRT-PCR, the expression profile changed globally in *P*. *putida* DLL-E4 in response to the stress of PNP exposure. Non-coding RNAs (*ins1* and *ins2*) were significantly up-regulated in the presence of PNP. CCR is involved in the regulation of PNP degradation: up-regulated expression of *crc* represses the degradation of HQ. Our results indicate complex regulation of gene expression in *P*. *putida* DLL-E4 in the presence of PNP, although the regulatory network remains to be further clarified.

## Supporting Information

S1 Fig RT-PCR products for the validation of operon predictions (2.0% agarose gel). (DOCX)

S1 Table Oligonucleotide primers used in this study. (DOCX)

S2 Table Strains and plasmids used in this study. (DOCX)

S3 Table List of genes differentially expressed in *P*. *putida* DLL-Δ*pnpR* grown on glucose plus PNP compared to glucose. The fold changes are reported in log_2_-based format.
(DOCX)

S4 Table List of genes differentially expressed in *P*. *putida* DLL-E4 grown on glucose plus PNP compared to glucose. The fold changes are reported in log_2_-based format.
(DOCX)

S5 Table The proportion of two ncRNAs in the transcribed intergenic sequences. (DOCX)

S6 Table Differentially expressed genes related to ribosomal proteins synthesis, rRNA synthesis, and RNA polymerase sigma factors in *P*. *putida* DLL-E4 and DLL-Δ*pnpR*. The fold changes are reported in log_2_-based format.
(DOCX)

S7 Table Differentially expressed genes related to glucose transport and oxidization in *P*. *putida* DLL-E4 and DLL-Δ*pnpR*. The fold changes are reported in log_2_-based format.
(DOCX)
