# Supplementary material for: The Regulation of para-Nitrophenol Degradation in Pseudomonas putida DLL-E4
Source: PLoS One. 2016 May 18;11(5):e0155485. doi: 10.1371/journal.pone.0155485 (PMC4871426; doi:10.1371/journal.pone.0155485)
Supplement: S1 Fig — (DOCX) [file pone.0155485.s001.docx]

**Fig. S1**


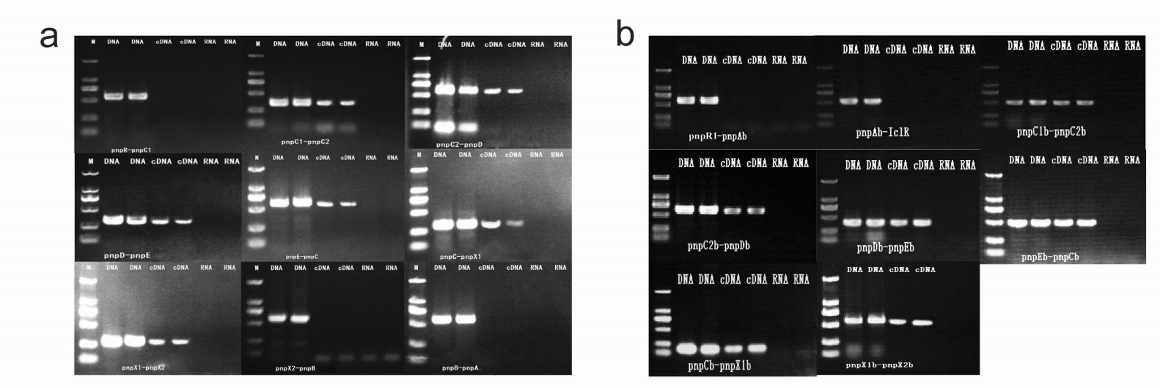


**Fig. S1.** **RT-PCR products for validation of operon predictions (2.0% agarose gel).** DNA = genomic DNA template. cDNA = cDNA template. RNA = RNA template. (a) RT-PCR results for 10 genes from the *pnp* cluster. (b) RT-PCR results for 10 genes from the *pnp1* cluster.
