# Supplementary material for: The Regulation of para-Nitrophenol Degradation in Pseudomonas putida DLL-E4
Source: PLoS One. 2016 May 18;11(5):e0155485. doi: 10.1371/journal.pone.0155485 (PMC4871426; doi:10.1371/journal.pone.0155485)
Supplement: S1 Table — (DOCX) [file pone.0155485.s002.docx]

**Table S1. Oligonucleotide primers used in this study.**

| **Primer** | **Sequence (5’ to 3’)** |
| --- | --- |
| **For qRT-PCR** | |
| *pnpR*-497F  *pnpR*-685R  *pnpC1*-277F  *pnpC1*-456R  *pnpB*-275F  *pnpB*-441R  *pnpA*-539F  *pnpA*-742R  *pnpC1b*-117F  *pnpC1b*-279R  *pnpC2b*-565F  *pnpC2b*-811R  *pnpDb*-987F  *pnpDb*-1180R  *pnpAb*-514F  *pnpAb*-719R  16S-1310F  16S-1466R  *crc*-111F1  *crc*-273R1  *crcZ*-32F1  *crcZ*-186R1  *crcY*-64F2  *crcY*-267R2  **For Disruption**  *pnpC1*-F  *pnpC1*-R  C1T (712) F  C1T (1702) R  C1b1777F  C1b2807R  pJQ51F  pJQ383R  C1b5754F  C1b6936R  *ins2*-F-TYB-320F  *ins2*-F-TYB-789R  *ins2*-R-TYB-1200F13  *ins2*-R-TYB-1851R13  *ins2*-YZ1-140F  *ins2*-YZ1-1892R  *ins2*-YZ2-30F  *ins2*-YZ2-360R  3566-F  3566-R  pJQMCS-3566F  pJQMCS-3566R  3566T-F  3566T-R | GCATGTCACCCCAATACGC  AATCGCGCAACAGGTGAAC  CTAGACACACCTTTGGCAG  CGGACCTTTGATGGTTTGC  ATGAACACGCTACCCACC  TCCATCCCTTATGCCACG  GGAAAATCGGCCTGACGT  CTTCAGCCTCGGAGATGT  CGCTCAGAACTCCAAACCC  GAGCTTGAGGAACTCCACA  CCGGTCAATCGCCAGTTT  TTTCGTCGGACATCTGGA  CGACCGTGTACTGAGCTAC  AGCTGAAACGCACCACGGT  AAGCATCTGGGGGTGAGT  ATCTCGGCCTCGGACAAT  GCAACTCGACTGCGTGAA  ACCGTGGTAACCGTCCTC  ACTCGATGACCCAGCTTTCC  TTGCAGGTAACGCCCGTAG  CAAGACGAAACGGCTCACGC  TCGATCCAACCAGTTCAGGC  CAGAGGCGTAGCAAGCAGAT  TTGTCAGCCTGTCCTGGTG  TCTTGTTCTCGGAAGGGATTA  ACTGTTGTCGTCGCCTGTGTA  TTGCGAGTACCGATGACGC  TCGGCGAGGAACCCAGTT  TGATCGTCAAAAACGGCCGGTCG  AGGAAAATGTCGGCGGGAATCAG  GTTTTCCCAGTCACGACGTTGTA  ACCTCACTCATTAGGCACCCCAG  CATCAAGGCTCGCAGGAA  ATCAAGCGCGGTTACTGG  TCTAGAGCCATCCCGATTGAA  CTCTTGGCTAACGCACCTTA  GTTAGCCAAGAGTCTAAAGACGTCCACG  GAGCTCAGTAGATTGATGACTTGCGGTG  ACCTGATAGCTGCCGCAATC  GCTTCACGGTTTCAAGGCTG  CGGTAGCGAAACTCTAGGTG  CGCTGTCGGTACTACATGCT  ATTACACCGATTGCCAG  GTATCGGTCTGGTAGTCC  TTTTCCCAGTCACGACGT  CACTCATTAGGCACCCCA  GCCCTGTTGTTTTGTCG  CGGGTAGTAACGGGAGAT |
| **For validation of operon prediction** | |
| pnp-*orf2*-1034F | AGAAATGGTGGCGAGTATCC |
| pnp-R-1623R | ATGAACTGCTCCAACTGCTG |
| pnp-R-2252F | ATTCCGGTGTTGCCAGAGTAC |
| pnp-C1-2831R | TGCCAAAGGTGTGTCTAGCT |
| pnp-C1-2978F  pnp-C2-3323R  pnp-C2-3907F  pnp-D-4516R  pnp-D-5356F  pnp-E-5684R  pnp-E-6381F  pnp-C-7001R  pnp-C-7392F  pnp-X1-7710R  pnp-X1-7738F2  pnp-X2-8287R2  pnp-X2-8177F  pnp-B-8775R  pnp-B-9041F  pnp-A-9611R  pnp-A-10560F  pnp-*orf3*-11177R  pnp-Ab-20558F  pnp-*iclR*-21077R  pnp-*pnpR1*-19135F  pnp-Ab-19741R  pnp-C*1*b-2178F  pnp-C*2*b-2679R  pnp-C*2*b-3204F  pnp-Db-3823R  pnp-Db-4880F  pnp-Eb-5283R  pnp-Eb-6062F  pnp-Cb-6574R  pnp-Cb-7122F  pnp-X*1*b-7300R  pnp-X*1*b-7228F  pnp-X*2*b-7825R | AACCATCAAAGGTCCGCTGT  AGCCATAGAAAAAGCCCCAG  CGACAAGGACGAAGGCAATC  AAGTAACGGAAGCAGGCAGC  TTGAAACCGACGAAGAAGCG  TCGGCAGGCTTTGGTAGACA  ATACACTCGGCGGCACATTC  ACCTTGCCGTCACACTTCTG  TGTAGGCGAGATGCTTGACG  AGAGCCTTCATTGTCGGTGG  ACCCACAGAGCCCATTTGCGA  TCCACCCTTACCTCCACACGG  CAAAGTCCTGATGTGTGCCC  CAGGAAACCACCATCACCAG  AGCAGCGTGACCTCAACATC  GACAACGACCACACCCTCAA  AAGGTGCTGGACGAGTATTC  GTGTCGATCTGAAAAGCCTG  GCTGACCGAACTCAACGAA  CGATAGCATCAAGGAAGCG  TGGTGATGACCGACTTTGC  CAGGATGAGGGTGAAACGT  CGCTCAGAACTCCAAACCC  ATGGGGTGGACTGGCTTTC  GGACGAGCCGGAAGTTCAT  CAATGCGGGTGATCAACGA  TCACCGTGGTGCGTTTCAG  CCAGGCGTTCGATCTCTTC  GGTGGGTGCATTGTTGTAC  GAGGGTGTCCGACAACAAA  ACGATGATTTGTCCGCCGA  TGAACGCAAATGCGCCTGA  TGCCACCGATCATCCCGAT  CCAGGCGTATTGGCACTGA |
