# Supplementary material for: The Regulation of para-Nitrophenol Degradation in Pseudomonas putida DLL-E4
Source: PLoS One. 2016 May 18;11(5):e0155485. doi: 10.1371/journal.pone.0155485 (PMC4871426; doi:10.1371/journal.pone.0155485)
Supplement: S2 Table — (DOCX) [file pone.0155485.s003.docx]

**Table S2. Strains and plasmids used in this study.**

| **Strains and plasmids** | **Characteristic(s)** | **Source** |
| --- | --- | --- |
| Strains： |  |  |
| *Pseudomonas putida*  DLL-E4  DLL-△*pnpR*  DLL-△*pnpRC1*  DLL-△*pnpRC1b*  DLL-d*pnpR1*  DLL-△*pnpRR1*  DLL-△*ins2*  DLL-A-*aph*  DLL-A-*aph* (pBBA) | Wild type; PNP degrader, G^-^, Amp^r^, Cm^r^  *pnpR* gene-deletion mutant of DLL-E4  *pnpC1* disruption mutant of DLL-△*pnpR*  *pnpC1b* disruption mutant of DLL-△*pnpR*  *pnpR1* disruption mutant of DLL-E4  *pnpR1* disruption mutant of DLL-△*pnpR*  *ins2* gene-deletion mutant of DLL-E4  *pnpA* replacement mutant of DLL-E4  complementation for *pnpA* replacement mutant of DLL-E4 | Lab stock  Lab stock  This study  This study  This study  This study  This study  Lab stock  Lab stock |
| *Escherichi coli*  DH5α | Host strain for cloning vectors | Lab stock |
| SM10_λpir_ | Conjugation strain | Lab stock |
| BL21 | F_ *ompT hsdS*(rB mB) *dcm lacY1*(DE3) | Lab stock |
| Plasmids |  |  |
| pJQ200SK | Gm^r^, *ori*p15A, Mob^+^, *lac*Zα^+^, *sacB* | Lab stock |
| pRK2013 | Helper plasmid, mob^+^, tra^+^, Kan^r^ | Lab stock |
| pJQ-*pnpC1*  pJQ-*pnpC1b*  pJQ-*ins2*  pJQ-*pnpR1*  pMD19-T  pET-*pnpA*his  pET-*pnpAb*his  pET-*pnpC1C2*his  pET-*pnpC1C2b*his | *pnpC1* insertion construct in pJQ200SK  *pnpC1b* insertion construct in pJQ200SK  *ins2* deletion construct in pJQ200SK  *pnpR1* insertion construct in pJQ200SK  T-A clone vectors, Amp^r^  pET-29a(+) derivative carrying the *pnpA*  pET-29a(+) derivative carrying the *pnpAb*  pET-29a(+) derivative carrying the *pnpC1C2*  pET-29a(+) derivative carrying the *pnpC1C2b* | This study  This study  This study  This study  TaKaRa  Lab stock  Lab stock  Lab stock  Lab stock |
