# Supplementary material for: The Regulation of para-Nitrophenol Degradation in Pseudomonas putida DLL-E4
Source: PLoS One. 2016 May 18;11(5):e0155485. doi: 10.1371/journal.pone.0155485 (PMC4871426; doi:10.1371/journal.pone.0155485)
Supplement: S4 Table — The fold changes are reported in log2-based format. (DOCX) [file pone.0155485.s005.docx]

**Table S4. List of genes differentially expressed in *P. putida* DLL-E4 grown on glucose plus PNP compared to glucose. The fold changes are reported in log_2_-based format.**

| **Genes** | **Annotated functions** | **Fold changes**  **(log_2_)** | **Results** |
| --- | --- | --- | --- |
| DW66_0002 | transcriptional regulator DeoR | 1.36 | up |
| DW66_0006 | F0F1 ATP synthase subunit gamma | -1.64 | down |
| DW66_0007 | F0F1 ATP synthase subunit alpha | -1.02 | down |
| DW66_0008 | F0F1 ATP synthase subunit delta | -1.34 | down |
| DW66_0009 | F0F1 ATP synthase subunit B | -1.12 | down |
| DW66_0011 | F0F1 ATP synthase subunit A | -1.97 | down |
| DW66_0012 | F0F1 ATP synthase subunit I | -1.29 | down |
| DW66_0013 | partitioning protein ParB | -1.66 | down |
| DW66_0014 | cobyrinic acid ac-diamide synthase | -2.34 | down |
| DW66_0015 | 16S rRNA methyltransferase GidB | -1.34 | down |
| DW66_0017 | tRNA modification GTPase TrmE | 1.73 | up |
| DW66_0018 | inner membrane protein translocase component YidC | -2.62 | down |
| DW66_0019 | ribonuclease P | -4.72 | down |
| DW66_0020 | 50S ribosomal protein L34 | -4.16 | down |
| DW66_0024 | DNA gyrase subunit B | -1.38 | down |
| DW66_0025 | transmembrane protein | -1.02 | down |
| DW66_0027 | *beta*-lactamase domain-containing protein | 2.88 | up |
| DW66_0028 | FAD-dependent pyridine nucleotide-disulfide oxidoreductase | 1.86 | up |
| DW66_0029 | hypothetical protein | 1.03 | up |
| DW66_0034 | D,D-heptose 1,7-bisphosphate phosphatase | -1.21 | down |
| DW66_0035 | glycyl-tRNA synthetase subunit beta | -1.57 | down |
| DW66_0036 | glycyl-tRNA synthetase subunit alpha | -1.19 | down |
| DW66_0037 | DNA-3-methyladenine glycosylase I | -1.44 | down |
| DW66_0038 | lipid A biosynthesis lauroyl acyltransferase | -1.72 | down |
| DW66_0039 | Chaperone protein YscY (Yop proteins translocation protein Y) | 1.59 | up |
| DW66_0041 | sun protein | -1.77 | down |
| DW66_0042 | methionyl-tRNA formyltransferase | -1.61 | down |
| DW66_0044 | DNA protecting protein DprA | -1.46 | down |
| DW66_0045 | RimN | 1.11 | up |
| DW66_0046 | hypothetical protein | 1.48 | up |
| DW66_0048 | coproporphyrinogen III oxidase | -1.69 | down |
| DW66_0049 | shikimate 5-dehydrogenase | -1.54 | down |
| DW66_0052 | choline-sulfatase | -2.65 | down |
| DW66_0056 | tryptophan synthase subunit alpha | 1.27 | up |
| DW66_0060 | Dodecin Flavin-binding | 5.74 | up |
| DW66_0061 | hypothetical protein | 2.76 | up |
| DW66_0062 | luciferase-like monooxygenase | 2.69 | up |
| DW66_0064 | hypothetical protein | 1.18 | up |
| DW66_0065 | lipoprotein | 1.24 | up |
| DW66_0066 | lipoprotein | -1.31 | down |
| DW66_0067 | hypothetical protein | -1.92 | down |
| DW66_0069 | carbonic anhydrase | -1.27 | down |
| DW66_0071 | hypothetical protein | 1.24 | up |
| DW66_0072 | peptidase M3A and M3B, thimet/oligopeptidase F | -3.77 | down |
| DW66_0073 | radical SAM domain-containing protein | -1.22 | down |
| DW66_0075 | sulfate transporter | -4.19 | down |
| DW66_0077 | cytochrome c oxidase subunit II | 1.70 | up |
| DW66_0078 | cytochrome c oxidase subunit I | 3.78 | up |
| DW66_0079 | cytochrome C oxidase assembly protein | 3.82 | up |
| DW66_0080 | cytochrome c oxidase subunit III | 4.35 | up |
| DW66_0081 | Permeases | -1.24 | down |
| DW66_0082 | Cytochrome oxidase biogenesis protein Surf1, facilitates heme A insertion | 1.17 | up |
| DW66_0083 | transmembrane protein | 1.79 | up |
| DW66_0084 | cytochrome oxidase assembly | 1.29 | up |
| DW66_0088 | binding-protein-dependent transport system inner membrane protein | -1.85 | down |
| DW66_0089 | DL-methionine transporter, ATP-binding subunit | -2.21 | down |
| DW66_0091 | lipoprotein | -1.49 | down |
| DW66_0102 | DSBA oxidoreductase | -2.53 | down |
| DW66_0103 | endonuclease/exonuclease/phosphatase | -3.28 | down |
| DW66_0104 | diguanylate cyclase | -3.52 | down |
| DW66_0105 | N-acetylmuramyl-L-alanine amidase, negative regulator of AmpC, AmpD | -1.66 | down |
| DW66_0106 | EAL domain-containing protein | -2.36 | down |
| DW66_0109 | transport-associated protein | -1.55 | down |
| DW66_0110 | hypothetical protein | -1.80 | down |
| DW66_0111 | hypothetical protein | 1.69 | up |
| DW66_0112 | inhibitor of vertebrate lysozyme | -1.12 | down |
| DW66_0113 | glutamate/aspartate:proton symporter | -4.24 | down |
| DW66_0114 | nucleoside recognition domain-containing protein | -1.46 | down |
| DW66_0118 | ABC-type transport system involved in resistance to organic solvents, permease component USSDB6A | -1.16 | down |
| DW66_0123 | citrate/H+ symporter | -4.87 | down |
| DW66_0124 | hypothetical protein | -4.68 | down |
| DW66_0125 | aldehyde-activating protein | 1.41 | up |
| DW66_0126 | hypothetical protein | -1.12 | down |
| DW66_0128 | Holliday junction resolvasome, helicase subunit | -1.09 | down |
| DW66_0129 | Outer membrane receptor proteins, mostly Fe transport | 1.97 | up |
| DW66_0130 | hypothetical protein | 1.15 | up |
| DW66_0131 | succinate CoA transferase | 1.53 | up |
| DW66_0132 | hypothetical protein | -1.01 | down |
| DW66_0133 | NAD(P)(+) transhydrogenase | 1.81 | up |
| DW66_0134 | pyridine proton-translocating NAD(P) transhydrogenase | 2.10 | up |
| DW66_0135 | NAD(P) transhydrogenase subunit alpha | 1.49 | up |
| DW66_0136 | transcriptional regulator LysR | -2.37 | down |
| DW66_0137 | acyl-CoA dehydrogenase domain-containing protein | 3.03 | up |
| DW66_0138 | L-carnitine dehydratase/bile acid-inducible protein F | 3.11 | up |
| DW66_0139 | TonB-dependent siderophore receptor | -1.14 | down |
| DW66_0140 | anti-FecI sigma factor FecR | -2.23 | down |
| DW66_0141 | RNA polymerase sigma-24 factor | -2.55 | down |
| DW66_0144 | hypothetical protein | -1.52 | down |
| DW66_0145 | diguanylate cyclase/phosphodiesterase | -1.01 | down |
| DW66_0146 | type I secretion membrane fusion protein HlyD | 1.74 | up |
| DW66_0147 | type I secretion system ATPase | 1.29 | up |
| DW66_0148 | hypothetical protein | -3.44 | down |
| DW66_0150 | RTX toxin | 4.50 | up |
| DW66_0151 | T1SS secreted agglutinin RTX | 4.50 | up |
| DW66_0152 | RTX toxin | 4.40 | up |
| DW66_0153 | transporter | 4.35 | up |
| DW66_0155 | taurine dioxygenase | -5.99 | down |
| DW66_0156 | ABC transporter substrate-binding protein | -4.03 | down |
| DW66_0157 | ABC transporter ATP-binding protein | -2.05 | down |
| DW66_0158 | binding-protein-dependent transport system inner membrane protein | -1.06 | down |
| DW66_0159 | transcriptional regulator | -5.37 | down |
| DW66_0160 | Sel1 domain-containing protein | -1.75 | down |
| DW66_0161 | transcriptional regulator MarR | -7.28 | down |
| DW66_0162 | fusaric acid resistance protein region | -7.10 | down |
| DW66_0163 | membrane protein | -5.72 | down |
| DW66_0164 | secretion protein HlyD | -4.74 | down |
| DW66_0165 | RND efflux system outer membrane lipoprotein | -3.52 | down |
| DW66_0167 | hypothetical protein | -2.06 | down |
| DW66_0168 | hypothetical protein | -3.32 | down |
| DW66_0169 | cystathionine gamma-synthase | -3.22 | down |
| DW66_0170 | DNA-binding protein inhibitor Id-2-related protein | 5.12 | up |
| DW66_0171 | hypothetical protein | 1.03 | up |
| DW66_0175 | porphobilinogen deaminase | -1.11 | down |
| DW66_0176 | uroporphyrinogen-III synthase | -1.47 | down |
| DW66_0179 | disulfide bond formation protein DsbB | -5.29 | down |
| DW66_0181 | FKBP-type peptidylprolyl isomerase | -1.91 | down |
| DW66_0184 | ABC transporter ATP-binding protein | -2.62 | down |
| DW66_0185 | membrane protein | -4.20 | down |
| DW66_0187 | membrane protease | 2.92 | up |
| DW66_0188 | membrane-bound protease | 1.45 | up |
| DW66_0189 | YbaK/prolyl-tRNA synthetase associated domain-containing protein | 5.69 | up |
| DW66_0190 | hypothetical protein | 4.50 | up |
| DW66_0191 | NADH:ubiquinone oxidoreductase subunit 2 (chain N) | 2.81 | up |
| DW66_0192 | hypothetical protein | 3.76 | up |
| DW66_0193 | hypothetical protein | -2.26 | down |
| DW66_0194 | hypothetical protein | -3.47 | down |
| DW66_0196 | lipoprotein | -1.04 | down |
| DW66_0197 | hypothetical protein | -1.34 | down |
| DW66_0198 | ABC transporter permease | -1.47 | down |
| DW66_0200 | ppkA-like protein | 1.73 | up |
| DW66_0203 | SrfB | -1.12 | down |
| DW66_0204 | virulence factor | -2.71 | down |
| DW66_0205 | transcriptional regulator | -6.22 | down |
| DW66_0206 | oxidoreductase | -3.05 | down |
| DW66_0207 | 4Fe-4S ferredoxin | -2.59 | down |
| DW66_0208 | nitrate ABC transporter periplasmic nitrate-binding protein | -4.12 | down |
| DW66_0209 | nitrate ABC transporter permease | -4.69 | down |
| DW66_0210 | nitrate ABC transporter ATP-binding protein | -3.67 | down |
| DW66_0211 | phycobiliprotein | -1.11 | down |
| DW66_0212 | hypothetical protein | -2.29 | down |
| DW66_0213 | arylsulfotransferase | -2.39 | down |
| DW66_0216 | type III effector HopAC1 | -2.27 | down |
| DW66_0220 | GAF and PAS/PAC sensor-containing diguanylate cyclase/phosphodiesterase | -1.37 | down |
| DW66_0221 | binding-protein-dependent transport system inner membrane protein | -2.05 | down |
| DW66_0222 | ABC transporter ATP-binding protein | -2.75 | down |
| DW66_0223 | NLPA lipoprotein | -2.13 | down |
| DW66_0224 | DszA monooxygenase | -3.29 | down |
| DW66_0225 | acyl-CoA dehydrogenase type 2 | -7.36 | down |
| DW66_0226 | acyl-CoA dehydrogenase type 2 | -5.87 | down |
| DW66_0227 | hypothetical protein | -11.37 | down |
| DW66_0229 | polar amino acid ABC transporter inner membrane subunit | -2.07 | down |
| DW66_0230 | cystine transporter subunit | -1.30 | down |
| DW66_0231 | serine O-acetyltransferase | -1.37 | down |
| DW66_0232 | choline/carnitine/betaine transporter | -2.13 | down |
| DW66_0233 | taurine dioxygenase | -2.09 | down |
| DW66_0234 | binding-protein-dependent transport system inner membrane protein | -3.33 | down |
| DW66_0235 | taurine transporter, ATP-binding subunit | -4.18 | down |
| DW66_0236 | taurine ABC transporter periplasmic taurine-binding protein | -7.69 | down |
| DW66_0237 | outer membrane porin | -2.22 | down |
| DW66_0238 | peroxidase | -4.13 | down |
| DW66_0239 | NAD(P)H-dependent FMN reductase | -5.51 | down |
| DW66_0240 | aliphatic sulfonates ABC transporter substrate-binding protein | -5.21 | down |
| DW66_0241 | alkanesulfonate monooxygenase | -3.03 | down |
| DW66_0242 | ABC-type transporter membrane permease | -2.73 | down |
| DW66_0243 | aliphatic sulfonates transporter ATP-binding protein SsuB | -2.06 | down |
| DW66_0244 | TOBE domain-containing protein | -1.66 | down |
| DW66_0247 | thioesterase protein | -1.15 | down |
| DW66_0248 | RNA-binding S1 domain-containing protein | -2.50 | down |
| DW66_0251 | integral membrane sensor signal transduction histidine kinase | -1.88 | down |
| DW66_0252 | *alpha*-L-glutamate ligase | 1.34 | up |
| DW66_0257 | phosphoenolpyruvate carboxykinase | -1.95 | down |
| DW66_0259 | hypothetical protein | 1.73 | up |
| DW66_0260 | oxidoreductase, alpha (molybdopterin) subunit | 1.18 | up |
| DW66_0262 | LysM protein | 4.95 | up |
| DW66_0264 | ADP-ribose diphosphatase NudE | -1.08 | down |
| DW66_0265 | 3'(2'),5'-bisphosphate nucleotidase | -1.88 | down |
| DW66_0269 | hypothetical protein | -3.03 | down |
| DW66_0270 | dTDP-4-dehydrorhamnose 3,5-epimerase | 1.90 | up |
| DW66_0272 | TonB-dependent siderophore receptor | -4.62 | down |
| DW66_0273 | outer membrane porin | -2.15 | down |
| DW66_0274 | glutamate synthase | -1.66 | down |
| DW66_0275 | integral membrane sensor signal transduction histidine kinase | -3.35 | down |
| DW66_0276 | two component transcriptional regulator | -3.76 | down |
| DW66_0277 | TonB-dependent siderophore receptor | -1.77 | down |
| DW66_0278 | hypothetical protein | 1.45 | up |
| DW66_0279 | hypothetical protein | -3.37 | down |
| DW66_0280 | hypothetical protein | 1.81 | up |
| DW66_0282 | polar amino acid ABC transporter inner membrane subunit | -1.62 | down |
| DW66_0283 | polar amino acid ABC transporter inner membrane subunit | -2.39 | down |
| DW66_0286 | GABA permease | 2.50 | up |
| DW66_0289 | hypothetical protein | 1.36 | up |
| DW66_0290 | A/G-specific adenine glycosylase | 1.08 | up |
| DW66_0292 | transporter protein | -3.31 | down |
| DW66_0295 | imidazole glycerol phosphate synthase subunit HisH | -1.04 | down |
| DW66_0296 | hypothetical protein | -1.22 | down |
| DW66_0297 | 1-(5-phosphoribosyl)-5-[(5- phosphoribosylamino)methylideneamino] imidazole-4-carboxamide isomerase | -1.02 | down |
| DW66_0298 | Imidazole glycerol phosphate synthase subunit HisF | -2.05 | down |
| DW66_0299 | choline ABC transporter ATP-binding protein | 1.37 | up |
| DW66_0301 | glycine betaine/L-proline ABC transporter periplasmic binding protein | 1.57 | up |
| DW66_0302 | L-serine dehydratase | -1.39 | down |
| DW66_0303 | transcriptional regulator AraC | 4.58 | up |
| DW66_0304 | hypothetical protein | 1.12 | up |
| DW66_0306 | 3-hydroxybutyryl-CoA dehydrogenase | -2.85 | down |
| DW66_0307 | hypothetical protein | -4.42 | down |
| DW66_0308 | choline ABC transporter periplasmic binding protein | -2.58 | down |
| DW66_0309 | transcriptional regulator AraC | 1.91 | up |
| DW66_0310 | hypothetical protein | -14.47 | down |
| DW66_0311 | hypothetical protein | -4.03 | down |
| DW66_0312 | hypothetical protein | -2.72 | down |
| DW66_0313 | membrane dipeptidase | 1.21 | up |
| DW66_0314 | hypothetical protein | 1.89 | up |
| DW66_0315 | NADH:flavin oxidoreductase | 1.26 | up |
| DW66_0319 | Rieske (2Fe-2S) domain-containing protein | 1.22 | up |
| DW66_0321 | methyl-accepting chemotaxis sensory transducer | -2.05 | down |
| DW66_0322 | hypothetical protein | -1.87 | down |
| DW66_0323 | hypothetical protein | -2.61 | down |
| DW66_0324 | methyl-accepting chemotaxis sensory transducer | -3.96 | down |
| DW66_0326 | serine hydroxymethyltransferase | 1.17 | up |
| DW66_0333 | formaldehyde dehydrogenase | 2.99 | up |
| DW66_0334 | TnpA protein | -1.23 | down |
| DW66_0336 | amino acid permease-associated protein | -2.05 | down |
| DW66_0337 | acyltransferase | -3.36 | down |
| DW66_0339 | DNA-binding response regulator CreB | -3.54 | down |
| DW66_0340 | sensory histidine kinase CreC | -4.11 | down |
| DW66_0347 | hypothetical protein | -1.25 | down |
| DW66_0349 | lipopolysaccharide heptosyltransferase II | -1.60 | down |
| DW66_0350 | lipopolysaccharide heptosyltransferase I | -1.56 | down |
| DW66_0352 | lipopolysaccharide kinase | -1.05 | down |
| DW66_0353 | lipopolysaccharide kinase | -1.17 | down |
| DW66_0354 | lipopolysaccharide kinase | -1.70 | down |
| DW66_0356 | hypothetical protein | -2.84 | down |
| DW66_0357 | hypothetical protein | -4.73 | down |
| DW66_0358 | PepSY-associated TM helix domain-containing protein | -4.97 | down |
| DW66_0359 | TonB-dependent siderophore receptor | -2.45 | down |
| DW66_0362 | RNA polymerase sigma factor | -3.00 | down |
| DW66_0364 | signal-transduction protein containing cAMP-binding and CBS domains | -1.18 | down |
| DW66_0365 | chemotaxis protein CheY | 2.29 | up |
| DW66_0367 | ACT domain-containing protein | 1.23 | up |
| DW66_0368 | transporter | -1.46 | down |
| DW66_0372 | biotin synthase | 2.95 | up |
| DW66_0374 | carboxylesterase | 1.63 | up |
| DW66_0375 | biotin biosynthesis protein BioC | 2.10 | up |
| DW66_0376 | dithiobiotin synthetase | 1.65 | up |
| DW66_0377 | hypothetical protein | -1.27 | down |
| DW66_0378 | acyl-CoA dehydrogenase domain-containing protein | -1.15 | down |
| DW66_0379 | diguanylate cyclase | -1.61 | down |
| DW66_0383 | Stress induced hydrophobic peptide | 2.61 | up |
| DW66_0387 | pyrroloquinoline quinone biosynthesis protein PqqC | -1.34 | down |
| DW66_0389 | coenzyme PQQ biosynthesis protein PqqF | -1.25 | down |
| DW66_0392 | hypothetical protein | -1.80 | down |
| DW66_0393 | transcriptional regulator AsnC | -2.39 | down |
| DW66_0394 | 6-hydroxy-3-succinoylpyridine hydroxylase | -1.54 | down |
| DW66_0395 | PAS/PAC sensor-containing diguanylate cyclase/phosphodiesterase | 2.33 | up |
| DW66_0398 | 30S ribosomal protein S21 | -1.52 | down |
| DW66_0399 | DNA-binding/iron metalloprotein/AP endonuclease | -2.68 | down |
| DW66_0400 | glycerol-3-phosphate acyltransferase PlsY | -1.65 | down |
| DW66_0401 | dihydroneopterin aldolase | -2.53 | down |
| DW66_0402 | 2-amino-4-hydroxy-6- hydroxymethyldihydropteridine pyrophosphokinase | -3.24 | down |
| DW66_0404 | SpoVR protein | 5.39 | up |
| DW66_0405 | hypothetical protein | 5.38 | up |
| DW66_0406 | serine protein kinase PrkA | 5.41 | up |
| DW66_0407 | thiosulfate sulfurtransferase | 2.06 | up |
| DW66_0410 | dimethyladenosine transferase | 3.72 | up |
| DW66_0415 | mannose-1-phosphate guanylyltransferase | 1.86 | up |
| DW66_0416 | heat shock protein DnaJ domain-containing protein | 1.32 | up |
| DW66_0420 | spermidine/putrescine ABC transporter ATPase | -1.52 | down |
| DW66_0422 | binding-protein-dependent transport system inner membrane protein | -1.27 | down |
| DW66_0423 | binding-protein-dependent transport system inner membrane protein | 2.40 | up |
| DW66_0425 | phosphoglycolate phosphatase | -1.47 | down |
| DW66_0426 | anthranilate synthase component I | -1.31 | down |
| DW66_0428 | anthranilate synthase component II | -1.62 | down |
| DW66_0429 | anthranilate phosphoribosyltransferase | -1.55 | down |
| DW66_0431 | Lipoate-protein ligase A | -1.08 | down |
| DW66_0433 | protein OsmC | -2.26 | down |
| DW66_0437 | short-chain dehydrogenase/reductase SDR | -1.64 | down |
| DW66_0440 | N-acetyl-gamma-glutamyl-phosphate reductase | -1.75 | down |
| DW66_0444 | tyrosyl-tRNA synthetase | -2.02 | down |
| DW66_0445 | hypothetical protein | 5.11 | up |
| DW66_0446 | biotin-protein ligase | -1.42 | down |
| DW66_0447 | pantothenate kinase | -1.97 | down |
| DW66_0448 | hypothetical protein | -2.87 | down |
| DW66_0450 | Preprotein translocase subunit SecE | -1.97 | down |
| DW66_0451 | transcription antitermination protein NusG | -2.22 | down |
| DW66_0452 | 50S ribosomal protein L11 | -3.99 | down |
| DW66_0453 | 50S ribosomal protein L1 | -3.78 | down |
| DW66_0454 | 50S ribosomal protein L10 | -4.68 | down |
| DW66_0455 | 50S ribosomal protein L7/L12 | -2.18 | down |
| DW66_0456 | DNA-directed RNA polymerase subunit beta | -1.39 | down |
| DW66_0458 | 30S ribosomal protein S12 | -2.29 | down |
| DW66_0459 | protein RpsG | -2.28 | down |
| DW66_0460 | elongation factor G | -1.99 | down |
| DW66_0463 | 30S ribosomal protein S10 | -3.38 | down |
| DW66_0464 | ribosomal protein L3 | -4.12 | down |
| DW66_0465 | 50S ribosomal protein L4 | -3.79 | down |
| DW66_0466 | 50S ribosomal protein L23 | -3.48 | down |
| DW66_0467 | 50S ribosomal protein L2 | -3.44 | down |
| DW66_0468 | 30S ribosomal protein S19 | -3.15 | down |
| DW66_0469 | 50S ribosomal protein L22 | -3.49 | down |
| DW66_0470 | ribosomal protein S3 | -3.40 | down |
| DW66_0471 | 50S ribosomal protein L16 | -3.14 | down |
| DW66_0472 | 50S ribosomal protein L29 | -2.63 | down |
| DW66_0473 | 30S ribosomal protein S17 | -2.50 | down |
| DW66_0474 | 50S ribosomal protein L14 | -2.15 | down |
| DW66_0475 | 50S ribosomal protein L24 | -1.99 | down |
| DW66_0476 | 50S ribosomal protein L5 | -1.47 | down |
| DW66_0478 | 30S ribosomal protein S8 | -3.06 | down |
| DW66_0479 | 50S ribosomal protein L6 | -3.07 | down |
| DW66_0480 | 50S ribosomal protein L18 | -3.02 | down |
| DW66_0481 | 30S ribosomal protein S5 | -3.30 | down |
| DW66_0482 | 50S ribosomal protein L30 | -3.54 | down |
| DW66_0483 | 50S ribosomal protein L15 | -3.40 | down |
| DW66_0484 | preprotein translocase subunit SecY | -3.47 | down |
| DW66_0485 | Preprotein translocase subunit secY | -1.52 | down |
| DW66_0486 | 30S ribosomal protein S13 | -1.77 | down |
| DW66_0487 | 30S ribosomal protein S11 | -2.13 | down |
| DW66_0488 | 30S ribosomal protein S4 | -1.88 | down |
| DW66_0489 | DNA-directed RNA polymerase subunit alpha | -2.13 | down |
| DW66_0490 | 50S ribosomal protein L17 | -1.04 | down |
| DW66_0491 | catalase | 5.19 | up |
| DW66_0492 | bacterioferritin | 3.94 | up |
| DW66_0497 | membrane protein precursor | 1.35 | up |
| DW66_0498 | short-chain dehydrogenase | 1.70 | up |
| DW66_0500 | formate dehydrogenase subunit beta | 5.21 | up |
| DW66_0501 | formate dehydrogenase subunit gamma | 4.65 | up |
| DW66_0502 | formate dehydrogenase accessory protein FdhE | 3.26 | up |
| DW66_0504 | selenocysteine-specific translation elongation factor | 1.69 | up |
| DW66_0507 | hypothetical protein | -2.10 | down |
| DW66_0508 | transcriptional regulator Cro/CI | -1.23 | down |
| DW66_0509 | membrane protein YfcA | -3.23 | down |
| DW66_0510 | hypothetical protein | -11.80 | down |
| DW66_0511 | dTDP-4-dehydrorhamnose reductase | -1.32 | down |
| DW66_0513 | transcriptional regulator HxlR | -1.80 | down |
| DW66_0514 | transporter | -2.94 | down |
| DW66_0515 | outer membrane protein W | 2.53 | up |
| DW66_0516 | lipoprotein | -1.95 | down |
| DW66_0517 | efflux ABC transporter permease | -3.94 | down |
| DW66_0518 | efflux ABC transporter ATP-binding protein | -5.07 | down |
| DW66_0519 | zinc-binding protein | -4.75 | down |
| DW66_0520 | hypothetical protein | -1.11 | down |
| DW66_0521 | thioredoxin | 1.15 | up |
| DW66_0522 | type 12 methyltransferase | -1.08 | down |
| DW66_0524 | ATP cone domain protein | -3.23 | down |
| DW66_0525 | riboflavin biosynthesis protein RibD | -2.67 | down |
| DW66_0526 | riboflavin synthase subunit alpha | -2.35 | down |
| DW66_0527 | bifunctional 3,4-dihydroxy-2-butanone 4-phosphate synthase/GTP cyclohydrolase II-like protein | -1.44 | down |
| DW66_0530 | thiamine monophosphate kinase | -1.61 | down |
| DW66_0531 | phosphatidylglycerophosphatase A | -2.61 | down |
| DW66_0532 | hypothetical protein | -3.18 | down |
| DW66_0533 | GTP cyclohydrolase | -1.63 | down |
| DW66_0534 | Permeases | -1.99 | down |
| DW66_0535 | periplasmic binding protein | -4.01 | down |
| DW66_0536 | 1-deoxy-D-xylulose-5-phosphate synthase | -1.30 | down |
| DW66_0537 | polyprenyl synthetase | -3.36 | down |
| DW66_0538 | exodeoxyribonuclease VII small subunit | -2.95 | down |
| DW66_0539 | 3,4-dihydroxy-2-butanone 4-phosphate synthase | -1.96 | down |
| DW66_0541 | hypothetical protein | -3.02 | down |
| DW66_0545 | Multicopper oxidase | 1.88 | up |
| DW66_0547 | transcriptional regulator | -4.78 | down |
| DW66_0549 | Inner membrane protein | -1.48 | down |
| DW66_0551 | N-acetyltransferase GCN5 | -1.76 | down |
| DW66_0552 | ethanolamine ammonia-lyase, small subunit | 1.05 | up |
| DW66_0554 | ethanolamine transporter | -2.07 | down |
| DW66_0555 | aldehyde dehydrogenase | 1.30 | up |
| DW66_0556 | hypothetical protein | -12.37 | down |
| DW66_0558 | hypothetical protein | -2.27 | down |
| DW66_0559 | UDP-N-acetylmuramate:L-alanyl-gamma-D-glutamyl- meso-diaminopimelate ligase | -1.09 | down |
| DW66_0561 | lipoprotein | -2.02 | down |
| DW66_0562 | Oxidoreductase | 3.13 | up |
| DW66_0568 | branched-chain alpha-keto acid dehydrogenase subunit E2 | 1.15 | up |
| DW66_0569 | transketolase central region | 1.47 | up |
| DW66_0570 | TPP-dependent acetoin dehydrogenase subunit alpha | 2.01 | up |
| DW66_0577 | methyl-accepting chemotaxis sensory transducer | -4.85 | down |
| DW66_0580 | NUDIX hydrolase | -1.06 | down |
| DW66_0581 | translation initiation factor SUI1 | -4.18 | down |
| DW66_0584 | MATE efflux protein | -2.93 | down |
| DW66_0587 | penicillin-binding protein 1C | -2.52 | down |
| DW66_0588 | hypothetical protein | -3.25 | down |
| DW66_0591 | peptidase A24A prepilin type IV | 1.03 | up |
| DW66_0593 | lipoprotein | 1.78 | up |
| DW66_0595 | type 12 methyltransferase | -1.02 | down |
| DW66_0596 | hypothetical protein | -1.05 | down |
| DW66_0597 | dehydratase | 1.68 | up |
| DW66_0598 | 3-ketoacyl-ACP reductase | 1.95 | up |
| DW66_0602 | transcriptional regulator MerR | 2.86 | up |
| DW66_0603 | heavy metal translocating P-type ATPase | 2.15 | up |
| DW66_0605 | heavy metal transport/detoxification protein | 5.52 | up |
| DW66_0609 | short-chain dehydrogenase | 2.39 | up |
| DW66_0613 | beta alanine-pyruvate transaminase | 2.33 | up |
| DW66_0614 | methylmalonate-semialdehyde dehydrogenase | 3.14 | up |
| DW66_0618 | 30S ribosomal protein S20 | -2.79 | down |
| DW66_0619 | integral membrane protein MviN | -1.49 | down |
| DW66_0620 | hypothetical protein | -1.61 | down |
| DW66_0621 | bifunctional riboflavin kinase/FMN adenylyltransferase | -1.69 | down |
| DW66_0622 | isoleucyl-tRNA synthetase | -1.72 | down |
| DW66_0623 | lipoprotein signal peptidase | -1.55 | down |
| DW66_0624 | FKBP-type peptidylprolyl isomerase | -1.48 | down |
| DW66_0625 | 4-hydroxy-3-methylbut-2-enyl diphosphate reductase | -1.17 | down |
| DW66_0627 | hypothetical protein | -1.88 | down |
| DW66_0628 | Type IV fimbrial biogenesis protein PilW | -1.45 | down |
| DW66_0629 | Type IV fimbrial biogenesis protein PilX | -1.08 | down |
| DW66_0630 | type IV pili biogenesis protein PilE | -2.92 | down |
| DW66_0631 | FAD dependent oxidoreductase | -2.71 | down |
| DW66_0633 | competence lipoprotein ComL | -1.25 | down |
| DW66_0634 | pseudouridine synthase RluA | -1.59 | down |
| DW66_0635 | hypothetical protein | -2.43 | down |
| DW66_0636 | ATPase | 4.31 | up |
| DW66_0640 | hypothetical protein | -2.38 | down |
| DW66_0645 | type II secretion system protein | 2.47 | up |
| DW66_0647 | resolvase | 3.36 | up |
| DW66_0650 | integrase | -4.93 | down |
| DW66_0652 | ISPsy14, transposition helper protein | -13.72 | down |
| DW66_0653 | ISPsy14, transposase | -2.82 | down |
| DW66_0654 | hypothetical protein | -2.70 | down |
| DW66_0655 | hypothetical protein | -1.70 | down |
| DW66_0656 | transcriptional regulator | -3.93 | down |
| DW66_0657 | arsenical pump membrane protein | -2.46 | down |
| DW66_0658 | transporter protein | -4.01 | down |
| DW66_0659 | hypothetical protein | -3.23 | down |
| DW66_0660 | two component sigma54 specific transcriptional regulator | -1.87 | down |
| DW66_0661 | histidine kinase | -2.79 | down |
| DW66_0662 | malate dehydrogenase | 2.52 | up |
| DW66_0663 | polar amino acid ABC transporter inner membrane subunit | -4.76 | down |
| DW66_0664 | amino acid ABC transporter periplasmic amino acid-binding protein | -4.77 | down |
| DW66_0666 | cystathionine gamma-synthase | -3.63 | down |
| DW66_0667 | amino acid transporter | -2.93 | down |
| DW66_0669 | threonine synthase | -1.87 | down |
| DW66_0670 | transcriptional regulator AsnC | -2.25 | down |
| DW66_0672 | glyceraldehyde-3-phosphate dehydrogenase, NADP-dependent | -2.20 | down |
| DW66_0673 | endoribonuclease L-PSP | -2.00 | down |
| DW66_0677 | hypothetical protein | -2.17 | down |
| DW66_0678 | ABC transporter ATP-binding protein | -1.29 | down |
| DW66_0680 | elongation factor | -1.85 | down |
| DW66_0683 | hypothetical protein | 4.49 | up |
| DW66_0685 | hypothetical protein | 2.72 | up |
| DW66_0686 | inner membrane protein | -1.48 | down |
| DW66_0687 | hypothetical protein | 2.60 | up |
| DW66_0688 | FKBP-type peptidyl-prolyl cis-trans isomerase | -2.03 | down |
| DW66_0690 | alkylphosphonate utilization operon protein PhnA | 1.60 | up |
| DW66_0691 | octaprenyl diphosphate synthase | -2.11 | down |
| DW66_0692 | ribosomal protein L21 | -2.96 | down |
| DW66_0693 | 50S ribosomal protein L27 | -2.72 | down |
| DW66_0694 | GTPase ObgE | -3.87 | down |
| DW66_0695 | *gamma*-glutamyl kinase | -1.38 | down |
| DW66_0696 | CreA protein | -1.59 | down |
| DW66_0697 | Chromosome segregation ATPases | -1.96 | down |
| DW66_0698 | hypothetical protein | -3.48 | down |
| DW66_0699 | hypothetical protein | -2.41 | down |
| DW66_0702 | ribosomal-protein-alanine acetyltransferase | -1.62 | down |
| DW66_0704 | transcriptional regulator LysR | -1.73 | down |
| DW66_0705 | lysine exporter protein LysE/YggA | -5.69 | down |
| DW66_0706 | anti-FecI sigma factor FecR | -3.61 | down |
| DW66_0708 | hypothetical protein | -4.40 | down |
| DW66_0709 | transporter protein | -5.13 | down |
| DW66_0710 | DNA-3-methyladenine glycosylase II | -1.25 | down |
| DW66_0711 | methylated-DNA--protein-cysteine methyltransferase | -1.44 | down |
| DW66_0712 | mechanosensitive ion channel protein MscS | -2.42 | down |
| DW66_0714 | hypothetical protein | 1.97 | up |
| DW66_0715 | betaine-aldehyde dehydrogenase | -2.59 | down |
| DW66_0716 | NCS1 nucleoside transporter | -2.76 | down |
| DW66_0717 | Undecaprenyl pyrophosphate synthase | -2.61 | down |
| DW66_0718 | Mg2 transporter protein CorA | -3.12 | down |
| DW66_0719 | amino acid ABC transporter ATP-binding protein | -1.46 | down |
| DW66_0720 | amino acid ABC transporter permease | -1.18 | down |
| DW66_0721 | amino acid ABC transporter permease | -2.34 | down |
| DW66_0722 | amino acid ABC transporter periplasmic amino acid-binding protein | -1.00 | down |
| DW66_0723 | transcriptional regulator | -1.83 | down |
| DW66_0724 | EutB ectoine utilization protein | -2.64 | down |
| DW66_0725 | ectoine utilization protein EutC | -5.94 | down |
| DW66_0726 | EutD ectoine utilization protein | -2.49 | down |
| DW66_0727 | EutE ectoine utilization protein | -2.27 | down |
| DW66_0728 | succinate-semialdehyde dehydrogenase | -1.12 | down |
| DW66_0730 | isochorismatase hydrolase | 5.13 | up |
| DW66_0731 | hypothetical protein | 2.90 | up |
| DW66_0732 | potassium efflux system protein | 2.91 | up |
| DW66_0733 | permease | 4.12 | up |
| DW66_0734 | RND efflux system outer membrane lipoprotein | 3.04 | up |
| DW66_0735 | secretion protein HlyD | 3.41 | up |
| DW66_0736 | membrane protein | 1.24 | up |
| DW66_0737 | sulfate transporter | 2.33 | up |
| DW66_0738 | GTP-dependent nucleic acid-binding protein EngD | -2.45 | down |
| DW66_0739 | peptidyl-tRNA hydrolase | -1.81 | down |
| DW66_0741 | ribose-phosphate pyrophosphokinase | -2.36 | down |
| DW66_0742 | 4-diphosphocytidyl-2-C-methyl-D-erythritol kinase | -4.47 | down |
| DW66_0743 | outer membrane lipoprotein LolB | -1.42 | down |
| DW66_0745 | hypothetical protein | -1.74 | down |
| DW66_0746 | hypothetical protein | -1.31 | down |
| DW66_0747 | hypothetical protein | -1.52 | down |
| DW66_0748 | Argininosuccinate lyase | -2.11 | down |
| DW66_0749 | Phosphatidylserine synthase | -12.70 | down |
| DW66_0750 | hypothetical protein | -5.88 | down |
| DW66_0751 | glutamyl-tRNA reductase | -1.23 | down |
| DW66_0756 | Lipid A 3-O-deacylase | 1.55 | up |
| DW66_0757 | short-chain dehydrogenase | 1.74 | up |
| DW66_0758 | deoxyribodipyrimidine photo-lyase | 1.16 | up |
| DW66_0762 | NAD-dependent epimerase/dehydratase | 1.42 | up |
| DW66_0766 | hypoxanthine-guanine phosphoribosyltransferase | -2.44 | down |
| DW66_0767 | Tryptophan synthase beta chain like | -2.43 | down |
| DW66_0771 | hypothetical protein | -3.51 | down |
| DW66_0773 | hypothetical protein | -3.30 | down |
| DW66_0775 | Restriction endonuclease | 3.28 | up |
| DW66_0779 | transmembrane protein | -3.86 | down |
| DW66_0780 | rRNA (guanine-N(2)-)-methyltransferase | -2.76 | down |
| DW66_0781 | glycerate dehydrogenase | -1.03 | down |
| DW66_0783 | hypothetical protein | 1.20 | up |
| DW66_0784 | hypothetical protein | 2.35 | up |
| DW66_0788 | PemI-like protein | -1.99 | down |
| DW66_0789 | toxin ChpB | -3.06 | down |
| DW66_0790 | beta-lactamase domain-containing protein | -2.66 | down |
| DW66_0795 | glutathione peroxidase | 3.64 | up |
| DW66_0797 | methyl-accepting chemotaxis sensory transducer with Pas/Pac sensor | -1.68 | down |
| DW66_0798 | hypothetical protein | -3.67 | down |
| DW66_0799 | aspartyl-tRNA amidotransferase subunit B | -3.04 | down |
| DW66_0803 | Thymidine phosphorylase | 1.75 | up |
| DW66_0804 | hypothetical protein | -11.26 | down |
| DW66_0807 | TatD-related deoxyribonuclease | -1.91 | down |
| DW66_0808 | DNA-binding transcriptional regulator FruR | -2.10 | down |
| DW66_0809 | phosphoenolpyruvate-protein phosphotransferase | -2.05 | down |
| DW66_0810 | 1-phosphofructokinase | -1.46 | down |
| DW66_0811 | PTS system fructose | -1.18 | down |
| DW66_0812 | hypothetical protein | -3.70 | down |
| DW66_0814 | diguanylate cyclase | -1.98 | down |
| DW66_0815 | outer membrane porin | -1.21 | down |
| DW66_0820 | RND efflux transporter, MFP subunit | -1.38 | down |
| DW66_0821 | cell division protein Fic | -13.02 | down |
| DW66_0822 | hypothetical protein | -2.63 | down |
| DW66_0823 | hypothetical protein | -1.32 | down |
| DW66_0824 | response regulator receiver modulated CheW protein | -1.17 | down |
| DW66_0825 | anaerobic nitric oxide reductase transcriptional regulator | 2.22 | up |
| DW66_0826 | nitric oxide dioxygenase | 4.89 | up |
| DW66_0827 | disulfide bond formation protein B | -2.51 | down |
| DW66_0828 | ubiquinol oxidase subunit II | -1.30 | down |
| DW66_0829 | ubiquinol oxidase subunit 1 | -2.30 | down |
| DW66_0830 | cytochrome o ubiquinol oxidase subunit 3 | -1.98 | down |
| DW66_0831 | cytochrome o ubiquinol oxidase protein cyoD | -2.02 | down |
| DW66_0832 | protoheme IX farnesyltransferase | -1.03 | down |
| DW66_0834 | hypothetical protein | -4.31 | down |
| DW66_0835 | Ferredoxin | -1.73 | down |
| DW66_0837 | hypothetical protein | -3.51 | down |
| DW66_0839 | hypothetical protein | -3.27 | down |
| DW66_0841 | Restriction endonuclease | 3.53 | up |
| DW66_0842 | hypothetical protein | 3.56 | up |
| DW66_0846 | phosphonates import ATP-binding protein phnC | -1.92 | down |
| DW66_0847 | binding-protein-dependent transport system inner membrane protein | -2.01 | down |
| DW66_0848 | phosphonate ABC transporter inner membrane subunit | -1.92 | down |
| DW66_0852 | extracellular solute-binding protein | -2.38 | down |
| DW66_0853 | ATP-binding component of ABC transporter | -2.47 | down |
| DW66_0854 | amino acid ABC transporter permease | -3.41 | down |
| DW66_0855 | amino acid ABC transporter permease | -3.39 | down |
| DW66_0857 | extracellular solute-binding protein | -1.15 | down |
| DW66_0858 | endoribonuclease L-PSP | -2.67 | down |
| DW66_0859 | D-amino-acid dehydrogenase | -2.84 | down |
| DW66_0860 | transcriptional regulator LysR | -1.99 | down |
| DW66_0861 | S-adenosylmethionine--tRNA ribosyltransferase-isomerase | -2.76 | down |
| DW66_0862 | queuine tRNA-ribosyltransferase | -1.47 | down |
| DW66_0864 | preprotein translocase subunit SecD | -1.93 | down |
| DW66_0865 | preprotein translocase subunit SecF | -2.26 | down |
| DW66_0866 | exported protein | 1.27 | up |
| DW66_0867 | protein SuhB | -1.40 | down |
| DW66_0868 | RNA methyltransferase | -1.30 | down |
| DW66_0873 | iron-sulfur cluster assembly protein IscA | -1.10 | down |
| DW66_0874 | co-chaperone HscB | -2.11 | down |
| DW66_0875 | chaperone protein HscA | -2.64 | down |
| DW66_0876 | ferredoxin, 2Fe-2S type, ISC system | -1.93 | down |
| DW66_0878 | nucleoside diphosphate kinase | -1.28 | down |
| DW66_0879 | radical SAM protein | -1.42 | down |
| DW66_0880 | type IV pilus biogenesis/stability protein PilW | -1.91 | down |
| DW66_0881 | transcriptional regulator XRE | -2.11 | down |
| DW66_0882 | 4-hydroxy-3-methylbut-2-en-1-yl diphosphate synthase | -1.87 | down |
| DW66_0883 | histidyl-tRNA synthetase | -2.07 | down |
| DW66_0884 | hypothetical protein | -1.74 | down |
| DW66_0885 | dehydrogenase | -1.75 | down |
| DW66_0889 | flavodoxin/nitric oxide synthase | -2.91 | down |
| DW66_0891 | TonB-dependent siderophore receptor | -2.68 | down |
| DW66_0892 | hydroxylase | -3.28 | down |
| DW66_0893 | Sel1 domain-containing protein | -3.41 | down |
| DW66_0894 | ornithine decarboxylase | -2.47 | down |
| DW66_0895 | RNA polymerase sigma-25 factor | -3.97 | down |
| DW66_0896 | anti-FecI sigma factor FecR | -4.47 | down |
| DW66_0897 | TonB-dependent siderophore receptor | -4.58 | down |
| DW66_0898 | glycine betaine/L-proline ABC transporter ATPase | -2.83 | down |
| DW66_0899 | binding-protein-dependent transport system inner membrane protein | -6.31 | down |
| DW66_0900 | glycine betaine ABC transporter substrate-binding protein | -5.42 | down |
| DW66_0901 | glycine/betaine ABC transporter permease | -4.24 | down |
| DW66_0902 | peptide chain release factor 3 | -3.31 | down |
| DW66_0903 | extracellular solute-binding protein | -1.32 | down |
| DW66_0905 | FAD dependent oxidoreductase | -2.69 | down |
| DW66_0906 | transcriptional regulator AraC | -3.16 | down |
| DW66_0907 | dipeptide transporter, ATP-binding subunit | 2.05 | up |
| DW66_0911 | peptide ABC transporter substrate-binding protein | 1.72 | up |
| DW66_0912 | outer membrane porin | -3.69 | down |
| DW66_0913 | extracellular solute-binding protein | -3.52 | down |
| DW66_0914 | extracellular solute-binding protein | -2.01 | down |
| DW66_0917 | transcriptional regulator | 2.76 | up |
| DW66_0918 | auxin efflux carrier | -2.23 | down |
| DW66_0921 | conjugal transfer protein TraR | -1.89 | down |
| DW66_0922 | rare lipoprotein A | -4.05 | down |
| DW66_0926 | rod shape-determining protein MreB | -2.12 | down |
| DW66_0927 | rod shape-determining protein MreC | -3.02 | down |
| DW66_0928 | rod shape-determining protein MreD | -4.30 | down |
| DW66_0929 | Maf-like protein | -2.26 | down |
| DW66_0931 | hypothetical protein | -1.02 | down |
| DW66_0932 | nitrilase/cyanide hydratase and apolipoprotein N-acyltransferase | 1.65 | up |
| DW66_0933 | peptidase U62 modulator of DNA gyrase | 2.03 | up |
| DW66_0936 | FagA protein | -3.50 | down |
| DW66_0937 | fumarate hydratase | -3.29 | down |
| DW66_0938 | superoxide dismutase | -2.99 | down |
| DW66_0939 | phosphotransferase system, phosphocarrier protein HPr | -1.15 | down |
| DW66_0942 | sigma 54 modulation protein/ribosomal protein S30EA | 2.16 | up |
| DW66_0948 | KpsF/GutQ protein | -1.51 | down |
| DW66_0949 | hypothetical protein | -4.09 | down |
| DW66_0950 | ABC transporter-like protein | -3.27 | down |
| DW66_0951 | ABC transporter, permease component YrbE | -3.66 | down |
| DW66_0952 | toluene tolerance protein Ttg2C | -1.54 | down |
| DW66_0955 | BolA protein | -1.30 | down |
| DW66_0956 | UDP-N-acetylglucosamine 1-carboxyvinyltransferase | -1.59 | down |
| DW66_0957 | ATP phosphoribosyltransferase, catalytic subunit | -3.04 | down |
| DW66_0958 | histidinol dehydrogenase | -1.67 | down |
| DW66_0959 | histidinol-phosphate aminotransferase | -1.18 | down |
| DW66_0962 | Ketosteroid isomerase-related protein | -1.23 | down |
| DW66_0966 | 23S rRNA mA1618 methyltransferase | 1.72 | up |
| DW66_0967 | valyl-tRNA synthetase | -1.21 | down |
| DW66_0969 | DNA polymerase III subunit chi | -2.76 | down |
| DW66_0971 | YjgP permease | -1.17 | down |
| DW66_0973 | transposase | -1.69 | down |
| DW66_0974 | RDD domain-containing protein | -1.18 | down |
| DW66_0976 | glycine cleavage system T protein | 1.88 | up |
| DW66_0979 | glycine cleavage system protein H | -1.91 | down |
| DW66_0981 | hypothetical protein | 2.43 | up |
| DW66_0982 | histidine kinase | -1.15 | down |
| DW66_0984 | sodium/glutamate symport carrier protein GltS | -3.58 | down |
| DW66_0985 | sigma-54 dependent transcriptional regulator/sensory box protein | -1.64 | down |
| DW66_0986 | Acetyl-CoA carboxylase alpha subunit | 2.89 | up |
| DW66_0987 | carbamate kinase | 4.35 | up |
| DW66_0988 | ornithine carbamoyltransferase | 6.34 | up |
| DW66_0989 | arginine deiminase | 7.24 | up |
| DW66_0990 | arginine/ornithine antiporter | 4.27 | up |
| DW66_0992 | hypothetical protein | -1.51 | down |
| DW66_0995 | anti-FecI sigma factor FecR | -2.62 | down |
| DW66_0996 | RNA polymerase sigma-26 factor | -5.67 | down |
| DW66_0998 | phosphogluconate dehydratase | -1.76 | down |
| DW66_1000 | two component transcriptional regulator | -1.60 | down |
| DW66_1001 | integral membrane sensor signal transduction histidine kinase | -2.11 | down |
| DW66_1004 | binding-protein-dependent transport system inner membrane protein | -2.87 | down |
| DW66_1005 | binding-protein-dependent transport system inner membrane protein | -2.76 | down |
| DW66_1006 | sugar ABC transporter ATP-binding protein | -1.76 | down |
| DW66_1008 | aldose 1-epimerase | -1.92 | down |
| DW66_1009 | DNA-binding transcriptional regulator HexR | -1.48 | down |
| DW66_1010 | glucose-6-phosphate 1-dehydrogenase | -2.21 | down |
| DW66_1013 | 2-isopropylmalate synthase | 1.58 | up |
| DW66_1014 | peptidase M23B | -1.77 | down |
| DW66_1015 | exodeoxyribonuclease VII large subunit | -2.51 | down |
| DW66_1016 | transcriptional regulator LysR | -1.06 | down |
| DW66_1019 | inosine 5'-monophosphate dehydrogenase | -1.38 | down |
| DW66_1020 | GMP synthase | -1.04 | down |
| DW66_1022 | hypothetical protein | -2.57 | down |
| DW66_1023 | ADP-ribosyl-(dinitrogen reductase) hydrolase | -2.41 | down |
| DW66_1024 | hypothetical protein | -2.40 | down |
| DW66_1025 | multicopper oxidase type 3 | -2.16 | down |
| DW66_1026 | CRISPR-associated protein Csn1 | -2.46 | down |
| DW66_1028 | hypothetical protein | -2.34 | down |
| DW66_1030 | hypothetical protein | -1.51 | down |
| DW66_1031 | sec-independent protein translocase, TatC subunit | -2.51 | down |
| DW66_1032 | twin-arginine translocation protein subunit TatB | -6.47 | down |
| DW66_1033 | twin arginine-targeting protein translocase | -2.09 | down |
| DW66_1035 | phosphatase | -2.11 | down |
| DW66_1036 | lipoprotein UxpA | -3.05 | down |
| DW66_1037 | type II secretion protein C | -7.46 | down |
| DW66_1038 | general secretion pathway protein D | -5.78 | down |
| DW66_1039 | type II secretion system protein E | -2.58 | down |
| DW66_1040 | general secretion pathway protein F | -2.71 | down |
| DW66_1041 | general secretion pathway protein G | -3.56 | down |
| DW66_1042 | general secretion pathway protein H | -3.86 | down |
| DW66_1043 | type II secretion system protein I/J | -5.10 | down |
| DW66_1044 | type II secretion system protein J | -3.67 | down |
| DW66_1045 | general secretion pathway protein L | -2.82 | down |
| DW66_1046 | type II secretion system protein M | -3.90 | down |
| DW66_1047 | type II secretion pathway protein GspN | -4.55 | down |
| DW66_1049 | filamentous hemagglutinin outer membrane protein | -2.23 | down |
| DW66_1050 | secretion protein | -4.85 | down |
| DW66_1054 | transferase | -1.69 | down |
| DW66_1056 | preQ0 transporter | -3.37 | down |
| DW66_1057 | phosphoribosylglycinamide formyltransferase 2 | -1.95 | down |
| DW66_1061 | signal recognition particle protein | -1.59 | down |
| DW66_1062 | 30S ribosomal protein S16 | -2.59 | down |
| DW66_1063 | 16S rRNA-processing protein RimM | -3.67 | down |
| DW66_1064 | tRNA (guanine-N1)-methyltransferase | -3.62 | down |
| DW66_1065 | 50S ribosomal protein L19 | -2.43 | down |
| DW66_1066 | thioesterase protein | -4.41 | down |
| DW66_1067 | site-specific tyrosine recombinase XerD | -3.04 | down |
| DW66_1069 | homoserine dehydrogenase | -1.62 | down |
| DW66_1071 | response regulator receiver protein | -5.73 | down |
| DW66_1072 | hypothetical protein | -1.01 | down |
| DW66_1073 | NAD(FAD)-utilizing enzyme possibly involved in translation | -2.11 | down |
| DW66_1075 | YaeQ protein | -2.26 | down |
| DW66_1076 | single-stranded-DNA-specific exonuclease RecJ | -2.70 | down |
| DW66_1077 | hypothetical protein | -2.09 | down |
| DW66_1078 | NADH:flavin oxidoreductase | 4.10 | up |
| DW66_1079 | HicB protein | -1.54 | down |
| DW66_1080 | *gamma*-aminobutyraldehyde dehydrogenase | 3.22 | up |
| DW66_1082 | binding-protein-dependent transport system inner membrane protein | 1.13 | up |
| DW66_1083 | spermidine/putrescine ABC transporter ATPase | 1.37 | up |
| DW66_1084 | extracellular solute-binding protein | 2.81 | up |
| DW66_1089 | CheW protein | 1.25 | up |
| DW66_1090 | CheA signal transduction histidine kinase | 1.75 | up |
| DW66_1091 | chemotaxis-specific methylesterase | 2.30 | up |
| DW66_1092 | response regulator receiver modulated diguanylate cyclase | 1.88 | up |
| DW66_1093 | peptide chain release factor 2 | -1.44 | down |
| DW66_1094 | lysyl-tRNA synthetase | -1.66 | down |
| DW66_1098 | lipoprotein | -1.21 | down |
| DW66_1099 | hypothetical protein | -1.89 | down |
| DW66_1102 | hypothetical protein | 1.63 | up |
| DW66_1105 | peptidase M22 glycoprotease | -1.87 | down |
| DW66_1107 | hypothetical protein | -1.42 | down |
| DW66_1109 | extensin protein | -1.84 | down |
| DW66_1112 | hypothetical protein | -1.10 | down |
| DW66_1113 | transcriptional regulator TetR | -1.81 | down |
| DW66_1114 | RND efflux transporter, MFP subunit | -2.48 | down |
| DW66_1115 | acriflavin resistance protein | -2.91 | down |
| DW66_1118 | hypothetical protein | -3.32 | down |
| DW66_1120 | glycerol-3-phosphate acyltransferase | -1.30 | down |
| DW66_1124 | rRNA (guanine-N(1)-)-methyltransferase | -2.47 | down |
| DW66_1125 | succinyl-diaminopimelate desuccinylase | -1.99 | down |
| DW66_1127 | UBA/THIF-type NAD/FAD binding protein | -2.45 | down |
| DW66_1130 | 2,3,4,5-tetrahydropyridine-2,6-carboxylate N-succinyltransferase | -1.51 | down |
| DW66_1131 | oxidoreductase, FAD-binding protein | -1.00 | down |
| DW66_1132 | Na+/H+ antiporter | -1.30 | down |
| DW66_1136 | 30S ribosomal protein S2 | -1.10 | down |
| DW66_1137 | elongation factor Ts | -2.52 | down |
| DW66_1138 | uridylate kinase | -1.08 | down |
| DW66_1139 | ribosome recycling factor | -1.25 | down |
| DW66_1140 | undecaprenyl diphosphate synthase | -3.03 | down |
| DW66_1141 | phosphatidate cytidylyltransferase | -2.13 | down |
| DW66_1144 | surface antigen | -1.86 | down |
| DW66_1145 | outer membrane chaperone Skp | -1.63 | down |
| DW66_1146 | UDP-3-O-(3-hydroxymyristoyl) glucosamine N-acyltransferase | -1.72 | down |
| DW66_1148 | UDP-N-acetylglucosamine acyltransferase | -1.25 | down |
| DW66_1149 | lipid-A-disaccharide synthase | -1.87 | down |
| DW66_1150 | ribonuclease HII | -4.10 | down |
| DW66_1151 | hypothetical protein | -1.23 | down |
| DW66_1152 | DNA polymerase III subunit alpha | -1.48 | down |
| DW66_1154 | tRNA(Ile)-lysidine synthetase | -1.10 | down |
| DW66_1155 | CTP synthetase | -1.27 | down |
| DW66_1156 | 2-dehydro-3-deoxyphosphooctonate aldolase | -1.79 | down |
| DW66_1157 | phosphopyruvate hydratase | -1.46 | down |
| DW66_1161 | S-(hydroxymethyl)glutathione dehydrogenase/class III alcohol dehydrogenase | 3.67 | up |
| DW66_1162 | S-formylglutathione hydrolase | 3.97 | up |
| DW66_1164 | tRNA pseudouridine synthase D | -1.47 | down |
| DW66_1165 | stationary phase survival protein SurE | -1.38 | down |
| DW66_1168 | RNA polymerase sigma factor RpoS | 2.04 | up |
| DW66_1169 | 4Fe-4S ferredoxin | -2.16 | down |
| DW66_1170 | DNA mismatch repair protein MutS | -1.55 | down |
| DW66_1173 | recA protein | 1.35 | up |
| DW66_1177 | transcriptional regulator AsnC | 1.26 | up |
| DW66_1179 | hypothetical protein | -2.39 | down |
| DW66_1181 | amino acid ABC transporter permease | -1.73 | down |
| DW66_1184 | hypothetical protein | -5.06 | down |
| DW66_1186 | amino acid MFS transporter | -3.22 | down |
| DW66_1187 | ISPsy14, transposase | -2.79 | down |
| DW66_1188 | ATPase AAA | -3.00 | down |
| DW66_1189 | D-amino acid dehydrogenase small subunit | 2.27 | up |
| DW66_1190 | Endoribonuclease L-PSP | 3.43 | up |
| DW66_1191 | hypothetical protein | 1.86 | up |
| DW66_1192 | N-acetyltransferase | -1.21 | down |
| DW66_1194 | transcriptional regulator | 3.10 | up |
| DW66_1195 | hypothetical protein | -1.16 | down |
| DW66_1196 | hypothetical protein | -7.39 | down |
| DW66_1197 | hypothetical protein | -2.40 | down |
| DW66_1198 | transcriptional regulator | -1.73 | down |
| DW66_1201 | hypothetical protein | 4.08 | up |
| DW66_1202 | hypothetical protein | 4.68 | up |
| DW66_1203 | hypothetical protein | 2.40 | up |
| DW66_1205 | recombination regulator RecX | -2.72 | down |
| DW66_1206 | Decarboxylase | 2.46 | up |
| DW66_1207 | hypothetical protein | 1.06 | up |
| DW66_1208 | secreted protein | -1.63 | down |
| DW66_1209 | DTW domain-containing protein | -3.06 | down |
| DW66_1213 | oxidoreductase, FAD/NAD(P)-binding subunit | 1.85 | up |
| DW66_1215 | membrane protein | 7.60 | up |
| DW66_1221 | alkyl hydroperoxide reductase/ thiol specific antioxidant/ Mal allergen | 1.60 | up |
| DW66_1222 | Heat shock protein | -2.02 | down |
| DW66_1223 | hypothetical protein | -1.22 | down |
| DW66_1226 | two component transcriptional regulator | -3.87 | down |
| DW66_1227 | integral membrane sensor signal transduction histidine kinase | -3.42 | down |
| DW66_1228 | cysteine synthase B | -1.66 | down |
| DW66_1229 | 23S rRNA 5-methyluridine methyltransferase | -1.13 | down |
| DW66_1233 | hypothetical protein | 1.43 | up |
| DW66_1235 | phosphoribosylglycinamide formyltransferase | -1.88 | down |
| DW66_1236 | phosphoribosylaminoimidazole synthetase | -2.39 | down |
| DW66_1238 | hypothetical protein | -1.05 | down |
| DW66_1239 | DNA replication initiation factor | -3.18 | down |
| DW66_1240 | NLP/P60 protein | -3.24 | down |
| DW66_1243 | hypothetical protein | -1.21 | down |
| DW66_1244 | hypothetical protein | 1.73 | up |
| DW66_1245 | cob(I)yrinic acid a,c-diamide adenosyltransferase | -3.35 | down |
| DW66_1246 | cobyrinic acid a,c-diamide synthase | -3.65 | down |
| DW66_1247 | cob(II)yrinic acid a,c-diamide reductase | -2.99 | down |
| DW66_1248 | cobalamin biosynthesis protein | -1.57 | down |
| DW66_1249 | threonine-phosphate decarboxylase | -2.34 | down |
| DW66_1250 | cobyric acid synthase | -2.87 | down |
| DW66_1251 | adenosylcobinamide kinase/adenosylcobinamide-phosphate guanylyltransferase | -3.03 | down |
| DW66_1252 | nicotinate-nucleotide-dimethylbenzimidazole phosphoribosyltransferase | -1.81 | down |
| DW66_1253 | alpha-ribazole phosphatase | -2.24 | down |
| DW66_1254 | cobalamin synthase | -3.10 | down |
| DW66_1255 | glycoside hydrolase | -3.76 | down |
| DW66_1262 | transcriptional regulator LysR | -3.42 | down |
| DW66_1263 | aromatic hydrocarbon degradation membrane protein | -1.95 | down |
| DW66_1265 | hypothetical protein | -2.54 | down |
| DW66_1266 | Sel1 domain-containing protein | -4.18 | down |
| DW66_1267 | DNA recombination protein RmuC | -1.05 | down |
| DW66_1269 | hypothetical protein | -1.97 | down |
| DW66_1271 | hypothetical protein | -2.96 | down |
| DW66_1273 | multidrug efflux pump | -1.44 | down |
| DW66_1275 | recombination associated protein | -2.38 | down |
| DW66_1276 | type II secretion system protein J | -3.01 | down |
| DW66_1277 | methionine aminopeptidase | -1.18 | down |
| DW66_1278 | hypothetical protein | -9.66 | down |
| DW66_1279 | molybdopterin oxidoreductase | -2.26 | down |
| DW66_1280 | nitrite reductase, (NAD(P)H) large subunit | -1.33 | down |
| DW66_1282 | transcriptional regulator LysR | -1.13 | down |
| DW66_1283 | FKBP-type peptidylprolyl isomerase | -2.44 | down |
| DW66_1284 | DUF1232 domain-containing protein | -2.66 | down |
| DW66_1285 | transcriptional regulator Cro/CI | 1.13 | up |
| DW66_1288 | carboxyl-terminal protease | 2.62 | up |
| DW66_1289 | hypothetical protein | -1.24 | down |
| DW66_1291 | hydrolase | 1.91 | up |
| DW66_1292 | ABC transporter ATP-binding protein | 1.29 | up |
| DW66_1296 | ABC transporter substrate-binding protein | 1.36 | up |
| DW66_1299 | hypothetical protein | 1.03 | up |
| DW66_1301 | ribosomal large subunit pseudouridine synthase A | -2.97 | down |
| DW66_1302 | cell division topological specificity factor MinE | -1.47 | down |
| DW66_1303 | septum site-determining protein MinD | -1.64 | down |
| DW66_1304 | septum formation inhibitor | -3.34 | down |
| DW66_1305 | lipid A biosynthesis lauroyl acyltransferase | -2.20 | down |
| DW66_1306 | patatin | -3.05 | down |
| DW66_1671 | glycine betaine ABC transporter substrate-binding protein | 3.51 | up |
| DW66_1674 | hypothetical protein | 1.28 | up |
| DW66_1677 | hypothetical protein | -2.84 | down |
| DW66_1680 | asparagine synthase amidotransferase | -1.96 | down |
| DW66_1681 | 5-methylaminomethyl-2-thiouridine methyltransferase | -1.45 | down |
| DW66_1683 | hypothetical protein | 1.36 | up |
| DW66_1686 | hypothetical protein | -1.62 | down |
| DW66_1687 | BolA protein | -1.53 | down |
| DW66_1690 | ABC transporter ATP-binding protein | 1.28 | up |
| DW66_1691 | GAF and PAS/PAC sensor-containing diguanylate cyclase/phosphodiesterase | 2.36 | up |
| DW66_1692 | Pyrroloquinoline quinone (Coenzyme PQQ) biosynthesis protein C | 4.15 | up |
| DW66_1696 | methylthioribose-1-phosphate isomerase | -2.76 | down |
| DW66_1697 | DNA gyrase subunit A | -1.77 | down |
| DW66_1698 | phosphoserine aminotransferase | -2.12 | down |
| DW66_1699 | chorismate mutase | -2.21 | down |
| DW66_1700 | 3-phosphoshikimate 1-carboxyvinyltransferase | -1.87 | down |
| DW66_1702 | 30S ribosomal protein S1 | -1.89 | down |
| DW66_1704 | integration host factor subunit beta | 2.85 | up |
| DW66_1707 | beta-lactamase | -3.68 | down |
| DW66_1710 | ISPsy14, transposase | -2.79 | down |
| DW66_1711 | ATPase AAA | -2.99 | down |
| DW66_1712 | hypothetical protein | -2.44 | down |
| DW66_1713 | dTDP-4-dehydrorhamnose 3,5-epimerase | -1.01 | down |
| DW66_1718 | transposase IS66 | -2.25 | down |
| DW66_1719 | Mobile element protein | -2.08 | down |
| DW66_1722 | GDP-D-mannose dehydratase | -1.28 | down |
| DW66_1723 | glycosyl transferase group 1 protein | -2.78 | down |
| DW66_1724 | glycosyl transferase group 1 protein | -3.63 | down |
| DW66_1725 | NAD-dependent dehydratase | -2.86 | down |
| DW66_1728 | Mobile element protein | -2.18 | down |
| DW66_1729 | transposase IS66 | -2.25 | down |
| DW66_1731 | ATPase AAA | -3.00 | down |
| DW66_1732 | ISPsy14, transposase | -2.79 | down |
| DW66_1736 | Helix-hairpin-helix repeat-containing competence protein ComEA | 1.49 | up |
| DW66_1737 | hypothetical protein | 4.43 | up |
| DW66_1738 | orotidine 5'-phosphate decarboxylase | -2.56 | down |
| DW66_1739 | undecaprenyl pyrophosphate phosphatase | -3.88 | down |
| DW66_1740 | hypothetical protein | -1.28 | down |
| DW66_1744 | MFS transporter | -3.32 | down |
| DW66_1746 | methyl-accepting chemotaxis sensory transducer | 2.82 | up |
| DW66_1747 | membrane protein | -1.07 | down |
| DW66_1749 | glutaredoxin | -3.09 | down |
| DW66_1750 | GTP cyclohydrolase I | -1.42 | down |
| DW66_1751 | Smr protein/MutS2 | -2.55 | down |
| DW66_1752 | hypothetical protein | -1.04 | down |
| DW66_1753 | isochorismatase hydrolase | 1.29 | up |
| DW66_1754 | N5-glutamine S-adenosyl-L-methionine-dependent methyltransferase | -1.18 | down |
| DW66_1755 | hypothetical protein | -1.88 | down |
| DW66_1756 | alpha/beta hydrolase fold protein | -2.00 | down |
| DW66_1758 | transporter protein | -1.33 | down |
| DW66_1760 | hypothetical protein | -3.17 | down |
| DW66_1761 | hypothetical protein | -2.41 | down |
| DW66_1763 | hypothetical protein | -2.60 | down |
| DW66_1765 | hypothetical protein | 1.92 | up |
| DW66_1766 | sulfatase | -3.08 | down |
| DW66_1768 | DNA and RNA helicases | 1.74 | up |
| DW66_1772 | 1-acyl-sn-glycerol-3-phosphate acyltransferase | -2.97 | down |
| DW66_1773 | enoyl-CoA hydratase | -1.66 | down |
| DW66_1774 | TonB-dependent siderophore receptor | -1.67 | down |
| DW66_1775 | hypothetical protein | 2.42 | up |
| DW66_1776 | transporter protein | -1.57 | down |
| DW66_1777 | 3-ketoacyl-(acyl-carrier-protein) reductase | 2.32 | up |
| DW66_1782 | elongation factor P | -2.00 | down |
| DW66_1783 | protein OsmC | 4.40 | up |
| DW66_1784 | transcriptional regulator MarR | -1.48 | down |
| DW66_1785 | transcriptional regulator LysR | -1.76 | down |
| DW66_1786 | membrane protein | -2.03 | down |
| DW66_1787 | transcriptional regulator LysR | 2.39 | up |
| DW66_1791 | Spermidine synthase-like protein | -2.52 | down |
| DW66_1792 | DEAD/DEAH box helicase | -5.04 | down |
| DW66_1793 | extradiol ring-cleavage dioxygenase class III protein subunit B | 2.38 | up |
| DW66_1795 | heat shock protein HtpX | 1.14 | up |
| DW66_1800 | ATP/GTP-binding protein | -3.22 | down |
| DW66_1801 | GAF domain-containing protein | -1.42 | down |
| DW66_1802 | hypothetical protein | 1.67 | up |
| DW66_1803 | fimbrial protein | -4.45 | down |
| DW66_1807 | Outer membrane autotransporter barrel | -3.65 | down |
| DW66_1809 | pili assembly chaperone | -4.44 | down |
| DW66_1810 | type I fimbrial protein FimA | -1.57 | down |
| DW66_1812 | acyl-CoA dehydrogenase | 2.79 | up |
| DW66_1813 | glutathione S-transferase domain-containing protein | -1.97 | down |
| DW66_1814 | ABC transporter ATP-binding protein | -2.23 | down |
| DW66_1815 | membrane protein | -1.83 | down |
| DW66_1816 | acetyltransferase | -1.27 | down |
| DW66_1817 | MotA/TolQ/ExbB proton channel | -2.07 | down |
| DW66_1818 | biopolymer transport protein ExbD/TolR | -2.07 | down |
| DW66_1819 | tetraacyldisaccharide 4'-kinase | -2.46 | down |
| DW66_1820 | hypothetical protein | -1.06 | down |
| DW66_1824 | ribonuclease E | -2.90 | down |
| DW66_1825 | hypothetical protein | -2.02 | down |
| DW66_1826 | pseudouridine synthase RluA | -2.88 | down |
| DW66_1827 | hydrolase | -2.73 | down |
| DW66_1828 | signal peptide peptidase SppA, 36K type | -2.17 | down |
| DW66_1830 | hypothetical protein | -2.91 | down |
| DW66_1831 | 50S ribosomal protein L32 | -3.10 | down |
| DW66_1832 | phosphate acyltransferase | -4.94 | down |
| DW66_1833 | malonyl CoA-acyl carrier protein transacylase | -1.01 | down |
| DW66_1835 | acyl carrier protein | 1.13 | up |
| DW66_1836 | 3-oxoacyl-(acyl carrier protein) synthase II | -1.79 | down |
| DW66_1837 | 4-amino-4-deoxychorismate lyase | -1.91 | down |
| DW66_1838 | aminodeoxychorismate lyase | -1.73 | down |
| DW66_1840 | DNA polymerase III subunit delta | -1.24 | down |
| DW66_1841 | deoxyribonuclease TatD | -1.03 | down |
| DW66_1842 | transcriptional regulator TetR | -1.20 | down |
| DW66_1843 | molybdenum cofactor synthesis domain-containing protein | -4.13 | down |
| DW66_1844 | lipoprotein | -3.26 | down |
| DW66_1845 | hypothetical protein | -2.62 | down |
| DW66_1846 | cupin | -4.05 | down |
| DW66_1851 | drug resistance transporter EmrB | -1.45 | down |
| DW66_1852 | secretion protein HlyD | -1.69 | down |
| DW66_1853 | glutamyl-tRNA synthetase | -1.69 | down |
| DW66_1854 | transcriptional regulator TetR | -2.98 | down |
| DW66_1855 | hydrolase | -2.20 | down |
| DW66_1857 | thioesterase protein | -2.15 | down |
| DW66_1858 | dihydrouridine synthase DuS | -3.50 | down |
| DW66_1859 | heat shock protein Hsp20 | 5.76 | up |
| DW66_1860 | PAS/PAC sensor-containing diguanylate cyclase/phosphodiesterase | 3.59 | up |
| DW66_1864 | type 11 methyltransferase | -1.18 | down |
| DW66_1867 | hypothetical protein | -1.16 | down |
| DW66_1868 | aspartate-semialdehyde dehydrogenase | -1.37 | down |
| DW66_1869 | peptidoglycan-binding LysM | 1.27 | up |
| DW66_1870 | tRNA pseudouridine synthase A | -1.64 | down |
| DW66_1872 | acetyl-CoA carboxylase subunit beta | -1.04 | down |
| DW66_1873 | bifunctional folylpolyglutamate synthase/ dihydrofolate synthase | -2.41 | down |
| DW66_1874 | cell division protein | -1.71 | down |
| DW66_1875 | colicin V production protein | -1.33 | down |
| DW66_1878 | oxidoreductase | -1.20 | down |
| DW66_1879 | lipoprotein | -4.14 | down |
| DW66_1882 | transcriptional regulator AraC | -3.21 | down |
| DW66_1883 | nitrilase/cyanide hydratase and apolipoprotein N-acyltransferase | -2.95 | down |
| DW66_1884 | NADH:flavin oxidoreductase | -1.48 | down |
| DW66_1885 | 1-aminocyclopropane-1-carboxylate deaminase | -2.18 | down |
| DW66_1892 | hypothetical protein | -1.70 | down |
| DW66_1897 | 3-carboxymuconate cyclase | 2.22 | up |
| DW66_1898 | hypothetical protein | -3.23 | down |
| DW66_1899 | glutathione S-transferase | -2.75 | down |
| DW66_1900 | SMC domain-containing protein | -2.43 | down |
| DW66_1901 | nuclease SbcCD subunit D | -3.26 | down |
| DW66_1902 | type IV secretion protein Rhs | -2.84 | down |
| DW66_1903 | membrane protein | -2.75 | down |
| DW66_1904 | hypothetical protein | 2.44 | up |
| DW66_1907 | von Willebrand factor type A | -1.72 | down |
| DW66_1908 | hypothetical protein | -2.57 | down |
| DW66_1909 | hypothetical protein | -1.47 | down |
| DW66_1912 | hypothetical protein | -2.25 | down |
| DW66_1914 | benzoate transporter | -2.04 | down |
| DW66_1916 | dihydrodipicolinate synthetase | 1.16 | up |
| DW66_1917 | class II aldolase/adducin domain-containing protein | -3.76 | down |
| DW66_1918 | transmembrane protein | -2.69 | down |
| DW66_1919 | regulator domain-containing protein | -1.38 | down |
| DW66_1920 | NmrA protein | 2.13 | up |
| DW66_1921 | hypothetical protein | 2.30 | up |
| DW66_1923 | drug resistance transporter EmrB | -1.37 | down |
| DW66_1926 | transcriptional regulator | -3.50 | down |
| DW66_1927 | N-acetyltransferase GCN5 | -1.79 | down |
| DW66_1930 | DNA-binding transcriptional regulator LysR | -1.58 | down |
| DW66_1932 | hypothetical protein | -1.04 | down |
| DW66_1934 | NAD-glutamate dehydrogenase | 2.60 | up |
| DW66_1935 | PEP synthetase regulatory protein | 1.06 | up |
| DW66_1936 | hypothetical protein | -2.86 | down |
| DW66_1938 | Alpha/beta hydrolase | -1.58 | down |
| DW66_1941 | CrfX protein | 3.34 | up |
| DW66_1942 | TM helix repeat-containing protein | 1.91 | up |
| DW66_1945 | uroporphyrin-III C-methyltransferase | -4.65 | down |
| DW66_1946 | serine/threonine-protein kinase | -2.52 | down |
| DW66_1947 | MFS transporter | -3.41 | down |
| DW66_1948 | response regulator receiver/ANTAR domain-containing protein | -4.27 | down |
| DW66_1949 | nitrate-binding protein NasS | -2.70 | down |
| DW66_1950 | dihydroorotate dehydrogenase 2 | -2.27 | down |
| DW66_1955 | PAS/PAC sensor hybrid histidine kinase | -3.28 | down |
| DW66_1956 | transcriptional regulator | -8.61 | down |
| DW66_1961 | ammonium transporter | -4.80 | down |
| DW66_1962 | glutaredoxin | -1.73 | down |
| DW66_1963 | peptidase M15A | -3.07 | down |
| DW66_1965 | pseudouridine synthase | -3.10 | down |
| DW66_1968 | aconitate hydratase | 2.59 | up |
| DW66_1969 | RNA 2'-O-ribose methyltransferase | -3.90 | down |
| DW66_1971 | erythronate-4-phosphate dehydrogenase | -1.03 | down |
| DW66_1972 | hypothetical protein | 3.98 | up |
| DW66_1973 | hypothetical protein | 3.34 | up |
| DW66_1974 | lipid ABC transporter ATPase/inner membrane protein | -3.59 | down |
| DW66_1975 | hypothetical protein | -4.50 | down |
| DW66_1976 | methyl-accepting chemotaxis sensory transducer | -3.96 | down |
| DW66_1977 | malate:quinone oxidoreductase | -2.84 | down |
| DW66_1978 | lipoprotein | 1.31 | up |
| DW66_1981 | group 1 glycosyl transferase | 2.09 | up |
| DW66_1983 | transcriptional regulator | -2.43 | down |
| DW66_1984 | sensor histidine kinase | -3.61 | down |
| DW66_1985 | CheA signal transduction histidine kinase | -3.53 | down |
| DW66_1987 | MOSC domain-containing protein | -1.20 | down |
| DW66_1988 | lytic transglycosylase | 2.38 | up |
| DW66_1989 | ABC transporter ATP-binding protein | -3.32 | down |
| DW66_1990 | UspA domain-containing protein | 2.02 | up |
| DW66_1991 | hypothetical protein | -3.24 | down |
| DW66_1992 | fatty acid oxidation complex subunit alpha | -2.30 | down |
| DW66_1993 | 3-ketoacyl-CoA thiolase | -1.21 | down |
| DW66_1994 | hypothetical protein | -1.10 | down |
| DW66_2000 | transcriptional regulator TetR | -1.99 | down |
| DW66_2001 | ErfK/YbiS/YcfS/YnhG protein | -3.32 | down |
| DW66_2002 | *beta*-hexosaminidase | -3.05 | down |
| DW66_2003 | *beta*-hexosaminidase | -4.08 | down |
| DW66_2004 | non-specific serine/threonine protein kinase | 1.31 | up |
| DW66_2007 | glyceraldehyde-3-phosphate dehydrogenase | -1.10 | down |
| DW66_2008 | lipoprotein ApbE | -1.28 | down |
| DW66_2012 | lipoprotein releasing system, transmembrane protein | -2.51 | down |
| DW66_2013 | lipoprotein releasing system ATP-binding protein | -1.78 | down |
| DW66_2014 | lipoprotein releasing system, transmembrane protein | -2.83 | down |
| DW66_2017 | two component heavy metal response transcriptional regulator | -1.66 | down |
| DW66_2019 | chromosome segregation ATPase | 2.36 | up |
| DW66_2020 | Phosphonoacetaldehyde phosphonohydrolase-related protein | -4.63 | down |
| DW66_2025 | hypothetical protein | -2.85 | down |
| DW66_2028 | transcriptional regulator AraC | -4.24 | down |
| DW66_2029 | sterol desaturase | -4.12 | down |
| DW66_2032 | 2-pyrone-4,6-dicarboxylic acid hydrolase | -3.46 | down |
| DW66_2033 | transport-related membrane protein | -1.77 | down |
| DW66_2034 | hydroxyacid dehydrogenase | -1.53 | down |
| DW66_2036 | transport-related membrane protein | -2.78 | down |
| DW66_2037 | permease | -3.96 | down |
| DW66_2038 | SMP-30/gluconolaconase/LRE domain-containing protein | -3.61 | down |
| DW66_2039 | outer membrane porin | -1.66 | down |
| DW66_2042 | NADH dehydrogenase (quinone) | 1.87 | up |
| DW66_2043 | formate dehydrogenase subunit alpha | 2.43 | up |
| DW66_2044 | formate dehydrogenase subunit delta | 2.00 | up |
| DW66_2045 | UspA domain-containing protein | 3.10 | up |
| DW66_2046 | tRNA-hydroxylase | -3.85 | down |
| DW66_2048 | hypothetical protein | -2.48 | down |
| DW66_2050 | anti-FecI sigma factor FecR | -3.73 | down |
| DW66_2051 | TonB-dependent siderophore receptor | -3.35 | down |
| DW66_2052 | transcriptional regulator LysR | -1.01 | down |
| DW66_2053 | extracellular solute-binding protein | 1.19 | up |
| DW66_2055 | hypothetical protein | -1.05 | down |
| DW66_2057 | ATPase AFG1 | -1.24 | down |
| DW66_2058 | DinB protein | 2.70 | up |
| DW66_2060 | membrane protein | -1.15 | down |
| DW66_2063 | hypothetical protein | -5.48 | down |
| DW66_2064 | peptidase U32 | -3.25 | down |
| DW66_2065 | membrane protein | -1.44 | down |
| DW66_2066 | IS111A/IS1328/IS1533, transposase | 1.27 | up |
| DW66_2067 | phosphonoacetaldehyde hydrolase | -1.88 | down |
| DW66_2068 | 2-aminoethylphosphonate--pyruvate transaminase | -2.27 | down |
| DW66_2069 | transcriptional regulator LysR | -1.05 | down |
| DW66_2070 | transcriptional regulator AraC | -1.69 | down |
| DW66_2071 | hypothetical protein | -3.18 | down |
| DW66_2072 | Leucine-rich repeat domain protein | -2.38 | down |
| DW66_2075 | acetyl-CoA acetyltransferase | 1.54 | up |
| DW66_2076 | acyl-CoA dehydrogenase | 1.29 | up |
| DW66_2078 | transcriptional regulator TraR | -2.46 | down |
| DW66_2079 | hypothetical protein | -3.59 | down |
| DW66_2080 | hypothetical protein | -3.51 | down |
| DW66_2081 | hypothetical protein | -3.42 | down |
| DW66_2082 | monovalent cation/H+ antiporter subunit G | 1.42 | up |
| DW66_2083 | monovalent cation/H+ antiporter subunit F | 1.53 | up |
| DW66_2087 | monovalent cation/H+ antiporter subunit A | -1.20 | down |
| DW66_2088 | Permease of the drug/metabolite transporter | -3.50 | down |
| DW66_2089 | transcriptional regulator XRE | -2.37 | down |
| DW66_2090 | isochorismatase hydrolase | -3.23 | down |
| DW66_2091 | transcriptional regulator AraC | -1.15 | down |
| DW66_2092 | Chromosome segregation ATPases | -1.57 | down |
| DW66_2093 | hydrolase | -3.03 | down |
| DW66_2094 | Biotin carboxylase | -1.87 | down |
| DW66_2098 | ABC transporter | -1.83 | down |
| DW66_2099 | transporter protein | 1.15 | up |
| DW66_2100 | outer membrane receptor FepA | -7.23 | down |
| DW66_2101 | phosphoglycerate mutase | -1.61 | down |
| DW66_2102 | sulfate permease | -2.83 | down |
| DW66_2104 | FAD dependent oxidoreductase | 1.22 | up |
| DW66_2105 | endoribonuclease L-PSP | 1.12 | up |
| DW66_2107 | membrane protein | -1.54 | down |
| DW66_2108 | methyl-accepting chemotaxis sensory transducer | 1.78 | up |
| DW66_2109 | hypothetical protein | -1.84 | down |
| DW66_2111 | transmembrane pair domain-containing protein | -2.27 | down |
| DW66_2113 | transcriptional regulator | -1.49 | down |
| DW66_2114 | transcriptional regulator | -1.21 | down |
| DW66_2115 | C4-dicarboxylate transporter DctA | -1.25 | down |
| DW66_2117 | PAS/PAC sensor-containing diguanylate cyclase/phosphodiesterase | -2.27 | down |
| DW66_2121 | GAF modulated sigma54 specific transcriptional regulator | 1.03 | up |
| DW66_2122 | sugar ABC transporter ATP-binding protein | -3.51 | down |
| DW66_2123 | sugar ABC transporter ATP-binding protein | -1.94 | down |
| DW66_2124 | binding-protein-dependent transport system inner membrane protein | -5.22 | down |
| DW66_2125 | binding-protein-dependent transport system inner membrane protein | -2.69 | down |
| DW66_2128 | hypothetical protein | -1.20 | down |
| DW66_2130 | hypothetical protein | 1.88 | up |
| DW66_2131 | trigger factor | -1.57 | down |
| DW66_2133 | ATP-dependent protease, ATP-binding subunit ClpX | 1.68 | up |
| DW66_2134 | ATP-dependent protease La | 2.81 | up |
| DW66_2135 | DNA-binding protein | -1.08 | down |
| DW66_2136 | PpiC-type peptidyl-prolyl cis-trans isomerase | -1.72 | down |
| DW66_2140 | acyl-CoA thioesterase II | -2.05 | down |
| DW66_2141 | Phage terminase, small subunit | 3.00 | up |
| DW66_2142 | methyl-accepting chemotaxis sensory transducer | 1.94 | up |
| DW66_2143 | preprotein translocase subunit TatD | -2.39 | down |
| DW66_2144 | lytic transglycosylase | -3.09 | down |
| DW66_2145 | DoxX protein | -5.09 | down |
| DW66_2146 | hypothetical protein | -1.77 | down |
| DW66_2147 | transcription elongation factor GreB | -1.53 | down |
| DW66_2149 | ABC transporter ATP-binding protein | -1.54 | down |
| DW66_2151 | hypothetical protein | -4.21 | down |
| DW66_2153 | outer membrane lipoprotein OprI | 4.50 | up |
| DW66_2154 | hypothetical protein | 1.52 | up |
| DW66_2156 | hypothetical protein | -11.52 | down |
| DW66_2158 | UspA domain-containing protein | 3.35 | up |
| DW66_2161 | para-aminobenzoate synthase subunit I | -3.88 | down |
| DW66_2163 | hypothetical protein | -1.85 | down |
| DW66_2164 | hypothetical protein | -2.19 | down |
| DW66_2166 | 2-methylisocitrate lyase | 2.24 | up |
| DW66_2167 | hypothetical protein | -10.80 | down |
| DW66_2168 | methylcitrate synthase | 2.41 | up |
| DW66_2169 | hypothetical protein | -3.76 | down |
| DW66_2171 | aconitate hydratase | 2.44 | up |
| DW66_2172 | AcnD-accessory protein PrpF | 1.71 | up |
| DW66_2173 | 2-methylcitrate dehydratase | 2.00 | up |
| DW66_2176 | hypothetical protein | -1.94 | down |
| DW66_2178 | hypothetical protein | -3.70 | down |
| DW66_2180 | peptidase | -1.78 | down |
| DW66_2181 | peptidase | -2.91 | down |
| DW66_2182 | two component transcriptional regulator | -4.48 | down |
| DW66_2183 | integral membrane sensor signal transduction histidine kinase | -3.36 | down |
| DW66_2184 | hypothetical protein | -2.32 | down |
| DW66_2185 | transcriptional regulator LysR | -1.49 | down |
| DW66_2189 | hypothetical protein | 2.23 | up |
| DW66_2193 | spore coat U domain-containing protein | 1.98 | up |
| DW66_2196 | spore coat U domain-containing protein | -1.28 | down |
| DW66_2197 | type 1 pili usher pathway chaperone CsuC | -1.56 | down |
| DW66_2198 | fimbrial biogenesis outer membrane usher protein | -3.12 | down |
| DW66_2199 | spore coat U domain-containing protein | -2.86 | down |
| DW66_2203 | integral membrane protein TerC | -1.61 | down |
| DW66_2204 | hypothetical protein | -2.85 | down |
| DW66_2205 | lipoprotein VacJ | -3.32 | down |
| DW66_2206 | two component transcriptional regulator | -1.74 | down |
| DW66_2207 | integral membrane sensor signal transduction histidine kinase | -3.70 | down |
| DW66_2208 | hypothetical protein | 2.13 | up |
| DW66_2212 | sulfite reductase (ferredoxin) | -1.63 | down |
| DW66_2213 | hypothetical protein | -1.98 | down |
| DW66_2214 | periplasmic binding protein/LacI transcriptional regulator | -2.18 | down |
| DW66_2215 | hypothetical protein | 3.99 | up |
| DW66_2216 | hypothetical protein | 1.95 | up |
| DW66_2217 | B12-dependent methionine synthase | 1.26 | up |
| DW66_2218 | fatty acid cistrans isomerase | -1.50 | down |
| DW66_2219 | acyltransferase 3 | -2.96 | down |
| DW66_2220 | NfuA Fe-S protein maturation | 1.25 | up |
| DW66_2223 | hypothetical protein | 4.32 | up |
| DW66_2225 | transporter protein | -3.20 | down |
| DW66_2226 | ornithine decarboxylase, constitutive | -1.99 | down |
| DW66_2227 | transcriptional regulator AraC | -2.32 | down |
| DW66_2228 | branched chain amino acid ABC transporter | -2.16 | down |
| DW66_2229 | AzlC protein | -3.09 | down |
| DW66_2230 | hypothetical protein | -1.64 | down |
| DW66_2232 | lysine exporter protein LysE/YggA | -1.49 | down |
| DW66_2233 | hypothetical protein | -2.72 | down |
| DW66_2235 | DNA polymerase II | -1.35 | down |
| DW66_2236 | Leucine-rich repeat containing protein | -1.14 | down |
| DW66_2238 | hypothetical protein | -3.44 | down |
| DW66_2239 | hypothetical protein | -4.34 | down |
| DW66_2240 | hypothetical protein | -3.20 | down |
| DW66_2241 | hypothetical protein | -4.47 | down |
| DW66_2242 | ferric reductase domain-containing protein | -5.78 | down |
| DW66_2243 | integral membrane sensor signal transduction histidine kinase | -1.15 | down |
| DW66_2244 | two component transcriptional regulator | -1.32 | down |
| DW66_2245 | hypothetical protein | -2.73 | down |
| DW66_2246 | redoxin domain-containing protein | -3.63 | down |
| DW66_2248 | 3-dehydroquinate dehydratase | -1.67 | down |
| DW66_2249 | outer membrane efflux protein | -7.22 | down |
| DW66_2250 | RND efflux transporter, MFP subunit | -3.40 | down |
| DW66_2251 | heavy metal efflux protein | -3.15 | down |
| DW66_2253 | TonB-dependent siderophore receptor | -5.01 | down |
| DW66_2254 | hypothetical protein | -2.37 | down |
| DW66_2255 | cobyrinic acid a,c-diamide synthase | -1.92 | down |
| DW66_2256 | diguanylate cyclase | -2.22 | down |
| DW66_2257 | hypothetical protein | -1.68 | down |
| DW66_2258 | outer membrane porin | -3.35 | down |
| DW66_2259 | iron ABC transporter ATP-binding protein | -8.44 | down |
| DW66_2260 | transport system permease | -5.16 | down |
| DW66_2261 | periplasmic binding protein | -5.26 | down |
| DW66_2262 | membrane protein | -4.00 | down |
| DW66_2263 | TonB-dependent siderophore receptor | -5.51 | down |
| DW66_2264 | transcriptional regulator LysR | -3.25 | down |
| DW66_2265 | lipoprotein | 2.67 | up |
| DW66_2266 | integral membrane protein TerC | 2.31 | up |
| DW66_2268 | hypothetical protein | -1.11 | down |
| DW66_2269 | FAD dependent oxidoreductase | -3.07 | down |
| DW66_2270 | sporulation domain-containing protein | -1.02 | down |
| DW66_2271 | Guanosine-3',5'-bis(Diphosphate) 3'-pyrophosphohydrolase | 1.17 | up |
| DW66_2273 | glutaminase-asparaginase | -1.05 | down |
| DW66_2279 | D-ribose pyranase | -1.39 | down |
| DW66_2280 | inosine/uridine-preferring nucleoside hydrolase | -1.72 | down |
| DW66_2283 | cold shock protein CspA | 2.37 | up |
| DW66_2284 | hypothetical protein | 1.30 | up |
| DW66_2285 | threonyl-tRNA synthetase | -1.05 | down |
| DW66_2286 | translation initiation factor IF-3 | -2.70 | down |
| DW66_2287 | ribosomal protein L35 | -2.39 | down |
| DW66_2288 | 50S ribosomal protein L20 | -2.16 | down |
| DW66_2289 | phenylalanyl-tRNA synthetase subunit alpha | -3.38 | down |
| DW66_2293 | Transcriptional regulator | -1.33 | down |
| DW66_2297 | diguanylate cyclase | 2.54 | up |
| DW66_2299 | transcriptional regulator | -2.75 | down |
| DW66_2300 | dihydroxy-acid dehydratase | -1.35 | down |
| DW66_2301 | transporter protein | -2.64 | down |
| DW66_2302 | hypothetical protein | -2.14 | down |
| DW66_2303 | aldehyde dehydrogenase | -1.49 | down |
| DW66_2304 | hypothetical protein | 1.89 | up |
| DW66_2305 | hypothetical protein | 3.31 | up |
| DW66_2306 | hypothetical protein | -1.91 | down |
| DW66_2307 | hypothetical protein | -2.40 | down |
| DW66_2308 | hypothetical protein | -2.72 | down |
| DW66_2314 | oligopeptide transporter OPT | -1.50 | down |
| DW66_2315 | hypothetical protein | -1.79 | down |
| DW66_2318 | metal dependent phosphohydrolase | -2.45 | down |
| DW66_2320 | hypothetical protein | -3.55 | down |
| DW66_2321 | transcriptional regulator LysR | -1.15 | down |
| DW66_2322 | transporter protein | -1.43 | down |
| DW66_2323 | glutathione S-transferase | -1.31 | down |
| DW66_2324 | hypothetical protein | -2.49 | down |
| DW66_2325 | hypothetical protein | -2.55 | down |
| DW66_2326 | transcriptional regulator LysR | -2.54 | down |
| DW66_2329 | iron-containing alcohol dehydrogenase | -13.13 | down |
| DW66_2331 | hypothetical protein | -1.04 | down |
| DW66_2332 | short-chain dehydrogenase/reductase SDR | -2.01 | down |
| DW66_2333 | transcriptional factor-like protein | -2.44 | down |
| DW66_2334 | alcohol dehydrogenase | -2.50 | down |
| DW66_2335 | transporter protein | -5.79 | down |
| DW66_2336 | NADH:flavin oxidoreductase | -4.01 | down |
| DW66_2340 | transcriptional regulator LysR | -1.24 | down |
| DW66_2341 | transporter protein | -1.37 | down |
| DW66_2345 | transcriptional regulator | -1.55 | down |
| DW66_2346 | hypothetical protein | -1.80 | down |
| DW66_2347 | amino acid permease-associated protein | -2.02 | down |
| DW66_2348 | trans-aconitate 2-methyltransferase | 2.63 | up |
| DW66_2351 | transporter protein | -1.76 | down |
| DW66_2352 | aminophosphonate oxidoreductase | -1.75 | down |
| DW66_2353 | hypothetical protein | -2.27 | down |
| DW66_2354 | transcriptional regulator LysR | -3.69 | down |
| DW66_2356 | transporter protein | -1.68 | down |
| DW66_2357 | hypothetical protein | -1.76 | down |
| DW66_2358 | hypothetical protein | -3.11 | down |
| DW66_2361 | chromate transporter | -1.86 | down |
| DW66_2363 | type I secretion outer membrane protein TolC | 1.99 | up |
| DW66_2364 | type I secretion membrane fusion protein HlyD | 2.00 | up |
| DW66_2366 | heme peroxidase | -1.25 | down |
| DW66_2367 | transcriptional regulator LysR | -1.24 | down |
| DW66_2368 | hydrolase | -3.00 | down |
| DW66_2369 | pirin domain-containing protein | 4.01 | up |
| DW66_2371 | UvrD/REP helicase | -2.23 | down |
| DW66_2372 | Leucine-rich repeat domain protein | -1.54 | down |
| DW66_2373 | alpha/beta hydrolase fold protein | -1.02 | down |
| DW66_2374 | glyoxalase/bleomycin resistance protein/dioxygenase | -1.25 | down |
| DW66_2375 | alpha/beta hydrolase fold protein | -1.57 | down |
| DW66_2376 | transporter protein | 2.69 | up |
| DW66_2377 | hypothetical protein | 1.75 | up |
| DW66_2378 | Thermostable hemolysin delta-VPH | -3.97 | down |
| DW66_2379 | long-chain-fatty-acid-CoA ligase | -3.22 | down |
| DW66_2380 | long-chain acyl-CoA synthetase | -2.21 | down |
| DW66_2381 | short-chain dehydrogenase | -2.02 | down |
| DW66_2382 | hypothetical protein | -1.62 | down |
| DW66_2383 | transcriptional regulator | 1.01 | up |
| DW66_2385 | glyoxalase/bleomycin resistance protein/dioxygenase | 2.28 | up |
| DW66_2387 | hypothetical protein | 4.67 | up |
| DW66_2388 | paraquat-inducible protein A | 2.38 | up |
| DW66_2389 | paraquat-inducible protein A | 3.21 | up |
| DW66_2390 | Paraquat-inducible protein B | 3.25 | up |
| DW66_2391 | hypothetical protein | 3.86 | up |
| DW66_2392 | hypothetical protein | -5.33 | down |
| DW66_2393 | hypothetical protein | 1.52 | up |
| DW66_2395 | aldehyde dehydrogenase | -2.48 | down |
| DW66_2399 | aldehyde dehydrogenase | -1.68 | down |
| DW66_2401 | FAD-binding dehydrogenase | -1.39 | down |
| DW66_2402 | transcriptional regulator IclR | -1.39 | down |
| DW66_2405 | transporter protein | 1.07 | up |
| DW66_2406 | fumarate reductase/succinate dehydrogenase flavoprotein domain-containing protein | -2.27 | down |
| DW66_2408 | fumarate reductase/succinate dehydrogenase flavoprotein domain-containing protein | -1.64 | down |
| DW66_2409 | shikimate dehydrogenase | -1.80 | down |
| DW66_2411 | extracellular solute-binding protein | -2.52 | down |
| DW66_2412 | FAD dependent oxidoreductase | -3.19 | down |
| DW66_2413 | BFD/(2Fe-2S)-binding domain-containing protein | -3.44 | down |
| DW66_2414 | ferredoxin | -1.96 | down |
| DW66_2415 | binding-protein-dependent transport system inner membrane protein | -1.80 | down |
| DW66_2416 | binding-protein-dependent transport system inner membrane protein | -2.82 | down |
| DW66_2417 | ABC transporter ATP-binding protein | -1.29 | down |
| DW66_2418 | transcriptional regulator IclR | 2.57 | up |
| DW66_2424 | Outer membrane protein ImpK/VasF, OmpA/MotB domain | -2.51 | down |
| DW66_2426 | type VI secretion-associated protein ImpA | -3.26 | down |
| DW66_2427 | hypothetical protein | -5.76 | down |
| DW66_2428 | hypothetical protein | -3.49 | down |
| DW66_2429 | hypothetical protein | -2.77 | down |
| DW66_2430 | hypothetical protein | -2.04 | down |
| DW66_2432 | dihydrodipicolinate synthase | -1.72 | down |
| DW66_2434 | transcriptional regulator | -1.37 | down |
| DW66_2435 | hypothetical protein | -5.30 | down |
| DW66_2436 | methyl-accepting chemotaxis sensory transducer | -2.20 | down |
| DW66_2437 | hypothetical protein | -1.41 | down |
| DW66_2439 | Kup system potassium uptake protein | -2.21 | down |
| DW66_2440 | transporter protein | -1.97 | down |
| DW66_2441 | universal stress protein | 2.02 | up |
| DW66_2442 | hypothetical protein | 1.13 | up |
| DW66_2443 | Plasmid stabilization system protein | -3.06 | down |
| DW66_2444 | phosphate ABC transporter substrate-binding protein | -1.56 | down |
| DW66_2445 | phosphate ABC transporter permease | -2.27 | down |
| DW66_2446 | phosphate ABC transporter permease | -2.94 | down |
| DW66_2447 | Phosphate import ATP-binding protein pstB 1 | -1.36 | down |
| DW66_2449 | hypothetical protein | -2.05 | down |
| DW66_2450 | transcriptional regulator XRE | 1.90 | up |
| DW66_2451 | glutamine synthetase | 1.43 | up |
| DW66_2455 | short-chain dehydrogenase/reductase SDR | 3.44 | up |
| DW66_2456 | hypothetical protein | 1.72 | up |
| DW66_2459 | cyclopropane-fatty-acyl-phospholipid synthase | 1.36 | up |
| DW66_2460 | hypothetical protein | 1.28 | up |
| DW66_2461 | amine oxidase | 1.71 | up |
| DW66_2462 | short-chain dehydrogenase/reductase | 2.23 | up |
| DW66_2463 | hypothetical protein | 2.88 | up |
| DW66_2465 | transcriptional regulator MerR | -2.58 | down |
| DW66_2466 | Outer membrane protein (porin) | -3.82 | down |
| DW66_2467 | hypothetical protein | -1.83 | down |
| DW66_2468 | PAS/PAC sensor hybrid histidine kinase | -1.41 | down |
| DW66_2469 | transcriptional regulator | -2.00 | down |
| DW66_2470 | Rhodanese-related sulfurtransferase | -3.63 | down |
| DW66_2471 | ABC transporter | -2.07 | down |
| DW66_2472 | efflux ABC transporter ATP-binding protein | -1.48 | down |
| DW66_2473 | outer membrane protein | -1.40 | down |
| DW66_2474 | ABC-type branched-chain amino acid transport systems periplasmic compoment-like protein | -1.44 | down |
| DW66_2475 | integral membrane sensor signal transduction histidine kinase | -1.97 | down |
| DW66_2476 | transcriptional regulator | -1.99 | down |
| DW66_2477 | pentapeptide repeat-containing protein | -2.92 | down |
| DW66_2480 | extracellular solute-binding protein | -1.60 | down |
| DW66_2481 | hypothetical protein | -2.76 | down |
| DW66_2482 | *beta*-lactamase domain-containing protein | -2.83 | down |
| DW66_2483 | quinoprotein ethanol dehydrogenase | 1.40 | up |
| DW66_2484 | aldehyde dehydrogenase | 1.19 | up |
| DW66_2485 | pyrroloquinoline quinone biosynthesis protein PqqD | 1.18 | up |
| DW66_2486 | iron-containing alcohol dehydrogenase | -1.47 | down |
| DW66_2487 | PAS/PAC sensor hybrid histidine kinase | -1.58 | down |
| DW66_2488 | sulfate permease | 1.08 | up |
| DW66_2489 | RND transporter | -4.03 | down |
| DW66_2490 | RND efflux transporter, MFP subunit | -4.73 | down |
| DW66_2491 | hydrophobe/amphiphile efflux-1 | -2.99 | down |
| DW66_2493 | transporter protein | -2.32 | down |
| DW66_2496 | iron-containing alcohol dehydrogenase | 1.09 | up |
| DW66_2497 | short-chain dehydrogenase/reductase SDR | 2.25 | up |
| DW66_2498 | shikimate/quinate 5-dehydrogenase | 2.45 | up |
| DW66_2501 | carbohydrate-selective porin OprB | -2.27 | down |
| DW66_2502 | aldehyde dehydrogenase | -1.01 | down |
| DW66_2503 | sugar ABC transporter periplasmic protein | -2.89 | down |
| DW66_2504 | ribose ABC transporter ATP-binding protein | -3.39 | down |
| DW66_2507 | transporter | -2.40 | down |
| DW66_2509 | hypothetical protein | -3.20 | down |
| DW66_2511 | exodeoxyribonuclease III | -1.22 | down |
| DW66_2512 | hypothetical protein | -2.29 | down |
| DW66_2513 | mechanosensitive ion channel protein MscS | -1.94 | down |
| DW66_2514 | hypothetical protein | -1.06 | down |
| DW66_2517 | membrane protein | -6.93 | down |
| DW66_2518 | hypothetical protein | -3.29 | down |
| DW66_2519 | CinA domain-containing protein | -1.05 | down |
| DW66_2520 | hypothetical protein | -1.03 | down |
| DW66_2521 | alcohol dehydrogenase | -2.37 | down |
| DW66_2522 | integral membrane protein | -4.05 | down |
| DW66_2527 | transcriptional regulator AraC | -1.59 | down |
| DW66_2529 | creatininase | 1.84 | up |
| DW66_2530 | Transcriptional regulator | -2.53 | down |
| DW66_2532 | DSBH domain containing protein | -1.00 | down |
| DW66_2534 | transcriptional regulator LysR | -2.11 | down |
| DW66_2535 | alcohol dehydrogenase | -2.92 | down |
| DW66_2536 | transporter protein | -2.84 | down |
| DW66_2537 | glyceraldehyde-3-phosphate dehydrogenase | -2.06 | down |
| DW66_2538 | arsenical resistance protein ArsH | -2.33 | down |
| DW66_2539 | arsenical-resistance protein | -3.78 | down |
| DW66_2540 | transcriptional regulator ArsR | -3.75 | down |
| DW66_2541 | transcriptional regulator LysR | -2.04 | down |
| DW66_2542 | tricarballylate dehydrogenase | -2.83 | down |
| DW66_2543 | tricarballylate utilization protein B | -6.75 | down |
| DW66_2544 | transcriptional regulator LysR | -1.73 | down |
| DW66_2545 | hypothetical protein | -2.11 | down |
| DW66_2546 | sodium:dicarboxylate symporter | -1.97 | down |
| DW66_2547 | citrate transporter | -1.46 | down |
| DW66_2549 | permeases of the drug/metabolite transporter | -2.01 | down |
| DW66_2550 | diguanylate cyclase | -3.79 | down |
| DW66_2551 | NAD-dependent epimerase/dehydratase / Alpha/beta hydrolase | -5.31 | down |
| DW66_2553 | RNA polymerase | -2.29 | down |
| DW66_2554 | transmembrane sensor | -3.71 | down |
| DW66_2555 | Outer membrane receptor proteins, likely involved in siderophore uptake | -3.86 | down |
| DW66_2557 | short chain dehydrogenase/reductase oxidoreductase | -4.08 | down |
| DW66_2558 | transcriptional activator FtrA | -1.44 | down |
| DW66_2559 | rhodanese | -3.82 | down |
| DW66_2560 | Chorismate mutase | -2.89 | down |
| DW66_2561 | hypothetical protein | -11.82 | down |
| DW66_2562 | hypothetical protein | -3.85 | down |
| DW66_2563 | transcriptional regulator Cro/CI | -3.14 | down |
| DW66_2564 | acetyltransferase | -2.22 | down |
| DW66_2566 | 2-dehydropantoate 2-reductase | -15.52 | down |
| DW66_2568 | dehydratase | -2.59 | down |
| DW66_2569 | L-carnitine dehydratase/bile acid-inducible protein F | -3.48 | down |
| DW66_2570 | shikimate 5-dehydrogenase | -2.57 | down |
| DW66_2572 | transglutaminase | -3.39 | down |
| DW66_2573 | hypothetical protein | -3.00 | down |
| DW66_2574 | hypothetical protein | -3.58 | down |
| DW66_2575 | hypothetical protein | -3.02 | down |
| DW66_2576 | major facilitator transporter | -2.24 | down |
| DW66_2577 | sugar phosphate isomerase | -2.34 | down |
| DW66_2578 | transcriptional regulator MocR | -2.06 | down |
| DW66_2579 | hypothetical protein | -1.67 | down |
| DW66_2582 | histidine ABC transporter substrate-binding protein | -1.44 | down |
| DW66_2583 | glycine betaine ABC transporter permease | -2.08 | down |
| DW66_2584 | glycine betaine ABC transporter ATP-binding protein | -4.59 | down |
| DW66_2585 | histidine ammonia-lyase | -2.69 | down |
| DW66_2586 | stress-induced protein | 2.07 | up |
| DW66_2587 | short chain dehydrogenase | -1.28 | down |
| DW66_2588 | mechanosensitive ion channel protein MscS | 1.46 | up |
| DW66_2589 | membrane protein | -2.24 | down |
| DW66_2590 | transcriptional regulator CRP/FNR | -2.25 | down |
| DW66_2593 | hypothetical protein | -4.81 | down |
| DW66_2594 | UspA domain-containing protein | -1.38 | down |
| DW66_2595 | metallo-beta-lactamase | -2.11 | down |
| DW66_2596 | hypothetical protein | -2.55 | down |
| DW66_2597 | phosphoribosyl transferase | -2.61 | down |
| DW66_2598 | hypothetical protein | -1.52 | down |
| DW66_2599 | erythromycin esterase | -2.04 | down |
| DW66_2601 | hypothetical protein | -8.78 | down |
| DW66_2603 | heat-shock protein | 1.54 | up |
| DW66_2605 | phosphoketolase | -1.49 | down |
| DW66_2606 | ion transporter | -1.14 | down |
| DW66_2607 | ATP-dependent metalloprotease FtsH | -1.94 | down |
| DW66_2610 | ATPase P | -2.33 | down |
| DW66_2612 | molecular chaperone GroEL | -2.74 | down |
| DW66_2613 | universal stress protein | -3.40 | down |
| DW66_2615 | hypothetical protein | -1.65 | down |
| DW66_2616 | universal stress protein | -2.52 | down |
| DW66_2617 | universal stress protein | -2.18 | down |
| DW66_2618 | phosphoribosyltransferase | -2.40 | down |
| DW66_2619 | sodium:proton antiporter | -2.05 | down |
| DW66_2623 | hypothetical protein | 2.19 | up |
| DW66_2625 | short-chain dehydrogenase/reductase SDR | -2.58 | down |
| DW66_2626 | oxidoreductase | -1.89 | down |
| DW66_2628 | hypothetical protein | -1.21 | down |
| DW66_2629 | hypothetical protein | -2.48 | down |
| DW66_2630 | hypothetical protein | -3.08 | down |
| DW66_2631 | transposase mutator type | -3.35 | down |
| DW66_2632 | Mobile element protein | -1.86 | down |
| DW66_2633 | ATP-dependent endonuclease | 1.38 | up |
| DW66_2634 | transposase ISPs1 | -2.00 | down |
| DW66_2635 | relaxase | -2.43 | down |
| DW66_2636 | hypothetical protein | -2.75 | down |
| DW66_2641 | cobalamin synthesis protein P47K | -1.40 | down |
| DW66_2642 | bifunctional 3,4-dihydroxy-2-butanone 4-phosphate synthase/GTP cyclohydrolase II protein | -2.18 | down |
| DW66_2644 | ethyl tert-butyl ether degradation EthD | -4.98 | down |
| DW66_2645 | reductase | -1.96 | down |
| DW66_2646 | luciferase-like monooxygenase | -2.23 | down |
| DW66_2657 | transposase IS66 | -2.26 | down |
| DW66_2658 | Mobile element protein | -2.11 | down |
| DW66_2662 | transposase IS66 | -2.25 | down |
| DW66_2663 | Mobile element protein | -2.12 | down |
| DW66_2667 | taurine:2-oxoglutarate aminotransferase | -1.98 | down |
| DW66_2668 | demethylmenaquinone methyltransferase | -1.95 | down |
| DW66_2669 | endoribonuclease | -3.99 | down |
| DW66_2671 | MFS transporter | -2.45 | down |
| DW66_2672 | allantoate amidohydrolase | -1.44 | down |
| DW66_2674 | hypothetical protein | -13.26 | down |
| DW66_2675 | aldolase II | -2.66 | down |
| DW66_2676 | transporter | -2.70 | down |
| DW66_2677 | transcriptional regulator LysR | -1.28 | down |
| DW66_2678 | hypothetical protein | -1.83 | down |
| DW66_2679 | amino acid permease-associated protein | -4.41 | down |
| DW66_2682 | Amidinotransferase | -1.60 | down |
| DW66_2683 | transporter | -1.54 | down |
| DW66_2684 | integrase | 1.03 | up |
| DW66_2687 | hypothetical protein | -1.86 | down |
| DW66_2688 | transcriptional regulator | -2.84 | down |
| DW66_2689 | transporter protein | -3.26 | down |
| DW66_2690 | mandelate racemase/muconate lactonizing protein | -3.45 | down |
| DW66_2691 | transcriptional regulator LysR | -2.14 | down |
| DW66_2696 | hypothetical protein | -1.80 | down |
| DW66_2698 | hypothetical protein | -11.12 | down |
| DW66_2699 | HNH endonuclease | -11.64 | down |
| DW66_2701 | hypothetical protein | -2.64 | down |
| DW66_2702 | hypothetical protein | -2.05 | down |
| DW66_2704 | hypothetical protein | -11.99 | down |
| DW66_2705 | hypothetical protein | -3.18 | down |
| DW66_2706 | hypothetical protein | -1.56 | down |
| DW66_2707 | hypothetical protein | -3.33 | down |
| DW66_2709 | hypothetical protein | -3.95 | down |
| DW66_2710 | hypothetical protein | -2.86 | down |
| DW66_2711 | Prophage PssSM-02 protein | -11.58 | down |
| DW66_2712 | hypothetical protein | -2.16 | down |
| DW66_2713 | hypothetical protein | -11.67 | down |
| DW66_2716 | GAF sensor-containing diguanylate cyclase | -1.90 | down |
| DW66_2718 | repressor | -1.99 | down |
| DW66_2719 | hypothetical protein | -10.27 | down |
| DW66_2720 | hypothetical protein | -2.27 | down |
| DW66_2722 | DNA helicase | -2.99 | down |
| DW66_2723 | hypothetical protein | -1.72 | down |
| DW66_2724 | hypothetical protein | -1.34 | down |
| DW66_2725 | hypothetical protein | -2.18 | down |
| DW66_2726 | hypothetical protein | -10.25 | down |
| DW66_2730 | hypothetical protein | -1.16 | down |
| DW66_2732 | hypothetical protein | -2.73 | down |
| DW66_2733 | membrane protein | -3.36 | down |
| DW66_2734 | hypothetical protein | -1.97 | down |
| DW66_2735 | Phage terminase, small subunit | -1.79 | down |
| DW66_2736 | terminase | -3.55 | down |
| DW66_2737 | hypothetical protein | -1.98 | down |
| DW66_2738 | SPP1phage head morphogenesis protein | -2.03 | down |
| DW66_2741 | phage-related hypothetical protein | -1.65 | down |
| DW66_2742 | hypothetical protein | -5.58 | down |
| DW66_2743 | hypothetical protein | -11.81 | down |
| DW66_2744 | hypothetical protein | -9.89 | down |
| DW66_2745 | hypothetical protein | -1.72 | down |
| DW66_2746 | hypothetical protein | -1.19 | down |
| DW66_2747 | hypothetical protein | -1.97 | down |
| DW66_2749 | tail length tape measure protein | -1.45 | down |
| DW66_2750 | hypothetical protein | -1.90 | down |
| DW66_2751 | hypothetical protein | -2.26 | down |
| DW66_2752 | hypothetical protein | -2.23 | down |
| DW66_2753 | structural protein | -1.67 | down |
| DW66_2755 | hypothetical protein | -2.07 | down |
| DW66_2756 | hypothetical protein | -1.13 | down |
| DW66_2757 | hypothetical protein | -2.12 | down |
| DW66_2758 | hypothetical protein | -6.90 | down |
| DW66_2760 | transcriptional regulator LysR | -2.51 | down |
| DW66_2761 | galactarate dehydratase | -2.45 | down |
| DW66_2762 | malate/L-lactate dehydrogenase | -3.96 | down |
| DW66_2763 | fumarylacetoacetate (FAA) hydrolase | -7.50 | down |
| DW66_2764 | transporter protein | -1.19 | down |
| DW66_2765 | VRR-NUC domain-containing protein | -2.28 | down |
| DW66_2766 | DEAD/DEAH box helicase | -2.76 | down |
| DW66_2767 | NnrS protein | 4.40 | up |
| DW66_2768 | integrase | -3.65 | down |
| DW66_2769 | hypothetical protein | -5.52 | down |
| DW66_2770 | urease accessory protein UreD | -4.66 | down |
| DW66_2771 | urease subunit gamma | -3.77 | down |
| DW66_2772 | urease subunit beta | -1.83 | down |
| DW66_2773 | urease subunit alpha | -2.35 | down |
| DW66_2774 | urease accessory protein UreE | -1.98 | down |
| DW66_2776 | urease accessory protein UreF | -1.77 | down |
| DW66_2777 | urease accessory protein UreG | -2.18 | down |
| DW66_2778 | hypothetical protein | 1.19 | up |
| DW66_2779 | sulfatase | 1.82 | up |
| DW66_2780 | Fap amyloid fiber secretin | 2.17 | up |
| DW66_2781 | Fap system outer membrane protein | -2.04 | down |
| DW66_2782 | peptidase C39, bacteriocin processing | -3.57 | down |
| DW66_2783 | hypothetical protein | 1.76 | up |
| DW66_2784 | Fap amyloid fibril minor component | -3.61 | down |
| DW66_2785 | Fap unknown function protein | -3.91 | down |
| DW66_2786 | Ribosylnicotinamide kinase | -2.90 | down |
| DW66_2787 | nicotinamide mononucleotide transporter PnuC | -3.38 | down |
| DW66_2788 | methyl-accepting chemotaxis sensory transducer | -1.13 | down |
| DW66_2789 | undecaprenyl pyrophosphate phosphatase | -2.07 | down |
| DW66_2791 | hypothetical protein | -1.17 | down |
| DW66_2793 | azoreductase | 2.43 | up |
| DW66_2794 | FAD-dependent pyridine nucleotide-disulfide oxidoreductase | 2.45 | up |
| DW66_2795 | transcriptional regulator XRE | -1.49 | down |
| DW66_2798 | aldolase II | 1.03 | up |
| DW66_2800 | hypothetical protein | -1.43 | down |
| DW66_2801 | hypothetical protein | 9.47 | up |
| DW66_2802 | hypothetical protein | 1.76 | up |
| DW66_2803 | hypothetical protein | 5.17 | up |
| DW66_2804 | beta-lactamase | -1.73 | down |
| DW66_2810 | transcriptional regulator XRE | -1.28 | down |
| DW66_2811 | transporter protein | -1.73 | down |
| DW66_2819 | hypothetical protein | -4.92 | down |
| DW66_2822 | hypothetical protein | -2.54 | down |
| DW66_2823 | lipoprotein | -1.92 | down |
| DW66_2825 | hypothetical protein | 3.53 | up |
| DW66_2826 | hypothetical protein | 4.26 | up |
| DW66_2828 | hypothetical protein | -2.98 | down |
| DW66_2829 | transcriptional regulator LysR | -2.33 | down |
| DW66_2830 | transmembrane pair domain-containing protein | 5.51 | up |
| DW66_2832 | ABC transporter-like protein | -3.03 | down |
| DW66_2833 | hypothetical protein | -2.08 | down |
| DW66_2834 | cobalamin biosynthesis protein CobW | -4.75 | down |
| DW66_2835 | cobaltochelatase subunit CobN | -3.96 | down |
| DW66_2837 | ChlD component of cobalt chelatase involved in B12 biosynthesis | -1.25 | down |
| DW66_2838 | hypothetical protein | 2.16 | up |
| DW66_2841 | lipoprotein signal peptide | -2.79 | down |
| DW66_2843 | ABC transporte, ATP binding protein | -1.73 | down |
| DW66_2845 | response regulator receiver domain-containing protein | 1.38 | up |
| DW66_2850 | hypothetical protein | -1.60 | down |
| DW66_2851 | secretion protein HlyD | -3.02 | down |
| DW66_2853 | dioxygenase | -4.81 | down |
| DW66_2854 | sodium:dicarboxylate symporter | -1.59 | down |
| DW66_2855 | CigR (inner membrane protein) | 2.49 | up |
| DW66_2856 | hypothetical protein | -1.07 | down |
| DW66_2857 | hypothetical protein | -1.43 | down |
| DW66_2859 | hypothetical protein | 3.78 | up |
| DW66_2860 | alcohol dehydrogenase | -1.47 | down |
| DW66_2861 | taurine dioxygenase | -3.24 | down |
| DW66_2862 | Transposase | -3.15 | down |
| DW66_2863 | transporter protein | -4.93 | down |
| DW66_2864 | DSBA oxidoreductase | -5.84 | down |
| DW66_2865 | (2Fe-2S)-binding protein | -4.88 | down |
| DW66_2866 | membrane protein | -3.36 | down |
| DW66_2867 | transcriptional regulator AraC | -1.15 | down |
| DW66_2868 | hypothetical protein | -3.06 | down |
| DW66_2869 | hypothetical protein | -3.91 | down |
| DW66_2870 | hypothetical protein | -2.24 | down |
| DW66_2872 | transporter | -2.68 | down |
| DW66_2873 | acyl-CoA dehydrogenase domain-containing protein | -2.11 | down |
| DW66_2874 | acyl-CoA synthetase | -2.10 | down |
| DW66_2876 | hypothetical protein | -4.84 | down |
| DW66_2877 | nitrilase/cyanide hydratase and apolipoprotein N-acyltransferase | -4.57 | down |
| DW66_2880 | lysine exporter protein LysE/YggA | -3.25 | down |
| DW66_2881 | transcriptional regulator AraC | -1.81 | down |
| DW66_2883 | outer membrane autotransporter | -2.25 | down |
| DW66_2884 | PpiC-type peptidyl-prolyl cis-trans isomerase | -1.75 | down |
| DW66_2885 | acetoacetyl-CoA synthetase | 1.74 | up |
| DW66_2887 | beta-D-hydroxybutyrate dehydrogenase | 1.05 | up |
| DW66_2888 | citrate transporter | -2.25 | down |
| DW66_2890 | binding-protein-dependent transport system inner membrane protein | 1.79 | up |
| DW66_2895 | hypothetical protein | -4.23 | down |
| DW66_2899 | deoxyribonuclease I | -4.09 | down |
| DW66_2900 | transporter protein | -2.50 | down |
| DW66_2901 | transcriptional regulator LysR | -1.90 | down |
| DW66_2903 | amino acid permease | -2.09 | down |
| DW66_2904 | nitrilase/cyanide hydratase and apolipoprotein N-acyltransferase | -2.42 | down |
| DW66_2905 | transcriptional regulator | -1.51 | down |
| DW66_2906 | Thymidylate kinase | -2.82 | down |
| DW66_2907 | hypothetical protein | 3.98 | up |
| DW66_2912 | transcriptional regulator LysR | -2.28 | down |
| DW66_2913 | patatin | 2.04 | up |
| DW66_2914 | thiamine pyrophosphate protein | -1.70 | down |
| DW66_2915 | double-stranded beta helix domain-containing protein | -2.69 | down |
| DW66_2917 | hypothetical protein | -1.58 | down |
| DW66_2919 | hypothetical protein | -13.43 | down |
| DW66_2920 | hypothetical protein | 1.25 | up |
| DW66_2921 | hypothetical protein | -2.84 | down |
| DW66_2923 | hypothetical protein | -1.35 | down |
| DW66_2925 | transcriptional regulator LysR | -1.45 | down |
| DW66_2929 | transcriptional regulator | 2.65 | up |
| DW66_2931 | hypothetical protein | 1.95 | up |
| DW66_2932 | Cytochrome c | -3.40 | down |
| DW66_2935 | transcriptional regulator AsnC | 1.69 | up |
| DW66_2936 | isochorismatase hydrolase | 1.34 | up |
| DW66_2940 | hypothetical protein | -1.14 | down |
| DW66_2943 | transcriptional regulator PaaX | -1.52 | down |
| DW66_2944 | phenylacetic acid degradation protein PaaY | -2.08 | down |
| DW66_2945 | enoyl-CoA hydratase-isomerase | -1.28 | down |
| DW66_2947 | 3-hydroxyacyl-CoA dehydrogenase | -1.32 | down |
| DW66_2948 | phenylacetic acid degradation protein PaaD | 1.02 | up |
| DW66_2950 | phenylacetate-CoA ligase | -1.48 | down |
| DW66_2951 | phenylacetate-CoA oxygenase subunit PaaA | 1.77 | up |
| DW66_2952 | phenylacetate-CoA oxygenase subunit PaaB | 2.73 | up |
| DW66_2953 | phenylacetate-CoA oxygenase subunit PaaI | 2.56 | up |
| DW66_2954 | phenylacetate-CoA oxygenase subunit PaaJ | 1.63 | up |
| DW66_2955 | phenylacetate-CoA oxygenase/reductase subunit PaaK | 1.34 | up |
| DW66_2959 | bifunctional aldehyde dehydrogenase/enoyl-CoA hydratase | 1.72 | up |
| DW66_2966 | hypothetical protein | -1.31 | down |
| DW66_2970 | Inner membrane protein YbhQ | -1.41 | down |
| DW66_2975 | acyl-CoA dehydrogenase | -1.19 | down |
| DW66_2976 | LmbE protein | -2.30 | down |
| DW66_2977 | type 12 methyltransferase | -1.11 | down |
| DW66_2978 | glycosyl transferase group 2 protein | -2.13 | down |
| DW66_2979 | hypothetical protein | -1.14 | down |
| DW66_2980 | oxidoreductase | -2.66 | down |
| DW66_2981 | nucleoside phosphorylase-like protein | -2.61 | down |
| DW66_2982 | carboxylate-amine ligase | -4.03 | down |
| DW66_2983 | methyltransferase small | -2.77 | down |
| DW66_2984 | hypothetical protein | -1.99 | down |
| DW66_2985 | hypothetical protein | -2.14 | down |
| DW66_2987 | transporter protein | -1.77 | down |
| DW66_2989 | peroxidase | -2.82 | down |
| DW66_2991 | *beta*-carotene hydroxylase | -3.66 | down |
| DW66_2992 | magnesium transporter MgtC | -6.38 | down |
| DW66_2993 | N-acetyltransferase GCN5 | -4.59 | down |
| DW66_2994 | diguanylate cyclase | 1.80 | up |
| DW66_2996 | Fructose-2,6-bisphosphatase | -1.06 | down |
| DW66_2998 | N-isopropylammelide isopropylaminohydrolase | 1.56 | up |
| DW66_2999 | transcription regulator TenA | -3.24 | down |
| DW66_3000 | transcription regulator TenA | -3.48 | down |
| DW66_3001 | multicopper oxidase type 2 | -2.24 | down |
| DW66_3002 | electron transport protein SCO1/SenC | -5.57 | down |
| DW66_3003 | diguanylate cyclase/phosphodiesterase | -3.79 | down |
| DW66_3004 | integrase | -2.45 | down |
| DW66_3005 | SMP-30/gluconolaconase/LRE domain-containing protein | -1.22 | down |
| DW66_3006 | glycerate kinase | -3.97 | down |
| DW66_3007 | transcriptional regulator | -2.89 | down |
| DW66_3008 | hypothetical protein | -2.52 | down |
| DW66_3009 | transporter protein | -1.81 | down |
| DW66_3010 | FAD-dependent pyridine nucleotide-disulfide oxidoreductase | -2.19 | down |
| DW66_3012 | N-ethylmaleimide reductase | -2.76 | down |
| DW66_3013 | luciferase | -1.77 | down |
| DW66_3015 | TraX protein | -2.36 | down |
| DW66_3017 | outer membrane porin | -4.05 | down |
| DW66_3018 | membrane protein | -7.40 | down |
| DW66_3019 | catechol 1,2-dioxygenase | -5.78 | down |
| DW66_3020 | transporter protein | -3.83 | down |
| DW66_3021 | 1,6-dihydroxycyclohexa-2,4-diene-1-carboxylate dehydrogenase | -5.27 | down |
| DW66_3022 | hypothetical protein | -10.91 | down |
| DW66_3023 | oxidoreductase, FAD/NAD(P)-binding subunit | -1.74 | down |
| DW66_3024 | benzoate 1,2-dioxygenase small subunit | -4.31 | down |
| DW66_3025 | benzoate dioxygenase subunit alpha | -2.14 | down |
| DW66_3027 | lipoprotein | -2.53 | down |
| DW66_3028 | histidinol-phosphate phosphatase | -3.00 | down |
| DW66_3029 | UspA domain-containing protein | 3.05 | up |
| DW66_3030 | TonB-dependent siderophore receptor | -2.89 | down |
| DW66_3032 | hypothetical protein | 1.71 | up |
| DW66_3033 | transcriptional regulator LysR | 1.21 | up |
| DW66_3034 | aldehyde dehydrogenase | 3.60 | up |
| DW66_3035 | helicase | -2.80 | down |
| DW66_3036 | transcriptional regulator AraC | -1.46 | down |
| DW66_3037 | glutamate-putrescine ligase | -2.51 | down |
| DW66_3038 | extracellular solute-binding protein | -3.41 | down |
| DW66_3039 | FAD dependent oxidoreductase | -2.32 | down |
| DW66_3040 | outer membrane lipoprotein | 4.29 | up |
| DW66_3041 | L-serine dehydratase | -3.73 | down |
| DW66_3042 | transcriptional regulator LysR | -1.43 | down |
| DW66_3043 | exopolysaccharide biosynthesis polyprenyl glycosylphosphotransferase | 1.38 | up |
| DW66_3045 | glycosyl transferase | 1.16 | up |
| DW66_3046 | group 1 glycosyl transferase | 1.42 | up |
| DW66_3047 | VirK | 2.00 | up |
| DW66_3048 | glycosyl transferase | 2.55 | up |
| DW66_3049 | serine O-acetyltransferase | 3.42 | up |
| DW66_3050 | glycosyl transferase | 3.47 | up |
| DW66_3051 | hexapeptide repeat-containing transferase | 3.36 | up |
| DW66_3052 | GMC oxidoreductase | 1.84 | up |
| DW66_3053 | hypothetical protein | 1.76 | up |
| DW66_3056 | hypothetical protein | 2.56 | up |
| DW66_3058 | non-specific protein-tyrosine kinase | 3.72 | up |
| DW66_3059 | lipopolysaccharide biosynthesis protein | 1.14 | up |
| DW66_3061 | polysaccharide export protein | 1.35 | up |
| DW66_3063 | short-chain fatty acid transporter | -1.24 | down |
| DW66_3064 | 3-oxoacid CoA-transferase subunit B | 1.93 | up |
| DW66_3066 | transcriptional regulator LysR | -2.30 | down |
| DW66_3067 | aldo/keto reductase | -2.36 | down |
| DW66_3068 | error-prone DNA polymerase | -2.30 | down |
| DW66_3069 | DNA polymerase-like protein | -3.23 | down |
| DW66_3070 | RecA/RadA recombinase | -2.38 | down |
| DW66_3071 | LexA repressor | -1.36 | down |
| DW66_3072 | threonine efflux protein | -4.18 | down |
| DW66_3077 | amidase | -3.04 | down |
| DW66_3078 | transporter protein | -4.10 | down |
| DW66_3079 | amidohydrolase | -3.13 | down |
| DW66_3081 | YD repeat-containing protein | -2.01 | down |
| DW66_3082 | hypothetical protein | -5.65 | down |
| DW66_3083 | hypothetical protein | -3.16 | down |
| DW66_3084 | Rhs element Vgr protein | -2.90 | down |
| DW66_3085 | hypothetical protein | -2.44 | down |
| DW66_3087 | hypothetical protein | -3.79 | down |
| DW66_3088 | hypothetical protein | -1.18 | down |
| DW66_3089 | hypothetical protein | -2.24 | down |
| DW66_3090 | hypothetical protein | -2.94 | down |
| DW66_3092 | hypothetical protein | -4.56 | down |
| DW66_3093 | type VI secretion protein | -3.42 | down |
| DW66_3094 | type VI secretion protein EvpB | -3.30 | down |
| DW66_3095 | type VI secretion system lysozyme-like protein | -3.86 | down |
| DW66_3096 | type VI secretion protein | -5.20 | down |
| DW66_3097 | hypothetical protein | -3.44 | down |
| DW66_3098 | type VI secretion ATPase | -3.50 | down |
| DW66_3099 | lipoprotein | -5.38 | down |
| DW66_3100 | type VI secretion protein | -4.82 | down |
| DW66_3101 | hypothetical protein | -3.63 | down |
| DW66_3102 | ImcF domain-containing protein | -3.65 | down |
| DW66_3103 | hypothetical protein | -3.45 | down |
| DW66_3104 | OmpA/MotB domain-containing protein | -4.61 | down |
| DW66_3106 | type VI secretion-associated protein ImpA | -2.16 | down |
| DW66_3108 | hypothetical protein | -1.69 | down |
| DW66_3109 | auxin efflux carrier | -2.31 | down |
| DW66_3110 | hypothetical protein | -12.05 | down |
| DW66_3111 | MFS transporter | -2.85 | down |
| DW66_3115 | xylose isomerase domain-containing protein TIM barrel | -1.28 | down |
| DW66_3116 | transcriptional regulator LacI | -2.97 | down |
| DW66_3118 | gluconate 2-dehydrogenase | -2.35 | down |
| DW66_3119 | Gluconate 2-dehydrogenase acceptor subunit | -4.01 | down |
| DW66_3121 | Outer membrane protein ImpK/VasF, OmpA/MotB domain | -3.21 | down |
| DW66_3122 | Rhs element Vgr protein | -2.14 | down |
| DW66_3123 | hypothetical protein | -2.74 | down |
| DW66_3124 | hypothetical protein | -2.51 | down |
| DW66_3125 | hypothetical protein | -6.33 | down |
| DW66_3126 | Glycoprotein gp2 | -1.70 | down |
| DW66_3127 | Glycoprotein gp2 | -1.81 | down |
| DW66_3130 | 2OG-Fe(II) oxygenase | -2.03 | down |
| DW66_3131 | hypothetical protein | -1.27 | down |
| DW66_3132 | membrane protein | -1.11 | down |
| DW66_3133 | hypothetical protein | 2.15 | up |
| DW66_3134 | alcohol dehydrogenase | -2.61 | down |
| DW66_3135 | IS111A/IS1328/IS1533, transposase | 1.27 | up |
| DW66_3136 | pirin domain-containing protein | 1.68 | up |
| DW66_3137 | hypothetical protein | -2.24 | down |
| DW66_3138 | alpha/beta hydrolase fold protein | -2.11 | down |
| DW66_3139 | lysine exporter protein LysE/YggA | -1.22 | down |
| DW66_3141 | hypothetical protein | -3.54 | down |
| DW66_3142 | cobalt transporter, CbtB subunit | -2.17 | down |
| DW66_3143 | cobalt transporter, CbtA subunit | -1.91 | down |
| DW66_3144 | cobalamin biosynthesis protein cobE | -3.75 | down |
| DW66_3145 | precorrin-4 C(11)-methyltransferase | -3.95 | down |
| DW66_3146 | hypothetical protein | -2.34 | down |
| DW66_3147 | transcriptional regulator | 1.37 | up |
| DW66_3149 | pili assembly chaperone | 1.36 | up |
| DW66_3150 | transcriptional regulator LacI | -4.38 | down |
| DW66_3151 | gluconokinase | -2.01 | down |
| DW66_3153 | hypothetical protein | -3.54 | down |
| DW66_3157 | integral membrane sensor signal transduction histidine kinase | 1.30 | up |
| DW66_3158 | lytic transglycosylase | -1.61 | down |
| DW66_3159 | general secretion pathway protein G | -3.43 | down |
| DW66_3160 | type II secretion system protein | -2.46 | down |
| DW66_3161 | RND efflux transporter, MFP subunit | -2.36 | down |
| DW66_3163 | RND efflux system outer membrane lipoprotein | 1.23 | up |
| DW66_3165 | histidine kinase | -1.14 | down |
| DW66_3167 | ThiJ/PfpI domain-containing protein | 2.86 | up |
| DW66_3168 | Rtn protein | -1.53 | down |
| DW66_3172 | transporter | -2.38 | down |
| DW66_3173 | membrane protein | -1.13 | down |
| DW66_3174 | lysine exporter protein LysE/YggA | -1.68 | down |
| DW66_3178 | Leucine-rich repeat domain protein | -2.42 | down |
| DW66_3179 | aldehyde dehydrogenase | -1.30 | down |
| DW66_3180 | glyoxalase/bleomycin resistance protein/dioxygenase | 1.73 | up |
| DW66_3182 | threonine dehydratase | -2.93 | down |
| DW66_3183 | transmembrane pair domain-containing protein | -1.85 | down |
| DW66_3184 | hypothetical protein | -5.70 | down |
| DW66_3185 | bacteriophage N4 adsorption protein B | 1.87 | up |
| DW66_3186 | bacteriophage N4 receptor, outer membrane subunit | 3.70 | up |
| DW66_3187 | hypothetical protein | 4.35 | up |
| DW66_3188 | diguanylate cyclase | 3.70 | up |
| DW66_3190 | integral membrane sensor signal transduction histidine kinase | -1.70 | down |
| DW66_3191 | two component transcriptional regulator | -1.71 | down |
| DW66_3195 | molybdopterin-guanine dinucleotide biosynthesis protein MobA | -2.63 | down |
| DW66_3197 | Quinohemoprotein amine dehydrogenase beta subunit | 3.76 | up |
| DW66_3200 | quinohemoprotein amine dehydrogenase, 60 kDa subunit | -1.26 | down |
| DW66_3201 | hypothetical protein | -10.18 | down |
| DW66_3204 | hypothetical protein | -3.25 | down |
| DW66_3205 | monooxygenase FAD-binding protein | -1.93 | down |
| DW66_3207 | efflux ABC transporter ATP-binding protein | -1.70 | down |
| DW66_3208 | GAF modulated sigma54 specific transcriptional regulator | -1.40 | down |
| DW66_3209 | hypothetical protein | -2.17 | down |
| DW66_3210 | curli production assembly/transport component CsgG | -1.98 | down |
| DW66_3211 | curli production assembly/transport component CsgF | -1.73 | down |
| DW66_3212 | curli assembly protein CsgE | -2.72 | down |
| DW66_3213 | secretion system X pseudopilin PulG-like protein | -3.08 | down |
| DW66_3214 | type II secretion system protein G | -4.54 | down |
| DW66_3215 | type II and III secretion system protein | -4.00 | down |
| DW66_3216 | type II and III secretion system protein | -3.70 | down |
| DW66_3217 | secretion system X translation initiation factor | -3.45 | down |
| DW66_3218 | secretion system X transmembrane protein | -6.25 | down |
| DW66_3219 | secretion system X transmembrane protein | -5.24 | down |
| DW66_3220 | hypothetical protein | -3.56 | down |
| DW66_3221 | type II secretion system protein E | -4.80 | down |
| DW66_3222 | response regulator receiver protein | -1.81 | down |
| DW66_3224 | cytochrome c | -2.99 | down |
| DW66_3225 | electron transport protein SCO1/SenC | -1.68 | down |
| DW66_3227 | Collagen triple helix repeat domain protein | -2.03 | down |
| DW66_3228 | hypothetical protein | -1.75 | down |
| DW66_3229 | hypothetical protein | 1.31 | up |
| DW66_3230 | malonate transporter subunit MadM | -2.08 | down |
| DW66_3233 | phosphoribosyl-dephospho-CoA transferase | 1.76 | up |
| DW66_3240 | hypothetical protein | -10.52 | down |
| DW66_3243 | transcriptional regulator LysR | -2.74 | down |
| DW66_3244 | hypothetical protein | -1.04 | down |
| DW66_3245 | tRNA-m1A22 methylase | -4.63 | down |
| DW66_3247 | hypothetical protein | -1.83 | down |
| DW66_3248 | U32 peptidase | -1.17 | down |
| DW66_3249 | amidase | 1.07 | up |
| DW66_3250 | YheO domain-containing protein | -1.17 | down |
| DW66_3252 | carboxynorspermidine decarboxylase | -3.92 | down |
| DW66_3253 | saccharopine dehydrogenase | -2.43 | down |
| DW66_3254 | ThiJ/PfpI domain-containing protein | -2.35 | down |
| DW66_3256 | UDP-glucose 6-dehydrogenase | -2.94 | down |
| DW66_3257 | malate:quinone oxidoreductase | 4.22 | up |
| DW66_3259 | Domain often clustered or fused with uracil-DNA glycosylase | -1.73 | down |
| DW66_3261 | hypothetical protein | -3.11 | down |
| DW66_3262 | riboflavin synthase subunit alpha | -1.52 | down |
| DW66_3263 | hypothetical protein | -1.50 | down |
| DW66_3265 | *delta*-aminolevulinic acid dehydratase | -2.58 | down |
| DW66_3268 | hydroxyglutarate oxidase | 2.15 | up |
| DW66_3269 | Carbon starvation induced protein CsiD | 4.23 | up |
| DW66_3271 | two component transcriptional regulator | -4.93 | down |
| DW66_3272 | integral membrane sensor signal transduction histidine kinase | -3.30 | down |
| DW66_3275 | hypothetical protein | -1.28 | down |
| DW66_3277 | UDP-2,3-diacylglucosamine hydrolase | -1.21 | down |
| DW66_3278 | hypothetical protein | -5.23 | down |
| DW66_3279 | penicillin amidase | -3.45 | down |
| DW66_3282 | hypothetical protein | -6.20 | down |
| DW66_3283 | hypothetical protein | -2.38 | down |
| DW66_3284 | hypothetical protein | -2.58 | down |
| DW66_3285 | hypothetical protein | -6.48 | down |
| DW66_3286 | hypothetical protein | -2.50 | down |
| DW66_3287 | surface antigen (D15) | -2.46 | down |
| DW66_3288 | acetyltransferase | 2.08 | up |
| DW66_3291 | aliphatic sulfonates ABC transporter substrate-binding protein | -4.37 | down |
| DW66_3292 | monooxygenase | -2.22 | down |
| DW66_3294 | lipid-binding START domain-containing protein | -1.29 | down |
| DW66_3295 | transcriptional regulator LysR | -2.47 | down |
| DW66_3296 | gamma-glutamyltransferase | -1.95 | down |
| DW66_3298 | 4-hydroxybenzoate 3-monooxygenase | -1.06 | down |
| DW66_3299 | transcriptional regulator AraC | -1.27 | down |
| DW66_3304 | cytochrome c oxidase accessory protein CcoG | -1.72 | down |
| DW66_3310 | electron transport complex protein RnfC | -1.82 | down |
| DW66_3311 | RnfABCDGE type electron transport complex subunit D | -2.96 | down |
| DW66_3312 | drug resistance transporter EmrB | -1.79 | down |
| DW66_3313 | secretion protein HlyD | -3.43 | down |
| DW66_3314 | transcriptional regulator MarR | -5.08 | down |
| DW66_3315 | DNA-binding response regulator | -1.82 | down |
| DW66_3316 | PAS/PAC sensor signal transduction histidine kinase | -1.50 | down |
| DW66_3317 | acyl-CoA synthetase | -2.35 | down |
| DW66_3318 | acyl-CoA dehydrogenase domain-containing protein | -2.06 | down |
| DW66_3320 | Na+/H+ antiporter NhaC | -4.74 | down |
| DW66_3322 | glycine betaine ABC transporter substrate-binding protein | 2.08 | up |
| DW66_3323 | ABC transporter permease | 1.19 | up |
| DW66_3324 | transcriptional regulator LysR | -1.12 | down |
| DW66_3327 | transmembrane protein | -2.82 | down |
| DW66_3329 | lysine exporter protein LysE/YggA | -4.26 | down |
| DW66_3331 | transcriptional regulator LysR | -2.82 | down |
| DW66_3336 | acetylornithine deacetylase | -3.73 | down |
| DW66_3337 | major pilin protein fimA | -1.83 | down |
| DW66_3339 | monooxygenase | -1.49 | down |
| DW66_3341 | TonB-dependent siderophore receptor | -3.25 | down |
| DW66_3342 | anti-FecI sigma factor FecR | -3.63 | down |
| DW66_3343 | RNA polymerase sigma-29 factor | -3.16 | down |
| DW66_3346 | hypothetical protein | 2.79 | up |
| DW66_3347 | diguanylate cyclase/phosphodiesterase | -4.49 | down |
| DW66_3348 | RND efflux system outer membrane lipoprotein | -1.91 | down |
| DW66_3349 | acriflavin resistance protein | -1.67 | down |
| DW66_3350 | acriflavin resistance protein | -3.11 | down |
| DW66_3351 | RND efflux transporter, MFP subunit | -5.18 | down |
| DW66_3352 | redoxin domain-containing protein | 1.60 | up |
| DW66_3353 | aromatic amino acid permease | -2.69 | down |
| DW66_3355 | hypothetical protein | -3.20 | down |
| DW66_3357 | transcriptional regulator RpiR | 3.90 | up |
| DW66_3358 | amino acid ABC transporter periplasmic binding protein | 4.13 | up |
| DW66_3361 | FAD dependent oxidoreductase | 1.55 | up |
| DW66_3362 | amino acid ABC transporter ATP-binding protein | 1.60 | up |
| DW66_3363 | peptidase C26 | 1.81 | up |
| DW66_3364 | 5-dehydro-4-deoxyglucarate dehydratase | -4.00 | down |
| DW66_3365 | D-galactonate transporter | -2.63 | down |
| DW66_3366 | galactarate dehydratase | -3.13 | down |
| DW66_3369 | hypothetical protein | -3.58 | down |
| DW66_3370 | Valyl-tRNA synthetase | -4.90 | down |
| DW66_3371 | transcriptional regulator LysR | -2.83 | down |
| DW66_3372 | alcohol dehydrogenase | -3.61 | down |
| DW66_3374 | Integral membrane protein | -2.13 | down |
| DW66_3375 | hypothetical protein | 2.20 | up |
| DW66_3376 | TonB-dependent siderophore receptor | -3.67 | down |
| DW66_3377 | hypothetical protein | -1.39 | down |
| DW66_3378 | type 11 methyltransferase | -5.45 | down |
| DW66_3379 | hypothetical protein | -2.17 | down |
| DW66_3380 | 2Fe-2S iron-sulfur cluster binding domain-containing protein | 2.37 | up |
| DW66_3381 | aldehyde oxidase and xanthine dehydrogenase molybdopterin binding protein | 2.70 | up |
| DW66_3382 | gluconate 2-dehydrogenase | 1.86 | up |
| DW66_3385 | lysine exporter protein LysE/YggA | -1.34 | down |
| DW66_3388 | choline/carnitine/betaine transporter | -3.22 | down |
| DW66_3389 | 3-demethylubiquinone-9 3-methyltransferase | 1.71 | up |
| DW66_3390 | SH3 type 3 domain-containing protein | -2.65 | down |
| DW66_3391 | hypothetical protein | 7.47 | up |
| DW66_3392 | transcriptional regulator LysR | -2.49 | down |
| DW66_3393 | short-chain dehydrogenase/reductase SDR | -2.32 | down |
| DW66_3395 | transcriptional regulator LysR | -2.98 | down |
| DW66_3396 | N-acetyl-gamma-glutamyl-phosphate reductase | -4.65 | down |
| DW66_3397 | acetyltransferase | -1.17 | down |
| DW66_3400 | sulfonate ABC transporter ATP-binding protein | -1.53 | down |
| DW66_3401 | acyl-CoA dehydrogenase | -1.05 | down |
| DW66_3402 | alkylhydroperoxidase | -1.47 | down |
| DW66_3403 | transcriptional regulator AraC | -1.67 | down |
| DW66_3404 | transcriptional regulator | 2.69 | up |
| DW66_3405 | FAD-dependent pyridine nucleotide-disulfide oxidoreductase | 2.02 | up |
| DW66_3406 | cytosine/purines uracil thiamine allantoin permease | -2.31 | down |
| DW66_3408 | luciferase | 2.85 | up |
| DW66_3409 | hypothetical protein | 2.44 | up |
| DW66_3410 | alpha/beta hydrolase fold protein | 1.16 | up |
| DW66_3411 | aldehyde dehydrogenase | 1.10 | up |
| DW66_3412 | flavin reductase domain-containing protein | 2.53 | up |
| DW66_3414 | transcriptional regulator | -1.34 | down |
| DW66_3415 | NUDIX hydrolase | -2.18 | down |
| DW66_3416 | transposase | -1.19 | down |
| DW66_3417 | transposase | -2.37 | down |
| DW66_3418 | GAF sensor hybrid histidine kinase | -1.31 | down |
| DW66_3420 | lysine exporter protein LysE/YggA | 3.32 | up |
| DW66_3422 | cytosine/purines uracil thiamine allantoin permease | -3.50 | down |
| DW66_3423 | ammonium transporter | -1.06 | down |
| DW66_3424 | ferredoxin-dependent glutamate synthase | -2.58 | down |
| DW66_3425 | glutamate synthase subunit alpha | -3.05 | down |
| DW66_3426 | glutamine amidotransferase | -4.19 | down |
| DW66_3427 | glutamine synthetase | -3.22 | down |
| DW66_3431 | nicotinamidase | 3.46 | up |
| DW66_3432 | nicotinate phosphoribosyltransferase | 2.14 | up |
| DW66_3435 | membrane protein YeiH | -1.14 | down |
| DW66_3436 | Decarboxylase | 1.79 | up |
| DW66_3437 | diguanylate cyclase | -1.41 | down |
| DW66_3438 | hypothetical protein | 5.73 | up |
| DW66_3441 | catalase/peroxidase HPI | 6.16 | up |
| DW66_3442 | transcriptional regulator LysR | -1.70 | down |
| DW66_3445 | transposase | -1.34 | down |
| DW66_3446 | aldo/keto reductase | 1.62 | up |
| DW66_3449 | hypothetical protein | -2.97 | down |
| DW66_3451 | muconolactone delta-isomerase | -2.64 | down |
| DW66_3452 | muconate and chloromuconate cycloisomerase | -1.40 | down |
| DW66_3454 | D-serine dehydratase | -3.20 | down |
| DW66_3455 | transcriptional regulator | -2.39 | down |
| DW66_3456 | Omega-amino acid--pyruvate aminotransferase | -1.82 | down |
| DW66_3457 | extracellular solute-binding protein | -1.89 | down |
| DW66_3462 | Acetohydroxy acid synthase | -1.04 | down |
| DW66_3463 | acyl-CoA synthetase | -1.50 | down |
| DW66_3464 | acyl-CoA dehydrogenase domain-containing protein | -1.02 | down |
| DW66_3466 | amino acid permease-associated protein | -2.47 | down |
| DW66_3467 | multi-sensor hybrid histidine kinase | -3.98 | down |
| DW66_3468 | extracellular solute-binding protein | -6.02 | down |
| DW66_3469 | two component transcriptional regulator | -2.42 | down |
| DW66_3470 | transcriptional regulator TetR | -1.67 | down |
| DW66_3471 | enoyl-CoA hydratase/isomerase | -1.57 | down |
| DW66_3474 | ABC transporter ATP-binding protein | 1.64 | up |
| DW66_3478 | IcmF-related protein | -4.71 | down |
| DW66_3479 | penicillin-binding protein 2 | -2.71 | down |
| DW66_3480 | hypothetical protein | -10.13 | down |
| DW66_3484 | glycolate oxidase subunit GlcD | -4.36 | down |
| DW66_3485 | glycolate oxidase, FAD binding subunit | -3.41 | down |
| DW66_3486 | glycolate oxidase, iron-sulfur subunit | -3.22 | down |
| DW66_3487 | hypothetical protein | -1.85 | down |
| DW66_3488 | malate synthase G | -2.09 | down |
| DW66_3489 | hypothetical protein | -5.24 | down |
| DW66_3490 | hypothetical protein | -2.97 | down |
| DW66_3491 | transcriptional regulator | -1.08 | down |
| DW66_3492 | transporter protein | -2.49 | down |
| DW66_3493 | deaminase | -1.95 | down |
| DW66_3495 | transcriptional regulator AraC | -3.24 | down |
| DW66_3496 | *beta*-ketothiolase | 2.02 | up |
| DW66_3497 | 3-hydroxybutyryl-CoA dehydrogenase | 2.33 | up |
| DW66_3498 | transcriptional regulator TetR | 2.68 | up |
| DW66_3499 | membrane protein | -1.95 | down |
| DW66_3502 | MiaB-like tRNA modifying protein YliG | -3.83 | down |
| DW66_3506 | hypothetical protein | -13.46 | down |
| DW66_3507 | acetyltransferase | 1.79 | up |
| DW66_3512 | integral membrane sensor signal transduction histidine kinase | -5.41 | down |
| DW66_3513 | two component transcriptional regulator | -5.78 | down |
| DW66_3514 | outer membrane protein H1 | -4.78 | down |
| DW66_3515 | dienelactone hydrolase | 5.32 | up |
| DW66_3516 | 4'-phosphopantetheinyl transferase | -1.33 | down |
| DW66_3517 | integral membrane sensor signal transduction histidine kinase | -2.29 | down |
| DW66_3518 | two component transcriptional regulator | -2.32 | down |
| DW66_3519 | hypothetical protein | -1.74 | down |
| DW66_3521 | hypothetical protein | -2.69 | down |
| DW66_3522 | ribonucleotide-diphosphate reductase subunit beta | 1.47 | up |
| DW66_3528 | ISPpu14, transposase | -4.64 | down |
| DW66_3529 | transposase | -3.61 | down |
| DW66_3532 | hypothetical protein | -2.04 | down |
| DW66_3533 | hypothetical protein | -11.20 | down |
| DW66_3534 | N-ethylmaleimide reductase | -1.90 | down |
| DW66_3535 | hypothetical protein | -1.66 | down |
| DW66_3536 | oxidoreductase | -3.62 | down |
| DW66_3537 | MFS transporter | -3.25 | down |
| DW66_3538 | transcriptional regulator LysR | -2.33 | down |
| DW66_3539 | NAD binding domain protein | -1.20 | down |
| DW66_3540 | hypothetical protein | 1.79 | up |
| DW66_3541 | hypothetical protein | -3.30 | down |
| DW66_3548 | hypothetical protein | 2.43 | up |
| DW66_3549 | transcriptional regulator CRP/FNR | -1.82 | down |
| DW66_3550 | hypothetical protein | -1.34 | down |
| DW66_3551 | NADH:flavin oxidoreductase | -2.77 | down |
| DW66_3552 | AttM/AiiB protein | -3.49 | down |
| DW66_3554 | short-chain dehydrogenase | -2.06 | down |
| DW66_3557 | Transcriptional regulator | 1.63 | up |
| DW66_3559 | hypothetical protein | -2.25 | down |
| DW66_3563 | MFS_1 protein | 2.06 | up |
| DW66_3564 | transcriptional regulator IclR | 2.62 | up |
| DW66_3566 | transcriptional regulator LysR | 1.57 | up |
| DW66_3567 | RND transporter | -1.23 | down |
| DW66_3568 | hypothetical protein | -1.20 | down |
| DW66_3570 | hypothetical protein | 2.04 | up |
| DW66_3571 | hypothetical protein | -3.71 | down |
| DW66_3572 | PnpA | 9.02 | up |
| DW66_3573 | PnpB | 8.26 | up |
| DW66_3574 | PnpX2 | 4.35 | up |
| DW66_3575 | PnpX1 | 1.76 | up |
| DW66_3576 | hydroxyquinol 1,2-dioxygenase | 2.94 | up |
| DW66_3577 | PnpE | 5.01 | up |
| DW66_3578 | PnpD | 4.53 | up |
| DW66_3579 | PnpC2 | 5.15 | up |
| DW66_3580 | PnpC1 | 4.68 | up |
| DW66_3582 | hypothetical protein | -3.12 | down |
| DW66_3583 | Mobile element protein | -1.84 | down |
| DW66_3584 | TnpA protein | -1.20 | down |
| DW66_3590 | hypothetical protein | -9.92 | down |
| DW66_3594 | maleylacetate reductase | 2.70 | up |
| DW66_3595 | *gamma*-hydroxymuconic semialdehyde dehydrogenase | 2.37 | up |
| DW66_3596 | hydroquinone dioxygenase large subunit | 4.03 | up |
| DW66_3597 | hydroquinone dioxygenase small subunit | 4.71 | up |
| DW66_3598 | transcriptional regulator LysR | -1.13 | down |
| DW66_3600 | double-stranded beta helix domain protein-containing protein | -1.45 | down |
| DW66_3603 | resolvase protein | -1.32 | down |
| DW66_3605 | SAM-dependent methyltransferase | -1.52 | down |
| DW66_3606 | TnpA repressor protein | -1.65 | down |
| DW66_3607 | hypothetical protein | -1.42 | down |
| DW66_3608 | hypothetical protein | -1.10 | down |
| DW66_3609 | major facilitator transporter | -2.76 | down |
| DW66_3611 | transposase | 1.56 | up |
| DW66_3612 | hypothetical protein | -4.67 | down |
| DW66_3613 | pili assembly chaperone | 1.78 | up |
| DW66_3615 | DNA-directed DNA polymerase | -2.12 | down |
| DW66_3616 | transposase ISPs1 | -1.79 | down |
| DW66_3617 | DNA-binding protein | -1.11 | down |
| DW66_3618 | hypothetical protein | -1.26 | down |
| DW66_3619 | permease | -1.53 | down |
| DW66_3620 | permease | -3.43 | down |
| DW66_3621 | hypothetical protein | -2.43 | down |
| DW66_3622 | hypothetical protein | -1.89 | down |
| DW66_3623 | metallophosphoesterase protein | -2.03 | down |
| DW66_3627 | CTP:Inositol-1-phosphate cytidylyltransferase | -1.12 | down |
| DW66_3628 | hypothetical protein | -4.08 | down |
| DW66_3630 | hypothetical protein | -1.90 | down |
| DW66_3631 | integrase for In2 | -1.82 | down |
| DW66_3633 | TnpA protein | -1.57 | down |
| DW66_3634 | RNA polymerase sigma-70 factor | -4.87 | down |
| DW66_3636 | hypothetical protein | -5.18 | down |
| DW66_3637 | TnpA protein | -1.53 | down |
| DW66_3639 | integrase catalytic region | -1.29 | down |
| DW66_3643 | hypothetical protein | -1.68 | down |
| DW66_3644 | lipoprotein | -1.90 | down |
| DW66_3649 | oxidoreductase FAD-binding protein | 2.25 | up |
| DW66_3650 | glutathione S-transferase domain-containing protein | -3.74 | down |
| DW66_3651 | ISPpu14, transposase | -4.64 | down |
| DW66_3652 | transposase | -3.60 | down |
| DW66_3660 | precorrin 6A synthase | -3.19 | down |
| DW66_3661 | outer membrane porin | -5.14 | down |
| DW66_3662 | transcriptional regulator MvaT, P16 subunit | 2.52 | up |
| DW66_3663 | lactoylglutathione lyase | -1.49 | down |
| DW66_3664 | hypothetical protein | 1.84 | up |
| DW66_3666 | hypothetical protein | -2.13 | down |
| DW66_3667 | hypothetical protein | -2.34 | down |
| DW66_3668 | endolysin | -1.72 | down |
| DW66_3670 | phage repressor | -1.33 | down |
| DW66_3671 | hypothetical protein | 1.14 | up |
| DW66_3672 | alginate lyase 2 | 1.20 | up |
| DW66_3677 | peptidase | -3.26 | down |
| DW66_3678 | hypothetical protein | -1.68 | down |
| DW66_3680 | hypothetical protein | -1.66 | down |
| DW66_3681 | periplasmic solute binding protein | -1.53 | down |
| DW66_3683 | ABC transport protein | -2.24 | down |
| DW66_3684 | ABC transporter | -1.65 | down |
| DW66_3685 | lipoprotein | -2.03 | down |
| DW66_3686 | isochorismatase hydrolase | -1.49 | down |
| DW66_3687 | thioesterase | -1.69 | down |
| DW66_3688 | balhimycin biosynthetic protein MbtH | -1.93 | down |
| DW66_3689 | Ribosomal protein S3AE | -1.99 | down |
| DW66_3690 | formyltetrahydrofolate deformylase | -1.42 | down |
| DW66_3691 | methylenetetrahydrofolate dehydrogenase | -1.48 | down |
| DW66_3697 | alpha/beta hydrolase domain-containing protein | 1.88 | up |
| DW66_3698 | pirin domain-containing protein | 1.65 | up |
| DW66_3700 | bifunctional 3,4-dihydroxy-2-butanone 4-phosphate synthase/GTP cyclohydrolase II-like protein | 1.16 | up |
| DW66_3702 | extracellular solute-binding protein | 1.52 | up |
| DW66_3703 | binding-protein-dependent transport system inner membrane protein | 2.07 | up |
| DW66_3704 | binding-protein-dependent transport system inner membrane protein | 1.48 | up |
| DW66_3705 | Polyamine ABC transporter ATP-binding protein | 1.49 | up |
| DW66_3706 | OmpA/MotB domain-containing protein | -3.07 | down |
| DW66_3707 | integral membrane protein TerC | -1.78 | down |
| DW66_3708 | acyl-CoA dehydrogenase domain-containing protein | -1.17 | down |
| DW66_3709 | transcriptional regulator LysR | -1.55 | down |
| DW66_3710 | class V aminotransferase | 6.51 | up |
| DW66_3711 | RES domain-containing protein | -4.24 | down |
| DW66_3712 | hypothetical protein | -2.38 | down |
| DW66_3715 | transcriptional regulator AraC | -2.65 | down |
| DW66_3716 | lysine exporter protein LysE/YggA | -4.37 | down |
| DW66_3717 | sugar efflux transporter | -2.20 | down |
| DW66_3718 | hypothetical protein | -2.24 | down |
| DW66_3719 | alcohol dehydrogenase | -2.38 | down |
| DW66_3722 | hypothetical protein | -1.84 | down |
| DW66_3724 | alkylhydroperoxidase | 4.49 | up |
| DW66_3726 | serine/threonine transporter SstT | -1.20 | down |
| DW66_3727 | Virulence factor mviM | -1.13 | down |
| DW66_3728 | hypothetical protein | 2.06 | up |
| DW66_3729 | alkyl hydroperoxide reductase subunit F | 2.91 | up |
| DW66_3730 | alkyl hydroperoxide reductase subunit C | 5.18 | up |
| DW66_3733 | cytochrome c class I | -1.94 | down |
| DW66_3734 | cytochrome c-type protein | -2.26 | down |
| DW66_3735 | hypothetical protein | -3.11 | down |
| DW66_3740 | molybdate ABC transporter permease | -1.24 | down |
| DW66_3743 | carbon storage regulator | 1.10 | up |
| DW66_3748 | DoxX protein | 4.17 | up |
| DW66_3749 | hypothetical protein | 1.02 | up |
| DW66_3751 | hypothetical protein | -3.80 | down |
| DW66_3752 | transcriptional regulator LysR | -2.50 | down |
| DW66_3754 | retron-type reverse transcriptase | -12.31 | down |
| DW66_3756 | Phage protein | -1.61 | down |
| DW66_3757 | ISPpu15, transposase | -4.73 | down |
| DW66_3758 | ISPpu15, transposase | -1.35 | down |
| DW66_3759 | copper resistance protein B | -1.34 | down |
| DW66_3760 | hypothetical protein | 3.17 | up |
| DW66_3761 | copper-translocating P-type ATPase | 2.14 | up |
| DW66_3768 | sensor histidine kinase | -1.38 | down |
| DW66_3771 | transposase | 2.17 | up |
| DW66_3772 | aerotaxis receptor Aer | 3.62 | up |
| DW66_3773 | PAS/PAC sensor-containing diguanylate cyclase/phosphodiesterase | 3.38 | up |
| DW66_3774 | DNA-binding protein | -1.11 | down |
| DW66_3775 | hypothetical protein | -1.26 | down |
| DW66_3776 | permease | -1.53 | down |
| DW66_3777 | permease | -3.43 | down |
| DW66_3778 | hypothetical protein | -2.43 | down |
| DW66_3779 | hypothetical protein | -1.89 | down |
| DW66_3780 | metallophosphoesterase protein | -2.03 | down |
| DW66_3784 | CTP:Inositol-1-phosphate cytidylyltransferase | -1.12 | down |
| DW66_3785 | hypothetical protein | -4.08 | down |
| DW66_3787 | TniA transposase | -1.90 | down |
| DW66_3788 | integrase for In2 | -1.82 | down |
| DW66_3790 | TnpA protein | -1.57 | down |
| DW66_3791 | RNA polymerase sigma-71 factor | -4.87 | down |
| DW66_3793 | hypothetical protein | -5.18 | down |
| DW66_3794 | TnpA protein | -1.53 | down |
| DW66_3800 | hypothetical protein | -1.68 | down |
| DW66_3801 | lipoprotein | -1.90 | down |
| DW66_3806 | oxidoreductase FAD-binding protein | 2.25 | up |
| DW66_3807 | glutathione S-transferase domain-containing protein | -4.61 | down |
| DW66_3808 | ATPase AAA | -3.00 | down |
| DW66_3809 | ISPsy14, transposase | -2.78 | down |
| DW66_3810 | TnpT protein | 2.20 | up |
| DW66_3811 | Tn4652, cointegrate resolution protein S | 2.40 | up |
| DW66_3812 | hypothetical protein | -4.27 | down |
| DW66_3813 | hypothetical protein | -1.43 | down |
| DW66_3814 | hypothetical protein | -3.54 | down |
| DW66_3815 | hypothetical protein | -7.98 | down |
| DW66_3816 | transposase for Tn21 | -2.04 | down |
| DW66_3817 | resolvase for Tn21 | -1.28 | down |
| DW66_3818 | transposase | -2.98 | down |
| DW66_3820 | hypothetical protein | -1.80 | down |
| DW66_3824 | D12 class N6 adenine-specific DNA methyltransferase | -1.90 | down |
| DW66_3825 | hypothetical protein | -5.80 | down |
| DW66_3826 | terminase, large subunit | -2.25 | down |
| DW66_3827 | hypothetical protein | -6.24 | down |
| DW66_3828 | integrase | -3.61 | down |
| DW66_3830 | phosphoglycerate mutase | -1.71 | down |
| DW66_3834 | type 12 methyltransferase | -1.56 | down |
| DW66_3835 | phage-related lipoprotein | -2.08 | down |
| DW66_3839 | citrate transporter | -1.85 | down |
| DW66_3840 | diguanylate cyclase | -2.58 | down |
| DW66_3841 | membrane protein | -1.58 | down |
| DW66_3842 | transcriptional regulator LysR | -2.06 | down |
| DW66_3843 | isochorismatase hydrolase | -2.51 | down |
| DW66_3844 | hypothetical protein | -3.17 | down |
| DW66_3848 | xanthine permease | -3.24 | down |
| DW66_3854 | glutathione S-transferase domain-containing protein | 1.09 | up |
| DW66_3856 | seryl-tRNA synthetase | -1.28 | down |
| DW66_3857 | camphor resistance protein CrcB | -2.35 | down |
| DW66_3858 | recombination factor protein RarA | -3.17 | down |
| DW66_3859 | lipoprotein chaperone | -3.07 | down |
| DW66_3860 | cell division protein FtsK | -1.35 | down |
| DW66_3862 | leucyl/phenylalanyl-tRNA-protein transferase | -2.61 | down |
| DW66_3863 | arginyl-tRNA-protein transferase | 2.28 | up |
| DW66_3865 | ATP-dependent Clp protease ATP-binding protein ClpA | 2.00 | up |
| DW66_3867 | cold-shock protein | 2.53 | up |
| DW66_3868 | isocitrate dehydrogenase | 4.62 | up |
| DW66_3870 | NUDIX hydrolase | -1.40 | down |
| DW66_3873 | adenylosuccinate lyase | -1.83 | down |
| DW66_3875 | acetyltransferase | 1.05 | up |
| DW66_3876 | DNA topoisomerase III | -2.61 | down |
| DW66_3878 | alpha/beta hydrolase fold protein | 4.81 | up |
| DW66_3879 | hypothetical protein | -4.55 | down |
| DW66_3880 | NADH pyrophosphatase | -2.26 | down |
| DW66_3882 | sodium/proton antiporter | -2.89 | down |
| DW66_3883 | deaminase-reductase domain-containing protein | -1.74 | down |
| DW66_3885 | ribonuclease Z | -2.05 | down |
| DW66_3888 | phenylhydantoinase | 2.31 | up |
| DW66_3891 | transcriptional regulator TetR | -1.36 | down |
| DW66_3896 | hypothetical protein | 4.82 | up |
| DW66_3897 | DNA and RNA helicases | -2.45 | down |
| DW66_3898 | hypothetical protein | -2.52 | down |
| DW66_3899 | hypothetical protein | -5.22 | down |
| DW66_3900 | VgrG protein | -4.76 | down |
| DW66_3904 | maltooligosyl trehalose synthase | 1.39 | up |
| DW66_3905 | hypothetical protein | 2.01 | up |
| DW66_3906 | glycogen debranching protein GlgX | 2.21 | up |
| DW66_3908 | outer membrane autotransporter | -2.74 | down |
| DW66_3909 | glycogen branching enzyme | 2.87 | up |
| DW66_3910 | trehalose synthase | 2.32 | up |
| DW66_3911 | alpha amylase | 1.23 | up |
| DW66_3917 | *gamma*-carboxygeranoyl-CoA hydratase | 1.37 | up |
| DW66_3919 | transcriptional regulator XRE | -3.26 | down |
| DW66_3920 | transcriptional regulator XRE | -2.00 | down |
| DW66_3921 | hypothetical protein | 3.09 | up |
| DW66_3922 | *sigma*-70 region 4 type 2 | -4.50 | down |
| DW66_3923 | transporter protein | -1.36 | down |
| DW66_3924 | transcriptional regulator LysR | -2.00 | down |
| DW66_3925 | short-chain type regulator | -2.41 | down |
| DW66_3926 | MFS transporter | -2.96 | down |
| DW66_3927 | transketolase | -3.37 | down |
| DW66_3928 | transketolase, C-terminal subunit | -3.81 | down |
| DW66_3929 | phosphate ABC transporter ATP-binding protein | -3.33 | down |
| DW66_3930 | polar amino acid ABC transporter inner membrane subunit | -3.12 | down |
| DW66_3931 | polar amino acid ABC transporter inner membrane subunit | -4.69 | down |
| DW66_3932 | amino acid ABC transporter periplasmic amino acid-binding protein | -3.32 | down |
| DW66_3933 | transcriptional regulator | -1.75 | down |
| DW66_3935 | GAF and PAS/PAC sensor-containing diguanylate cyclase/phosphodiesterase | -1.27 | down |
| DW66_3937 | Pas/Pac sensor-containing chemotaxis sensory transducer | -2.97 | down |
| DW66_3938 | ImcF domain-containing protein | -2.89 | down |
| DW66_3939 | hypothetical protein | -5.78 | down |
| DW66_3941 | excinuclease ABC subunit C | -2.22 | down |
| DW66_3945 | uroporphyrin-III c-methyltransferase | 1.12 | up |
| DW66_3946 | phosphate transporter | 1.06 | up |
| DW66_3947 | glutathione S-transferase domain-containing protein | 1.07 | up |
| DW66_3948 | thioesterase protein | 1.09 | up |
| DW66_3949 | amino acid permease-associated protein | 1.27 | up |
| DW66_3951 | class III aminotransferase | 5.26 | up |
| DW66_3953 | hypothetical protein | -1.57 | down |
| DW66_3954 | elongation factor G | 5.14 | up |
| DW66_3955 | sulfate transporter | -4.06 | down |
| DW66_3956 | NolW domain-containing protein | 1.46 | up |
| DW66_3957 | hypothetical protein | -4.40 | down |
| DW66_3958 | isocitrate lyase | 4.58 | up |
| DW66_3960 | hypothetical protein | 2.54 | up |
| DW66_3962 | NADH dehydrogenase subunit A | 1.69 | up |
| DW66_3963 | NADH dehydrogenase subunit B | 2.92 | up |
| DW66_3964 | bifunctional NADH:ubiquinone oxidoreductase subunit C/D | 2.46 | up |
| DW66_3965 | NADH dehydrogenase subunit E | 2.94 | up |
| DW66_3966 | NADH dehydrogenase subunit F | 4.18 | up |
| DW66_3967 | NADH dehydrogenase subunit G | 2.71 | up |
| DW66_3968 | NADH:ubiquinone oxidoreductase subunit H | 2.59 | up |
| DW66_3969 | NADH dehydrogenase subunit I | 3.80 | up |
| DW66_3970 | NADH dehydrogenase subunit J | 2.93 | up |
| DW66_3971 | NADH dehydrogenase subunit K | 2.64 | up |
| DW66_3972 | NADH dehydrogenase subunit L | 2.28 | up |
| DW66_3973 | NADH:ubiquinone oxidoreductase subunit M | 1.97 | up |
| DW66_3974 | NADH dehydrogenase subunit N | 3.09 | up |
| DW66_3975 | hypothetical protein | 1.32 | up |
| DW66_3978 | hypothetical protein | -2.70 | down |
| DW66_3979 | transcriptional regulator | -2.76 | down |
| DW66_3980 | hypothetical protein | -3.47 | down |
| DW66_3981 | TonB-dependent receptor | -1.40 | down |
| DW66_3982 | NADPH-dependent FMN reductase | 1.35 | up |
| DW66_3984 | lysine decarboxylase | 2.67 | up |
| DW66_3985 | DNA polymerase III subunit epsilon | -1.78 | down |
| DW66_3989 | MltD domain-containing protein | -1.05 | down |
| DW66_3990 | extracellular solute-binding protein | -1.52 | down |
| DW66_3992 | binding-protein-dependent transport system inner membrane protein | -1.98 | down |
| DW66_3994 | peptide ABC transporter ATP-binding protein | 1.02 | up |
| DW66_3995 | hypothetical protein | 1.61 | up |
| DW66_3998 | aminotransferase, class III | -1.77 | down |
| DW66_3999 | transcriptional regulator LysR | -2.00 | down |
| DW66_4000 | hypothetical protein | -5.82 | down |
| DW66_4001 | hypothetical protein | -2.32 | down |
| DW66_4003 | osmosensitive K+ channel signal transduction histidine kinase | -1.52 | down |
| DW66_4004 | potassium-transporting, ATPase subunit C | -2.41 | down |
| DW66_4005 | potassium-transporting, ATPase subunit B | -3.13 | down |
| DW66_4006 | potassium-transporting ATPase subunit A | -2.76 | down |
| DW66_4007 | membrane protein | -1.91 | down |
| DW66_4008 | Type IIA topoisomerase (DNA gyrase/topo II, topoisomerase IV), A subunit | -1.34 | down |
| DW66_4009 | alpha/beta hydrolase fold protein | -1.36 | down |
| DW66_4013 | NAD(P)H-dependent glycerol-3-phosphate dehydrogenase | -1.02 | down |
| DW66_4014 | hypothetical protein | -2.12 | down |
| DW66_4015 | TonB-dependent receptor plug | -1.14 | down |
| DW66_4016 | hypothetical protein | -2.56 | down |
| DW66_4017 | integral membrane sensor hybrid histidine kinase | -1.06 | down |
| DW66_4020 | amidohydrolase 2 | -1.14 | down |
| DW66_4021 | pirin domain-containing protein | 1.17 | up |
| DW66_4022 | dienelactone hydrolase | 2.77 | up |
| DW66_4023 | heat shock protein 90 | 6.49 | up |
| DW66_4026 | hypothetical protein | 1.62 | up |
| DW66_4027 | hypothetical protein | 1.65 | up |
| DW66_4028 | branched-chain amino acid transport system II carrier protein | -1.87 | down |
| DW66_4031 | dihydrolipoamide dehydrogenase | 1.52 | up |
| DW66_4032 | dihydrolipoamide succinyltransferase | 1.32 | up |
| DW66_4033 | 2-oxoglutarate dehydrogenase E1 component | 1.19 | up |
| DW66_4034 | succinate dehydrogenase iron-sulfur subunit | 1.47 | up |
| DW66_4036 | succinate dehydrogenase, hydrophobic membrane anchor protein | -1.45 | down |
| DW66_4037 | succinate dehydrogenase, cytochrome b556 subunit | -1.78 | down |
| DW66_4039 | lipid-binding START domain-containing protein | -1.35 | down |
| DW66_4040 | proteinase inhibitor | -1.88 | down |
| DW66_4041 | Translation initiation factor 2 | -2.14 | down |
| DW66_4043 | OmpA/MotB domain-containing protein | -1.73 | down |
| DW66_4044 | lipoprotein | -1.90 | down |
| DW66_4045 | Extracellular solute-binding protein | -1.28 | down |
| DW66_4047 | electron transfer flavoprotein alpha/beta-subunit | -1.50 | down |
| DW66_4048 | electron transfer flavoprotein-ubiquinone oxidoreductase | -1.92 | down |
| DW66_4051 | RNA polymerase sigma factor | -3.30 | down |
| DW66_4052 | RND efflux transporter, MFP subunit | -2.73 | down |
| DW66_4054 | RND efflux system outer membrane lipoprotein | -1.70 | down |
| DW66_4055 | Pyoverdine biosynthesis related protein PvdP | -2.14 | down |
| DW66_4056 | peptidase M19 | -2.93 | down |
| DW66_4057 | class V aminotransferase | -1.91 | down |
| DW66_4058 | alpha/beta hydrolase domain-containing protein | -1.77 | down |
| DW66_4059 | transcription factor jumonji domain-containing protein | -2.34 | down |
| DW66_4060 | diaminobutyrate-2-oxoglutarate aminotransferase | -2.49 | down |
| DW66_4061 | integral membrane sensor signal transduction histidine kinase | -1.06 | down |
| DW66_4062 | two component transcriptional regulator | -1.03 | down |
| DW66_4063 | hypothetical protein | -1.45 | down |
| DW66_4065 | GAD-like domain-containing protein | -1.11 | down |
| DW66_4066 | MobA-like protein | -1.94 | down |
| DW66_4070 | aldehyde oxidase and xanthine dehydrogenase molybdopterin binding protein | -1.18 | down |
| DW66_4071 | protein-disulfide reductase | -1.32 | down |
| DW66_4074 | peptide synthase | -1.08 | down |
| DW66_4075 | extracytoplasmic-function sigma-70 factor | -6.33 | down |
| DW66_4076 | siderophore biosynthesis protein | -3.96 | down |
| DW66_4077 | extracellular solute-binding protein | -1.13 | down |
| DW66_4081 | cbb3-type cytochrome c oxidase subunit I | 2.29 | up |
| DW66_4082 | cbb3-type cytochrome c oxidase subunit II | 3.37 | up |
| DW66_4083 | cbb3-type cytochrome c oxidase, CcoQ subunit | 1.49 | up |
| DW66_4084 | cbb3-type cytochrome c oxidase subunit III | 2.93 | up |
| DW66_4085 | cbb3-type cytochrome c oxidase subunit I | 2.12 | up |
| DW66_4086 | cbb3-type cytochrome c oxidase subunit II | 4.11 | up |
| DW66_4087 | cbb3-type cytochrome c oxidase, CcoQ subunit | 4.28 | up |
| DW66_4088 | cbb3-type cytochrome c oxidase subunit III | 4.02 | up |
| DW66_4089 | cbb3-type cytochrome c oxidase, accessory protein CcoG | 2.44 | up |
| DW66_4090 | hypothetical protein | 1.63 | up |
| DW66_4094 | coproporphyrinogen III oxidase | 1.88 | up |
| DW66_4097 | recombination protein RecR | -2.75 | down |
| DW66_4098 | hypothetical protein | 1.25 | up |
| DW66_4099 | DNA polymerase III subunits gamma and tau | -1.33 | down |
| DW66_4101 | hypothetical protein | 1.15 | up |
| DW66_4102 | membrane protein | -3.11 | down |
| DW66_4103 | transcriptional regulator MerR | 1.40 | up |
| DW66_4104 | NAD-dependent DNA ligase LigA | -2.39 | down |
| DW66_4105 | hypothetical protein | -1.67 | down |
| DW66_4107 | chromosome segregation protein SMC | -1.37 | down |
| DW66_4108 | transcriptional regulator | -1.43 | down |
| DW66_4109 | xanthine dehydrogenase small subunit | 1.64 | up |
| DW66_4110 | xanthine dehydrogenase molybdopterin binding subunit | 3.82 | up |
| DW66_4111 | xanthine dehydrogenase accessory factor XdhC | 1.60 | up |
| DW66_4112 | guanine deaminase | 1.61 | up |
| DW66_4113 | aquaporin Z | 1.21 | up |
| DW66_4116 | transposase | -1.28 | down |
| DW66_4120 | ureidoglycolate hydrolase | 1.93 | up |
| DW66_4122 | xanthine/uracil permease | -3.06 | down |
| DW66_4124 | nucleoside-binding outer membrane protein-like protein | -2.46 | down |
| DW66_4126 | transcriptional regulator TetR | -1.62 | down |
| DW66_4127 | GlcG protein | -4.27 | down |
| DW66_4128 | hypothetical protein | -4.76 | down |
| DW66_4129 | glyoxylate carboligase | 1.16 | up |
| DW66_4132 | hydroxypyruvate reductase | -2.09 | down |
| DW66_4133 | pyruvate kinase | -2.20 | down |
| DW66_4136 | sulfate ABC transporter substrate-binding protein | -2.63 | down |
| DW66_4137 | Methyltransferase | -2.55 | down |
| DW66_4138 | Permease of the drug/metabolite transporter | -2.41 | down |
| DW66_4140 | cytosine/purines uracil thiamine allantoin permease | -2.52 | down |
| DW66_4142 | type I hydrophobic transmembrane region and ATP/GTP binding motif protein | -3.01 | down |
| DW66_4143 | hypothetical protein | -2.04 | down |
| DW66_4144 | hypothetical protein | -3.20 | down |
| DW66_4145 | FKBP-type peptidylprolyl isomerase | -1.96 | down |
| DW66_4147 | phenazine biosynthesis protein | -2.98 | down |
| DW66_4148 | D-isomer specific 2-hydroxyacid dehydrogenase NAD-binding protein | -3.21 | down |
| DW66_4149 | Cytochrome c heme lyase subunit CcmH | -1.31 | down |
| DW66_4151 | cytochrome c-type biogenesis protein CcmI | -2.47 | down |
| DW66_4152 | cytochrome C biogenesis protein | -2.90 | down |
| DW66_4153 | periplasmic protein thiol-disulfide oxidoreductase DsbE | -1.51 | down |
| DW66_4154 | cytochrome c-type biogenesis protein CcmF | -1.44 | down |
| DW66_4155 | cytochrome c-type biogenesis protein CcmE | -1.20 | down |
| DW66_4156 | heme exporter protein CcmD | -1.76 | down |
| DW66_4157 | heme exporter protein CcmC | -1.58 | down |
| DW66_4158 | heme exporter protein CcmB | -3.40 | down |
| DW66_4159 | cytochrome c biogenesis protein CcmA | -3.27 | down |
| DW66_4160 | hypothetical protein | -9.86 | down |
| DW66_4161 | hypothetical protein | -1.40 | down |
| DW66_4162 | hypothetical protein | -4.86 | down |
| DW66_4164 | hypothetical protein | -1.42 | down |
| DW66_4165 | transcriptional regulator AraC | -1.76 | down |
| DW66_4166 | hypothetical protein | -2.92 | down |
| DW66_4167 | hypothetical protein | -1.13 | down |
| DW66_4168 | O-succinylhomoserine sulfhydrylase | -1.38 | down |
| DW66_4169 | transporter protein | -1.81 | down |
| DW66_4170 | Pyrroloquinoline quinone (Coenzyme PQQ) biosynthesis protein C | -1.34 | down |
| DW66_4171 | acetyl-transferase | -2.10 | down |
| DW66_4172 | hypothetical protein | -1.38 | down |
| DW66_4173 | hypothetical protein | -2.00 | down |
| DW66_4174 | hypothetical protein | -3.82 | down |
| DW66_4175 | purine-binding chemotaxis protein CheW | -2.28 | down |
| DW66_4176 | CheW protein | -2.72 | down |
| DW66_4177 | cobyrinic acid a,c-diamide synthase | -2.69 | down |
| DW66_4178 | flagellar motor protein MotD | -2.15 | down |
| DW66_4179 | flagellar motor protein | -2.88 | down |
| DW66_4180 | chemotaxis-specific methylesterase | -2.02 | down |
| DW66_4181 | CheA signal transduction histidine kinase | -2.33 | down |
| DW66_4182 | chemotaxis phosphatase, CheZ | -2.56 | down |
| DW66_4183 | response regulator receiver protein | -1.52 | down |
| DW66_4184 | flagellar biosynthesis sigma factor | -1.28 | down |
| DW66_4186 | flagellar biosynthesis protein flhF | -1.18 | down |
| DW66_4187 | flagellar biosynthesis protein FlhA | -1.47 | down |
| DW66_4188 | flagellar biosynthesis protein FlhB | -4.41 | down |
| DW66_4189 | flagellar biosynthesis protein FliR | -4.19 | down |
| DW66_4190 | flagellar biosynthesis protein FliQ | -3.50 | down |
| DW66_4191 | flagellar biosynthesis protein FliP | -4.01 | down |
| DW66_4192 | flagellar biosynthesis protein FliO | -2.69 | down |
| DW66_4193 | flagellar motor switch protein | -1.81 | down |
| DW66_4194 | flagellar motor switch protein FliM | -2.04 | down |
| DW66_4195 | flagellar basal body-associated protein FliL | -3.08 | down |
| DW66_4196 | flagellar hook-length control protein | -1.80 | down |
| DW66_4198 | response regulator receiver protein | -1.04 | down |
| DW66_4200 | flagellar biosynthesis chaperone | -2.64 | down |
| DW66_4201 | flagellum-specific ATP synthase | -1.66 | down |
| DW66_4202 | flagellar assembly protein H | -2.73 | down |
| DW66_4204 | flagellar MS-ring protein | -2.22 | down |
| DW66_4205 | flagellar hook-basal body protein FliE | -1.89 | down |
| DW66_4207 | PAS/PAC sensor signal transduction histidine kinase | -1.58 | down |
| DW66_4209 | flagellin FliC | 1.45 | up |
| DW66_4210 | flagellin FlaG | 1.29 | up |
| DW66_4216 | group II intron-encoding maturase | 1.78 | up |
| DW66_4218 | flagellar hook-associated protein FlgL | 1.40 | up |
| DW66_4224 | flagellar basal body rod protein FlgF | -1.02 | down |
| DW66_4225 | hypothetical protein | -1.29 | down |
| DW66_4226 | flagellar hook protein FlgE | 1.40 | up |
| DW66_4229 | flagellar basal body rod protein FlgB | -1.95 | down |
| DW66_4230 | protein-glutamate O-methyltransferase | -1.06 | down |
| DW66_4232 | flagellar basal body P-ring biosynthesis protein FlgA | -3.26 | down |
| DW66_4237 | transcriptional regulator AsnC | -1.21 | down |
| DW66_4240 | branched-chain alpha-keto acid dehydrogenase subunit E2 | 1.71 | up |
| DW66_4241 | dihydrolipoyl dehydrogenase | 2.62 | up |
| DW66_4242 | PAS/PAC sensor-containing diguanylate cyclase | 3.14 | up |
| DW66_4245 | branched-chain amino acid transport | -1.00 | down |
| DW66_4246 | phosphonate metabolism protein/1,5-bisphosphokinase PhnN | -1.33 | down |
| DW66_4248 | magnesium transporter | -5.93 | down |
| DW66_4249 | carbon storage regulator | -2.39 | down |
| DW66_4250 | aspartate kinase | -2.14 | down |
| DW66_4252 | alanyl-tRNA ligase | -2.70 | down |
| DW66_4253 | hemolysin-like protein | -1.37 | down |
| DW66_4254 | succinylglutamate desuccinylase | 1.71 | up |
| DW66_4255 | GTPases | 1.66 | up |
| DW66_4256 | succinylarginine dihydrolase | 2.29 | up |
| DW66_4261 | hypothetical protein | -11.51 | down |
| DW66_4267 | hypothetical protein | -1.09 | down |
| DW66_4270 | PhhR | -2.37 | down |
| DW66_4271 | phenylalanine 4-monooxygenase | 1.99 | up |
| DW66_4272 | pterin-4-alpha-carbinolamine dehydratase | 3.02 | up |
| DW66_4273 | MFS transporter | -2.63 | down |
| DW66_4274 | FAD linked oxidase domain-containing protein | -2.10 | down |
| DW66_4275 | transcriptional regulator LysR | -1.84 | down |
| DW66_4277 | RNA-binding S4 domain-containing protein | -1.71 | down |
| DW66_4278 | hypothetical protein | -10.89 | down |
| DW66_4279 | segregation and condensation protein B | -1.70 | down |
| DW66_4280 | chromosome segregation and condensation protein ScpA | -1.14 | down |
| DW66_4283 | intracellular septation protein A | -1.62 | down |
| DW66_4285 | two component transcriptional regulator | -1.93 | down |
| DW66_4286 | hypothetical protein | -5.24 | down |
| DW66_4287 | integral membrane sensor signal transduction histidine kinase | -5.38 | down |
| DW66_4289 | potassium uptake protein TrkH | -3.29 | down |
| DW66_4291 | transcriptional regulator AraC | 2.05 | up |
| DW66_4292 | hypothetical protein | 2.32 | up |
| DW66_4293 | metallophosphoesterase | -5.20 | down |
| DW66_4294 | transcriptional regulator AraC | -2.69 | down |
| DW66_4295 | membrane protein | -2.07 | down |
| DW66_4296 | hypothetical protein | -1.79 | down |
| DW66_4298 | patatin | -2.98 | down |
| DW66_4302 | hypothetical protein | -1.28 | down |
| DW66_4304 | DTW domain-containing protein | -1.59 | down |
| DW66_4305 | methyl-accepting chemotaxis sensory transducer with Pas/Pac sensor | 2.39 | up |
| DW66_4306 | transcriptional regulator LysR | -2.37 | down |
| DW66_4307 | agmatinase | -3.16 | down |
| DW66_4309 | hypothetical protein | 4.74 | up |
| DW66_4310 | transcriptional regulator LysR | 1.32 | up |
| DW66_4311 | MFS precursor | -1.80 | down |
| DW66_4312 | Permease of the drug/metabolite transporter | -3.04 | down |
| DW66_4313 | hypothetical protein | -1.15 | down |
| DW66_4316 | DEAD/DEAH box helicase | -1.76 | down |
| DW66_4317 | hypothetical protein | -10.72 | down |
| DW66_4318 | mechanosensitive ion channel protein MscS | -2.53 | down |
| DW66_4319 | cytosine/purines uracil thiamine allantoin permease | -3.20 | down |
| DW66_4320 | carboxylate/amino acid/amine transporter | -2.77 | down |
| DW66_4321 | NAD(P)H dehydrogenase (quinone) | 1.88 | up |
| DW66_4323 | transcriptional regulator LysR | -1.61 | down |
| DW66_4327 | hypothetical protein | -1.55 | down |
| DW66_4330 | ATP-dependent helicase HrpA | -1.77 | down |
| DW66_4331 | glutamine synthetase | -1.16 | down |
| DW66_4332 | FAD dependent oxidoreductase | -1.10 | down |
| DW66_4333 | acyl coenzyme A synthetase 1 | -1.99 | down |
| DW66_4337 | RNA polymerase | 1.03 | up |
| DW66_4338 | activator of Hsp90 ATPase 1 | 1.28 | up |
| DW66_4339 | DGPFAETKE protein | 2.43 | up |
| DW66_4341 | Type IIA topoisomerase (DNA gyrase/topo II, topoisomerase IV), A subunit | 1.88 | up |
| DW66_4342 | acetyltransferase | -2.46 | down |
| DW66_4347 | RNA-binding S1 domain-containing protein | -1.30 | down |
| DW66_4349 | bifunctional isocitrate dehydrogenase kinase/phosphatase protein | 1.70 | up |
| DW66_4350 | DMT transporter permease | -2.08 | down |
| DW66_4351 | hypothetical protein | -1.89 | down |
| DW66_4352 | Permease of the drug/metabolite transporter | -2.35 | down |
| DW66_4353 | PvdO, pyoverdine responsive serine/threonine kinase (by OlgaV) | -3.66 | down |
| DW66_4354 | TonB-dependent siderophore receptor | -4.63 | down |
| DW66_4358 | amino acid adenylation domain-containing protein | -2.08 | down |
| DW66_4359 | hypothetical protein | -1.88 | down |
| DW66_4360 | hypothetical protein | -2.74 | down |
| DW66_4361 | pyoverdine biosynthesis regulatory protein | -4.49 | down |
| DW66_4362 | azurin | -2.95 | down |
| DW66_4363 | cyclic peptide transporter | -3.46 | down |
| DW66_4364 | aspartyl/asparaginyl beta-hydroxylase | -1.75 | down |
| DW66_4366 | hypothetical protein | -1.97 | down |
| DW66_4367 | hypothetical protein | -2.61 | down |
| DW66_4373 | transporter protein | -1.12 | down |
| DW66_4375 | hypothetical protein | -2.80 | down |
| DW66_4376 | glutamine amidotransferase | -1.82 | down |
| DW66_4377 | Permeases | -1.88 | down |
| DW66_4384 | hypothetical protein | -2.46 | down |
| DW66_4385 | hypothetical protein | -2.99 | down |
| DW66_4386 | nitroreductase | -1.03 | down |
| DW66_4387 | D-isomer specific 2-hydroxyacid dehydrogenase NAD-binding protein | -4.28 | down |
| DW66_4388 | Protein YcgL | -3.71 | down |
| DW66_4389 | ribonuclease D | -4.04 | down |
| DW66_4390 | sulfatase | -7.37 | down |
| DW66_4391 | 5-carboxymethyl-2-hydroxymuconate isomerase | -1.66 | down |
| DW66_4392 | hypothetical protein | 4.38 | up |
| DW66_4399 | molybdenum cofactor biosynthesis protein B | -1.91 | down |
| DW66_4400 | transcriptional regulator LysR | -1.13 | down |
| DW66_4401 | transcriptional regulator AraC | -2.02 | down |
| DW66_4402 | amino acid permease-associated protein | -14.52 | down |
| DW66_4403 | proline racemase | -13.23 | down |
| DW66_4404 | dihydrodipicolinate synthetase | -1.73 | down |
| DW66_4406 | FAD dependent oxidoreductase | -1.46 | down |
| DW66_4408 | transcriptional regulator ArsR | -1.85 | down |
| DW66_4409 | transcriptional regulator | 1.46 | up |
| DW66_4411 | malate:quinone oxidoreductase | -1.82 | down |
| DW66_4412 | proline racemase protein | -3.33 | down |
| DW66_4413 | outer membrane lipoprotein | -1.35 | down |
| DW66_4414 | potassium uptake protein TrkH | -2.17 | down |
| DW66_4415 | Na+/H+ antiporter NhaA | -1.00 | down |
| DW66_4417 | beta-lactamase domain-containing protein | -1.21 | down |
| DW66_4418 | lipoprotein | -1.36 | down |
| DW66_4420 | glycine cleavage system transcriptional repressor | -1.95 | down |
| DW66_4421 | alkyl hydroperoxide reductase/ thiol specific antioxidant/ Mal allergen | -2.19 | down |
| DW66_4422 | permease PerM | -3.22 | down |
| DW66_4423 | SirA protein | -1.50 | down |
| DW66_4426 | GTP-binding protein | -1.90 | down |
| DW66_4427 | amino acid ABC transporter permease | -5.50 | down |
| DW66_4428 | methyl-accepting chemotaxis protein | -4.75 | down |
| DW66_4430 | exsB protein | -2.22 | down |
| DW66_4431 | radical SAM domain-containing protein | -2.03 | down |
| DW66_4432 | tol-pal system protein YbgF | -2.16 | down |
| DW66_4433 | peptidoglycan-associated lipoprotein OprL | -1.40 | down |
| DW66_4434 | translocation protein TolB | -2.00 | down |
| DW66_4435 | TolA protein | -1.08 | down |
| DW66_4436 | biopolymer transport protein TolR | -2.24 | down |
| DW66_4437 | TolQ | -2.34 | down |
| DW66_4438 | 4-hydroxybenzoyl-CoA thioesterase | -1.26 | down |
| DW66_4441 | Holliday junction resolvase | -4.34 | down |
| DW66_4442 | hypothetical protein | -2.50 | down |
| DW66_4443 | aspartyl-tRNA synthetase | -1.96 | down |
| DW66_4445 | hypothetical protein | -1.15 | down |
| DW66_4446 | ferritin Dps protein | 1.61 | up |
| DW66_4448 | slyX protein | -1.12 | down |
| DW66_4449 | histidine triad (HIT) protein | -1.40 | down |
| DW66_4451 | prolyl-tRNA synthetase | -1.43 | down |
| DW66_4452 | lipoprotein | -1.65 | down |
| DW66_4454 | membrane protein | -1.95 | down |
| DW66_4457 | MiaB-like tRNA modifying protein YliG | -3.83 | down |
| DW66_4461 | hypothetical protein | -13.46 | down |
| DW66_4462 | acetyltransferase | 1.79 | up |
| DW66_4467 | integral membrane sensor signal transduction histidine kinase | -5.41 | down |
| DW66_4468 | two component transcriptional regulator | -5.78 | down |
| DW66_4469 | outer membrane protein H1 | -4.78 | down |
| DW66_4470 | dienelactone hydrolase | 5.32 | up |
| DW66_4471 | 4'-phosphopantetheinyl transferase | -1.33 | down |
| DW66_4472 | integral membrane sensor signal transduction histidine kinase | -2.29 | down |
| DW66_4473 | two component transcriptional regulator | -2.32 | down |
| DW66_4474 | hypothetical protein | -1.74 | down |
| DW66_4476 | hypothetical protein | -2.69 | down |
| DW66_4477 | ribonucleotide-diphosphate reductase subunit beta | 1.47 | up |
| DW66_4480 | hypothetical protein | -1.68 | down |
| DW66_4481 | lipoprotein | -1.90 | down |
| DW66_4486 | oxidoreductase FAD-binding protein | 2.25 | up |
| DW66_4487 | glutathione S-transferase domain-containing protein | -1.19 | down |
| DW66_4488 | metal-binding protein | -2.62 | down |
| DW66_4489 | asparaginase/glutaminase | -3.02 | down |
| DW66_4490 | hypothetical protein | -1.44 | down |
| DW66_4491 | nitrilase/cyanide hydratase and apolipoprotein N-acyltransferase | -3.15 | down |
| DW66_4492 | amine oxidase | -3.25 | down |
| DW66_4494 | acetolactate synthase | 1.44 | up |
| DW66_4496 | zinc-dependent hydrolase | -1.23 | down |
| DW66_4497 | diguanylate cyclase | 1.75 | up |
| DW66_4499 | PAS/PAC sensor-containing diguanylate cyclase/phosphodiesterase | -1.03 | down |
| DW66_4500 | lipoprotein | -1.20 | down |
| DW66_4501 | transcriptional regulator LysR | -2.67 | down |
| DW66_4503 | secretion protein HlyD | -1.75 | down |
| DW66_4504 | hypothetical protein | -2.16 | down |
| DW66_4505 | fusaric acid resistance protein region | -3.32 | down |
| DW66_4506 | hypothetical protein | 5.10 | up |
| DW66_4510 | ATP-dependent helicase HepA | 1.84 | up |
| DW66_4511 | diguanylate cyclase/phosphodiesterase | 3.23 | up |
| DW66_4512 | hypothetical protein | -3.67 | down |
| DW66_4517 | branched chain amino acid ABC transporter ATP-binding protein | 1.04 | up |
| DW66_4519 | FAD dependent oxidoreductase | -1.04 | down |
| DW66_4521 | 17 kDa surface antigen | -3.28 | down |
| DW66_4522 | 30S ribosomal protein S3 | 2.62 | up |
| DW66_4525 | beta-lactamase | -2.03 | down |
| DW66_4527 | ATP-dependent DNA helicase DinG | -2.97 | down |
| DW66_4528 | Phosphoribosylcarboxyaminoimidazole (NCAIR) mutase | -1.22 | down |
| DW66_4531 | OmpA/MotB domain-containing protein | 3.56 | up |
| DW66_4532 | OmpA/MotB domain-containing protein | 6.13 | up |
| DW66_4534 | hypothetical protein | 1.98 | up |
| DW66_4535 | water stress/hypersensitive response domain-containing protein | 1.28 | up |
| DW66_4537 | transporter protein | -1.97 | down |
| DW66_4539 | penicillin amidase | -1.79 | down |
| DW66_4540 | exonuclease | -2.36 | down |
| DW66_4541 | ATP-dependent DNA ligase | -1.58 | down |
| DW66_4542 | succinylglutamate desuccinylase/aspartoacylase | 1.06 | up |
| DW66_4543 | DEAD/DEAH box helicase | -2.45 | down |
| DW66_4544 | ICC-like protein phosphoesterase | -1.34 | down |
| DW66_4546 | deoxycytidine triphosphate deaminase | -1.72 | down |
| DW66_4547 | cold-shock domain-contain protein | -1.04 | down |
| DW66_4549 | methionyl-tRNA ligase | -1.35 | down |
| DW66_4552 | RnfABCDGE type electron transport complex subunit G | 1.03 | up |
| DW66_4556 | transcriptional regulator | -1.11 | down |
| DW66_4557 | hypothetical protein | -3.07 | down |
| DW66_4558 | argininosuccinate synthase | 1.87 | up |
| DW66_4562 | anti-oxidant AhpCTSA | 1.76 | up |
| DW66_4564 | bacterioferritin | 4.14 | up |
| DW66_4567 | ornithine carbamoyltransferase | -3.00 | down |
| DW66_4568 | ABC transporter ATP-binding protein | -2.90 | down |
| DW66_4570 | channel protein | -5.24 | down |
| DW66_4571 | glycerol kinase | -2.03 | down |
| DW66_4573 | glycerol-3-phosphate dehydrogenase | -1.46 | down |
| DW66_4574 | hypothetical protein | -3.49 | down |
| DW66_4576 | glutamate/aspartate ABC transporter permease | 2.68 | up |
| DW66_4577 | polar amino acid ABC transporter inner membrane subunit | 2.40 | up |
| DW66_4578 | glutamate-aspartate ABC transporter ATP-binding protein | 3.25 | up |
| DW66_4579 | integral membrane sensor signal transduction histidine kinase | -1.51 | down |
| DW66_4581 | GlpM protein | -1.14 | down |
| DW66_4582 | alpha/beta hydrolase | -1.65 | down |
| DW66_4583 | transcriptional regulator MetR | -2.39 | down |
| DW66_4584 | NUDIX hydrolase | -3.48 | down |
| DW66_4587 | amino acid permease-associated protein | -1.64 | down |
| DW66_4588 | membrane carboxypeptidase | 4.45 | up |
| DW66_4591 | Integral membrane protein | -1.96 | down |
| DW66_4593 | TonB-dependent receptor | -5.32 | down |
| DW66_4594 | carbohydrate-selective porin OprB | -1.77 | down |
| DW66_4595 | glucose dehydrogenase | 1.03 | up |
| DW66_4596 | ATP-dependent protease La | 3.86 | up |
| DW66_4598 | methyltransferase | -3.59 | down |
| DW66_4599 | methyltransferase | -3.82 | down |
| DW66_4600 | hypothetical protein | -2.40 | down |
| DW66_4601 | two component heavy metal response transcriptional regulator | -3.59 | down |
| DW66_4602 | heavy metal sensor signal transduction histidine kinase | -3.43 | down |
| DW66_4603 | pyridoxine 5'-phosphate synthase | -1.50 | down |
| DW66_4604 | DNA repair protein RecO | -6.40 | down |
| DW66_4605 | GTP-binding protein Era | -1.61 | down |
| DW66_4606 | ribonuclease III | -1.49 | down |
| DW66_4607 | signal peptidase I | -2.59 | down |
| DW66_4608 | GTP-binding protein LepA | -2.62 | down |
| DW66_4609 | hypothetical protein | -2.20 | down |
| DW66_4610 | protease Do | -1.85 | down |
| DW66_4611 | sigma E regulatory protein MucB/RseB | -2.21 | down |
| DW66_4612 | anti sigma-E protein, RseA | -1.63 | down |
| DW66_4613 | RNA polymerase sigma factor AlgU | -1.39 | down |
| DW66_4614 | L-aspartate oxidase | -2.83 | down |
| DW66_4615 | hypothetical protein | -2.07 | down |
| DW66_4618 | signal transduction protein | -2.01 | down |
| DW66_4621 | outer membrane porin | -4.05 | down |
| DW66_4622 | tricarboxylate transport protein TctC | -1.14 | down |
| DW66_4623 | tricarboxylate transport protein TctB | -1.31 | down |
| DW66_4625 | ammonia monooxygenase | -2.56 | down |
| DW66_4626 | uracil-DNA glycosylase | -3.14 | down |
| DW66_4627 | enoyl-CoA hydratase/isomerase | 3.51 | up |
| DW66_4631 | (R)-3-hydroxydecanoyl-ACP:CoA transacylase | 3.88 | up |
| DW66_4632 | sulfate transporter | -1.54 | down |
| DW66_4633 | hypothetical protein | -2.35 | down |
| DW66_4634 | Methyltransferase | -1.99 | down |
| DW66_4635 | hypothetical protein | -2.85 | down |
| DW66_4636 | glycoside hydrolase | -1.78 | down |
| DW66_4637 | integral membrane sensor signal transduction histidine kinase | -2.33 | down |
| DW66_4638 | two component sigma54 specific transcriptional regulator | -1.54 | down |
| DW66_4649 | NAD-dependent epimerase/dehydratase | 1.69 | up |
| DW66_4650 | *beta*-lactamase domain-containing protein | -1.10 | down |
| DW66_4651 | transcriptional regulator LysR | -1.32 | down |
| DW66_4652 | carboxyphosphonoenolpyruvate phosphonomutase | -1.51 | down |
| DW66_4653 | drug resistance transporter EmrB | -3.22 | down |
| DW66_4654 | transcriptional regulator TetR | -2.95 | down |
| DW66_4655 | RND efflux transporter, MFP subunit | -4.64 | down |
| DW66_4656 | hydrophobe/amphiphile efflux-1 | -3.92 | down |
| DW66_4657 | RND efflux system outer membrane lipoprotein | -1.83 | down |
| DW66_4658 | porin | -2.60 | down |
| DW66_4659 | hypothetical protein | 2.07 | up |
| DW66_4660 | 4-carboxymuconolactone decarboxylase | 5.29 | up |
| DW66_4661 | 3-oxoadipate enol-lactonase | 4.23 | up |
| DW66_4662 | 3-carboxy-cis,cis-muconate cycloisomerase | 3.57 | up |
| DW66_4663 | metabolite/H(+) symporter | 2.95 | up |
| DW66_4664 | beta-ketoadipyl-CoA thiolase | 5.66 | up |
| DW66_4665 | 3-oxoadipate:succinyl-CoA transferase subunit B | 5.07 | up |
| DW66_4666 | 3-oxoadipate:succinyl-CoA transferase subunit A | 2.80 | up |
| DW66_4670 | phosphate transporter | -3.09 | down |
| DW66_4673 | group 1 glycosyl transferase | 2.98 | up |
| DW66_4674 | hypothetical protein | 3.10 | up |
| DW66_4675 | hypothetical protein | 2.82 | up |
| DW66_4676 | formyltetrahydrofolate deformylase | -1.03 | down |
| DW66_4677 | Transcriptional regulator | -1.19 | down |
| DW66_4678 | exonuclease I | -1.61 | down |
| DW66_4679 | type IV pilus assembly PilZ | -1.13 | down |
| DW66_4682 | 2-polyprenylphenol hydroxylase and related flavodoxin oxidoreductases | -1.49 | down |
| DW66_4683 | hypothetical protein | -1.95 | down |
| DW66_4684 | Fe-S type hydro-lyase subunit alpha | -2.16 | down |
| DW66_4685 | nitrilase/cyanide hydratase and apolipoprotein N-acyltransferase | -3.28 | down |
| DW66_4686 | Histone acetyltransferase HPA2 and related acetyltransferases | -1.90 | down |
| DW66_4688 | glutamine amidotransferase | 1.94 | up |
| DW66_4689 | phospholipase | -2.77 | down |
| DW66_4690 | hypothetical protein | -2.57 | down |
| DW66_4691 | hypothetical protein | -1.09 | down |
| DW66_4692 | beta-lactamase | -2.74 | down |
| DW66_4694 | hemolysin | -2.51 | down |
| DW66_4696 | Acyl carrier protein phosphodiesterase | -1.26 | down |
| DW66_4697 | transcriptional regulator ArsR | -1.33 | down |
| DW66_4699 | hypothetical protein | -2.16 | down |
| DW66_4700 | NAD-dependent epimerase/dehydratase | -2.08 | down |
| DW66_4701 | chromosome replication initiation inhibitor protein | -1.77 | down |
| DW66_4702 | lysine exporter protein LysE/YggA | 2.60 | up |
| DW66_4704 | hydroxypyruvate isomerase | -3.92 | down |
| DW66_4705 | transcriptional regulator | -2.81 | down |
| DW66_4706 | glucose dehydrogenase | -3.04 | down |
| DW66_4707 | glucose dehydrogenase | -1.97 | down |
| DW66_4709 | Iron-regulated protein A precursor | -3.10 | down |
| DW66_4710 | thiol oxidoreductase with 2 cytochrome c heme-binding sites | -3.94 | down |
| DW66_4711 | lipoprotein | -2.34 | down |
| DW66_4712 | hypothetical protein | -2.05 | down |
| DW66_4713 | RND efflux transporter, MFP subunit | 1.28 | up |
| DW66_4715 | hypothetical protein | -1.09 | down |
| DW66_4716 | lipopolysaccharide kinase | -4.54 | down |
| DW66_4717 | type 11 methyltransferase | -3.77 | down |
| DW66_4718 | integral membrane sensor signal transduction histidine kinase | -2.36 | down |
| DW66_4719 | two component transcriptional regulator | -1.27 | down |
| DW66_4720 | PAP2 protein | -5.42 | down |
| DW66_4721 | chaperonin GroEL | 5.05 | up |
| DW66_4722 | co-chaperonin GroES | 5.58 | up |
| DW66_4724 | exclusion suppressor FxsA | 3.02 | up |
| DW66_4726 | Peptide chain release factor RF-3 | -3.06 | down |
| DW66_4727 | methylated-DNA-(protein)-cysteine S-methyltransferase DNA binding protein | 1.79 | up |
| DW66_4728 | AmpG-related permease | -1.01 | down |
| DW66_4729 | transporter protein | -3.03 | down |
| DW66_4730 | mechanosensitive ion channel protein MscS | -2.20 | down |
| DW66_4732 | 2-dehydropantoate 2-reductase | -1.09 | down |
| DW66_4734 | ATP-cobalamin adenosyltransferase | 2.06 | up |
| DW66_4735 | Mutator mutT protein (7,8-dihydro-8-oxoguanine-triphosphatase) | -1.17 | down |
| DW66_4739 | hypothetical protein | -2.22 | down |
| DW66_4740 | UDP-3-0-acyl N-acetylglucosamine deacetylase | 2.34 | up |
| DW66_4741 | cell division protein FtsZ | 2.07 | up |
| DW66_4744 | D-alanine--D-alanine ligase | 1.00 | up |
| DW66_4745 | UDP-N-acetylmuramate--L-alanine ligase | 1.51 | up |
| DW66_4746 | undecaprenyldiphospho-muramoylpentapeptide beta-N-acetylglucosaminyltransferase | 1.62 | up |
| DW66_4750 | UDP-N-acetylmuramoyl-tripeptide--D-alanyl-D- alanine ligase | 1.34 | up |
| DW66_4753 | cell division protein FtsL | -1.20 | down |
| DW66_4756 | ribosomal RNA small subunit methyltransferase I | -1.05 | down |
| DW66_4758 | endonuclease distantly related to archaeal Holliday junction resolvase | -1.56 | down |
| DW66_4759 | phosphoheptose isomerase | -1.74 | down |
| DW66_4760 | transport-associated protein | -1.18 | down |
| DW66_4761 | lipoprotein | -1.05 | down |
| DW66_4764 | ubiquinol-cytochrome c reductase, cytochrome c1 | 3.33 | up |
| DW66_4765 | cytochrome b/b6 domain-containing protein | 3.24 | up |
| DW66_4766 | ubiquinol-cytochrome c reductase, iron-sulfur subunit | 2.25 | up |
| DW66_4767 | 30S ribosomal protein S9 | -2.62 | down |
| DW66_4768 | 50S ribosomal protein L13 | -3.03 | down |
| DW66_4770 | transcriptional regulator AraC | -2.11 | down |
| DW66_4771 | ATPase AFG1 | -1.34 | down |
| DW66_4772 | tryptophanyl-tRNA synthetase | -2.04 | down |
| DW66_4773 | Alpha/beta hydrolase | -1.99 | down |
| DW66_4774 | hypothetical protein | 1.62 | up |
| DW66_4775 | methionine gamma-lyase | 1.71 | up |
| DW66_4776 | transcriptional regulator AsnC | -1.38 | down |
| DW66_4777 | bifunctional sulfate adenylyltransferase subunit 1/adenylylsulfate kinase protein | -1.41 | down |
| DW66_4778 | sulfate adenylyltransferase subunit 2 | -3.72 | down |
| DW66_4779 | hypothetical protein | -1.05 | down |
| DW66_4780 | 2-alkenal reductase | -1.66 | down |
| DW66_4784 | amino acid ABC transporter periplasmic amino acid-binding protein | 1.94 | up |
| DW66_4785 | hypothetical protein | -1.74 | down |
| DW66_4788 | molybdopterin biosynthesis protein MoaE | -1.44 | down |
| DW66_4790 | molybdenum cofactor biosynthesis protein MoaC | -1.22 | down |
| DW66_4791 | PhoH protein | 2.59 | up |
| DW66_4792 | polysaccharide deacetylase | -1.14 | down |
| DW66_4793 | hypothetical protein | -1.61 | down |
| DW66_4794 | GDP-mannose 6-dehydrogenase | -1.42 | down |
| DW66_4795 | glycosyl transferase Alg8 | -2.67 | down |
| DW66_4796 | type IV pilus assembly PilZ | -3.41 | down |
| DW66_4797 | Sel1 domain-containing protein | -2.84 | down |
| DW66_4798 | outer membrane protein AlgE | -1.26 | down |
| DW66_4799 | carbohydrate-binding and sugar hydrolysis protein | -3.14 | down |
| DW66_4800 | alginate biosynthesis protein AlgX | -2.25 | down |
| DW66_4801 | poly(beta-D-mannuronate) lyase | -2.07 | down |
| DW66_4802 | alginate o-acetyltransferase AlgI | -2.87 | down |
| DW66_4803 | protein AlgJ | -1.45 | down |
| DW66_4804 | alginate O-acetyltransferase | 1.86 | up |
| DW66_4805 | mannose-1-phosphate guanylyltransferase/mannose-6-phosphate isomerase | 2.51 | up |
| DW66_4808 | short-chain dehydrogenase/reductase | 2.73 | up |
| DW66_4809 | RND efflux system outer membrane lipoprotein | 1.85 | up |
| DW66_4810 | secretion protein HlyD | 2.25 | up |
| DW66_4811 | transporter protein | 1.24 | up |
| DW66_4812 | transcriptional regulator LysR | -1.98 | down |
| DW66_4814 | secretion protein HlyD | -1.88 | down |
| DW66_4815 | Na+-dependent transporters | -10.85 | down |
| DW66_4816 | fusaric acid resistance protein region | -2.72 | down |
| DW66_4817 | RND efflux system outer membrane lipoprotein | -3.25 | down |
| DW66_4819 | D-isomer specific 2-hydroxyacid dehydrogenase NAD-binding protein | -2.05 | down |
| DW66_4821 | hypothetical protein | -2.37 | down |
| DW66_4823 | TonB-dependent siderophore receptor | -3.31 | down |
| DW66_4824 | anti-FecI sigma factor FecR | -3.93 | down |
| DW66_4825 | RNA polymerase sigma-30 factor | -7.06 | down |
| DW66_4826 | hypothetical protein | -4.83 | down |
| DW66_4827 | PepSY-associated TM helix domain-containing protein | -2.76 | down |
| DW66_4828 | hypothetical protein | -4.07 | down |
| DW66_4829 | ribosomal subunit interface protein | 3.88 | up |
| DW66_4831 | phosphate-starvation-inducible E | 1.46 | up |
| DW66_4832 | hypothetical protein | 2.78 | up |
| DW66_4833 | leucine dehydrogenase | 2.53 | up |
| DW66_4835 | maleylacetoacetate isomerase | 2.94 | up |
| DW66_4836 | fumarylacetoacetase | 2.42 | up |
| DW66_4838 | transcriptional regulator IclR | -2.33 | down |
| DW66_4839 | short-chain dehydrogenase/reductase SDR | -1.73 | down |
| DW66_4840 | hypothetical protein | -10.10 | down |
| DW66_4841 | alpha/beta hydrolase fold protein | 1.33 | up |
| DW66_4846 | PAS/PAC sensor protein | -1.02 | down |
| DW66_4849 | membrane protein | -5.46 | down |
| DW66_4850 | short-chain dehydrogenase | -1.06 | down |
| DW66_4852 | HopJ type III effector protein | -1.50 | down |
| DW66_4855 | cytochrome B561 | 3.54 | up |
| DW66_4856 | signal peptide protein | 4.25 | up |
| DW66_4857 | acetyl-CoA acetyltransferase | 2.24 | up |
| DW66_4858 | 5- methyltetrahydropteroyltriglutamate/homocysteine S-methyltransferase | 2.72 | up |
| DW66_4859 | methylenetetrahydrofolate reductase | -1.80 | down |
| DW66_4862 | hypothetical protein | 3.01 | up |
| DW66_4863 | carbon starvation protein CstA | 1.93 | up |
| DW66_4864 | type IV pilus assembly PilZ | 1.63 | up |
| DW66_4865 | uracil-xanthine permease | 2.07 | up |
| DW66_4866 | DNA repair protein RadA | -1.87 | down |
| DW66_4867 | large conductance mechanosensitive channel protein MscL | 2.05 | up |
| DW66_4871 | Cyanide insensitive terminal oxidase, subunit III | 5.26 | up |
| DW66_4872 | cytochrome d ubiquinol oxidase subunit II | 3.92 | up |
| DW66_4873 | cytochrome bd ubiquinol oxidase subunit I | 3.37 | up |
| DW66_4874 | major facilitator superfamily protein | -2.91 | down |
| DW66_4877 | protocatechuate 3,4-dioxygenase subunit alpha | -1.56 | down |
| DW66_4878 | protocatechuate 3,4-dioxygenase subunit beta | -1.51 | down |
| DW66_4879 | metallopeptidase, zinc binding protein | 1.43 | up |
| DW66_4880 | methyl-accepting chemotaxis sensory transducer | -2.12 | down |
| DW66_4881 | *gamma*-glutamyltransferase | 1.12 | up |
| DW66_4883 | NAD(P)H dehydrogenase (quinone) | -1.73 | down |
| DW66_4885 | membrane protein | -5.27 | down |
| DW66_4887 | CDP-alcohol phosphatidyltransferase | -2.38 | down |
| DW66_4891 | phosphatidate cytidylyltransferase | 1.14 | up |
| DW66_4893 | transporter | 1.27 | up |
| DW66_4894 | hypothetical protein | -2.10 | down |
| DW66_4896 | methylmalonate-semialdehyde dehydrogenase | -1.18 | down |
| DW66_4897 | type VI secretion system effector | -3.06 | down |
| DW66_4898 | hypothetical protein | -1.26 | down |
| DW66_4899 | 3-hydroxyisobutyrate dehydrogenase | 6.75 | up |
| DW66_4900 | methylmalonate-semialdehyde dehydrogenase | 5.39 | up |
| DW66_4901 | transcriptional regulator LysR | -2.72 | down |
| DW66_4905 | exodeoxyribonuclease V subunit alpha | 2.23 | up |
| DW66_4906 | exodeoxyribonuclease V subunit beta | 1.52 | up |
| DW66_4907 | exodeoxyribonuclease V subunit gamma | 1.10 | up |
| DW66_4908 | sulfite oxidase, YedZ subunit | -1.79 | down |
| DW66_4909 | TMAO/DMSO reductase | -1.29 | down |
| DW66_4914 | hypothetical protein | -1.09 | down |
| DW66_4916 | penicillin-binding protein 1B | -2.09 | down |
| DW66_4917 | hypothetical protein | -3.05 | down |
| DW66_4919 | TfoX domain-containing protein | 3.17 | up |
| DW66_4920 | lipoprotein | -1.89 | down |
| DW66_4921 | hemin importer, ATP-binding subunit | -1.07 | down |
| DW66_4922 | transport system permease | -2.11 | down |
| DW66_4923 | periplasmic binding protein | -3.22 | down |
| DW66_4924 | Rieske (2Fe-2S) domain-containing protein | -1.60 | down |
| DW66_4925 | sugar fermentation stimulation protein A | -2.08 | down |
| DW66_4926 | Valine--pyruvate aminotransferase | -1.63 | down |
| DW66_4928 | glutamyl-Q tRNA(Asp) synthetase | -2.16 | down |
| DW66_4935 | 3-methyl-2-oxobutanoate hydroxymethyltransferase | -1.55 | down |
| DW66_4936 | pantoate--beta-alanine ligase | -2.51 | down |
| DW66_4937 | flagellar motor switch protein FliN | -4.40 | down |
| DW66_4939 | acetyl-CoA synthetase | 1.26 | up |
| DW66_4945 | transport-associated protein | 1.30 | up |
| DW66_4946 | polynucleotide phosphorylase/polyadenylase | -2.44 | down |
| DW66_4948 | tRNA pseudouridine synthase B | -1.55 | down |
| DW66_4949 | ribosome-binding factor A | -3.69 | down |
| DW66_4950 | translation initiation factor IF-2 | -2.72 | down |
| DW66_4951 | transcription elongation factor NusA | -2.73 | down |
| DW66_4952 | ribosome maturation factor rimP | -2.26 | down |
| DW66_4953 | triosephosphate isomerase | -3.40 | down |
| DW66_4956 | ATP-dependent metalloprotease FtsH | 1.18 | up |
| DW66_4957 | 23S rRNA methyltransferase J | 1.28 | up |
| DW66_4958 | hypothetical protein | 1.20 | up |
| DW66_4959 | Permeases | -1.82 | down |
| DW66_4960 | transcription elongation factor GreA | -1.89 | down |
| DW66_4961 | carbamoyl phosphate synthase, large subunit | -1.02 | down |
| DW66_4963 | dihydrodipicolinate reductase | 1.36 | up |
| DW66_4964 | chaperone protein DnaJ | 1.52 | up |
| DW66_4965 | molecular chaperone DnaK | 5.61 | up |
| DW66_4966 | heat shock protein GrpE | 5.27 | up |
| DW66_4972 | transcriptional regulator | 1.58 | up |
| DW66_4973 | L-lactate transport | 4.01 | up |
| DW66_4974 | L-lactate dehydrogenase | 3.37 | up |
| DW66_4981 | transporter protein | -1.16 | down |
| DW66_4983 | amino acid permease-associated protein | 1.31 | up |
| DW66_4984 | glucarate dehydratase | -3.06 | down |
| DW66_4985 | transporter protein | -3.70 | down |
| DW66_4987 | alcohol dehydrogenase | 1.36 | up |
| DW66_4988 | hydrolase | 1.41 | up |
| DW66_4991 | hypothetical protein | -1.62 | down |
| DW66_4992 | phosphohistidine phosphatase, SixA | -4.81 | down |
| DW66_4993 | histone deacetylase protein | -1.84 | down |
| DW66_4994 | NAD(FAD)-utilizing dehydrogenases | -2.75 | down |
| DW66_4995 | DEAD/DEAH box helicase | -2.92 | down |
| DW66_4996 | Permease of the drug/metabolite transporter | -2.38 | down |
| DW66_4998 | DNA polymerase III subunit epsilon | -1.91 | down |
| DW66_5001 | hypothetical protein | -1.69 | down |
| DW66_5002 | hypothetical protein | -1.56 | down |
| DW66_5003 | ATP-dependent helicase HrpB | -2.21 | down |
| DW66_5005 | transporter | -1.02 | down |
| DW66_5006 | hypothetical protein | -3.10 | down |
| DW66_5007 | transcriptional regulator AsnC | 1.05 | up |
| DW66_5011 | acyl-CoA dehydrogenase domain-containing protein | 1.30 | up |
| DW66_5012 | hypothetical protein | -2.62 | down |
| DW66_5013 | hypothetical protein | -2.51 | down |
| DW66_5014 | integral membrane sensor signal transduction histidine kinase | -1.40 | down |
| DW66_5015 | phosphomethylpyrimidine kinase | -1.34 | down |
| DW66_5016 | thiamine-phosphate pyrophosphorylase | -1.76 | down |
| DW66_5019 | hypothetical protein | -1.36 | down |
| DW66_5023 | apolipoprotein N-acyltransferase | -1.89 | down |
| DW66_5025 | hypothetical protein | 6.22 | up |
| DW66_5026 | leucyl-tRNA synthetase | -1.46 | down |
| DW66_5028 | DNA polymerase III subunit delta | -2.30 | down |
| DW66_5029 | D-isomer specific 2-hydroxyacid dehydrogenase, N | -1.64 | down |
| DW66_5033 | lipoyltransferase | -1.20 | down |
| DW66_5036 | rare lipoprotein A | -1.00 | down |
| DW66_5037 | lytic murein transglycosylase B | -2.30 | down |
| DW66_5038 | rod shape-determining protein RodA | -3.80 | down |
| DW66_5039 | penicillin-binding protein 2 | -3.39 | down |
| DW66_5040 | LSU m3Psi1915 methyltransferase RlmH | -2.26 | down |
| DW66_5041 | iojap protein | -1.88 | down |
| DW66_5043 | gamma-glutamyl phosphate reductase | -1.08 | down |
| DW66_5044 | 3-methyladenine DNA glycosylase | -5.17 | down |
| DW66_5045 | N-acetyltransferase GCN5 | -5.16 | down |
| DW66_5048 | LrgA protein | -1.44 | down |
| DW66_5050 | 50S ribosomal protein L11 methyltransferase | -1.60 | down |
| DW66_5051 | CDGSH-type zinc finger protein | -1.66 | down |
| DW66_5053 | DNA-binding protein Fis | -1.25 | down |
| DW66_5054 | bifunctional phosphoribosylaminoimidazolecarboxamide formyltransferase/IMP cyclohydrolase | -1.81 | down |
| DW66_5055 | hypothetical protein | -2.32 | down |
| DW66_5058 | multiple antibiotic resistance (MarC)-like protein | -2.58 | down |
| DW66_5059 | hypothetical protein | -3.66 | down |
| DW66_5060 | hypothetical protein | -2.75 | down |
| DW66_5061 | precorrin-3B C(17)-methyltransferase | -1.35 | down |
| DW66_5062 | precorrin-2 C20-methyltransferase | -1.45 | down |
| DW66_5064 | precorrin-3B synthase | -1.71 | down |
| DW66_5065 | hypothetical protein | -1.37 | down |
| DW66_5066 | precorrin-6y C5,15-methyltransferase (decarboxylating) CbiE subunit | -3.14 | down |
| DW66_5067 | cobalt-precorrin-6A synthase | -2.90 | down |
| DW66_5068 | cobalt-precorrin-6x reductase | -3.02 | down |
| DW66_5069 | activity regulator of membrane protease YbbK | -1.99 | down |
| DW66_5071 | IS111A/IS1328/IS1533, transposase | 1.27 | up |
| DW66_5074 | multicopper oxidases | -1.75 | down |
| DW66_5077 | D-alanine/D-serine/glycine permease | -1.68 | down |
| DW66_5078 | urea ABC transporter urea binding protein | -2.48 | down |
| DW66_5079 | urea ABC transporter permease UrtB | -5.16 | down |
| DW66_5080 | urea ABC transporter permease UrtC | -4.39 | down |
| DW66_5081 | branched-chain amino acid ABC transporter ATP-binding protein | -2.67 | down |
| DW66_5082 | urea ABC transporter ATP-binding protein | -2.13 | down |
| DW66_5083 | branched chain amino acid ABC transporter ATP-binding protein | 1.27 | up |
| DW66_5084 | Chaperone-modulator protein CbpM | 1.42 | up |
| DW66_5085 | chaperone DnaJ domain-containing protein | 1.35 | up |
| DW66_5086 | molecular chaperone-like protein | -3.72 | down |
| DW66_5088 | PsiF repeat-containing protein | 2.76 | up |
| DW66_5089 | transcriptional regulator AraC | -1.27 | down |
| DW66_5091 | alpha/beta hydrolase fold protein | -1.95 | down |
| DW66_5093 | ferritin Dps protein | 1.01 | up |
| DW66_5096 | transcriptional regulator TetR | -2.76 | down |
| DW66_5097 | N-acylglucosamine 2-epimerase | -1.13 | down |
| DW66_5098 | short-chain dehydrogenase/reductase SDR | -1.18 | down |
| DW66_5099 | Branched chain amino acid ABC transporter ATP-binding protein | 1.61 | up |
| DW66_5100 | branched chain amino acid ABC transporter ATP-binding protein | 1.58 | up |
| DW66_5103 | extracellular ligand-binding receptor | 3.60 | up |
| DW66_5104 | nicotinate phosphoribosyltransferase | -1.20 | down |
| DW66_5106 | azurin | 3.17 | up |
| DW66_5107 | Lysine decarboxylase | -1.20 | down |
| DW66_5108 | oxidoreductase | -2.75 | down |
| DW66_5109 | replicative DNA helicase | -2.34 | down |
| DW66_5110 | 50S ribosomal protein L9 | -2.20 | down |
| DW66_5111 | hypothetical protein | -2.72 | down |
| DW66_5112 | 30S ribosomal protein S18 | -1.93 | down |
| DW66_5113 | 30S ribosomal protein S6 | -1.83 | down |
| DW66_5116 | hypothetical protein | -1.83 | down |
| DW66_5117 | iron ABC transporter periplasmic iron-binding protein | -1.71 | down |
| DW66_5118 | binding-protein-dependent transport system inner membrane protein | -2.30 | down |
| DW66_5119 | integral membrane protein | -2.91 | down |
| DW66_5120 | type VI secretion system effector | -3.03 | down |
| DW66_5122 | adenylosuccinate synthetase | -1.24 | down |
| DW66_5123 | ATP phosphoribosyltransferase regulatory subunit | -2.97 | down |
| DW66_5127 | RNA-binding protein Hfq | 1.55 | up |
| DW66_5133 | iron-sulfur cluster binding protein | -1.57 | down |
| DW66_5134 | membrane protein | -3.09 | down |
| DW66_5136 | ribosome-associated GTPase | -1.22 | down |
| DW66_5137 | flagellar motor protein MotB | -2.02 | down |
| DW66_5138 | flagellar motor protein MotA | -1.21 | down |
| DW66_5139 | hypothetical protein | -1.18 | down |
| DW66_5143 | phosphoserine phosphatase SerB | -2.54 | down |
| DW66_5144 | HAMP domain-containing protein | -1.57 | down |
| DW66_5146 | DNA topoisomerase IV subunit A | -1.27 | down |
| DW66_5147 | Transporter | -2.45 | down |
| DW66_5148 | hypothetical protein | -1.86 | down |
| DW66_5150 | hypothetical protein | -2.01 | down |
| DW66_5151 | Icc protein | -2.68 | down |
| DW66_5154 | lipoprotein | -1.15 | down |
| DW66_5155 | hydroxymethylpyrimidine transporter CytX | -2.42 | down |
| DW66_5156 | phosphomethylpyrimidine synthase ThiC | -2.17 | down |
| DW66_5157 | type I secretion outer membrane protein TolC | 1.12 | up |
| DW66_5158 | IS111A/IS1328/IS1533, transposase | 1.27 | up |
| DW66_5159 | 3-deoxy-D-manno-octulosonic-acid transferase | -2.44 | down |
| DW66_5160 | transcriptional regulator LysR | -2.56 | down |
| DW66_5161 | small multidrug resistance protein | -3.13 | down |
| DW66_5167 | O-antigen polymerase | -1.79 | down |
| DW66_5169 | group 1 glycosyl transferase | 1.28 | up |
| DW66_5171 | Glycosyltransferase | -3.10 | down |
| DW66_5172 | LmbE protein | -2.15 | down |
| DW66_5173 | Mig-14 protein | -2.98 | down |
| DW66_5174 | group 1 glycosyl transferase | -1.79 | down |
| DW66_5175 | carbamoyltransferase | -1.44 | down |
| DW66_5176 | hypothetical protein | -1.38 | down |
| DW66_5178 | trifunctional transcriptional regulator/proline dehydrogenase/pyrroline-5-carboxylate dehydrogenase | 2.62 | up |
| DW66_5181 | peptidase U62 modulator of DNA gyrase | 1.27 | up |
| DW66_5182 | TldE/PmbA protein | 1.88 | up |
| DW66_5184 | ATP-dependent RNA helicase DbpA | -2.25 | down |
| DW66_5185 | hypothetical protein | -2.62 | down |
| DW66_5186 | integral membrane protein YccS | -1.32 | down |
| DW66_5187 | amino acid ABC transporter periplasmic amino acid-binding protein | -1.31 | down |
| DW66_5188 | acetyltransferase | -1.54 | down |
| DW66_5191 | response regulator receiver modulated diguanylate cyclase/phosphodiesterase with PAS/PAC sensor(s) | 2.34 | up |
| DW66_5194 | hypothetical protein | 1.17 | up |
| DW66_5195 | phosphoglycerate kinase | -1.89 | down |
| DW66_5196 | D-erythrose-4-phosphate dehydrogenase | -2.01 | down |
| DW66_5197 | transketolase | -1.53 | down |
| DW66_5198 | transcriptional regulator ArsR | 1.83 | up |
| DW66_5199 | S-adenosylmethionine synthetase | 2.67 | up |
| DW66_5200 | NAD-dependent DNA ligase LigB | -1.86 | down |
| DW66_5202 | cytochrome c class II | -2.43 | down |
| DW66_5203 | hypothetical protein | -3.09 | down |
| DW66_5204 | MltA domain-containing protein | -1.93 | down |
| DW66_5205 | membrane protein | -1.58 | down |
| DW66_5208 | thioesterase protein | 1.10 | up |
| DW66_5212 | extracellular solute-binding protein | -2.70 | down |
| DW66_5213 | hypothetical protein | -3.15 | down |
| DW66_5214 | DEAD/DEAH box helicase | -2.19 | down |
| DW66_5215 | Protein yceI precursor | 1.90 | up |
| DW66_5217 | amine oxidase | -2.00 | down |
| DW66_5218 | hypothetical protein | -7.72 | down |
| DW66_5219 | adenosylmethionine-8-amino-7-oxononanoate transaminase | -1.46 | down |
| DW66_5220 | 16S ribosomal RNA methyltransferase RsmE | -1.29 | down |
| DW66_5221 | hemolysin III channel protein | -2.56 | down |
| DW66_5224 | methyl-accepting chemotaxis sensory transducer | -2.15 | down |
| DW66_5225 | CheW protein | -1.52 | down |
| DW66_5228 | glutathione synthetase | -1.77 | down |
| DW66_5229 | TonB protein | -1.73 | down |
| DW66_5230 | hypothetical protein | -1.44 | down |
| DW66_5231 | Holliday junction resolvase-like protein | -2.28 | down |
| DW66_5232 | bifunctional protein pyrR | -2.13 | down |
| DW66_5233 | aspartate carbamoyltransferase catalytic subunit | -1.44 | down |
| DW66_5235 | ATP-dependent protease peptidase subunit | 5.75 | up |
| DW66_5236 | ATP-dependent protease, ATP-binding subunit HslU | 5.92 | up |
| DW66_5237 | hypothetical protein | 1.64 | up |
| DW66_5238 | poly(R)-hydroxyalkanoic acid synthase, class II | -2.27 | down |
| DW66_5239 | poly(3-hydroxyalkanoate) depolymerase | -1.24 | down |
| DW66_5243 | poly(hydroxyalcanoate) granule associated protein GA1 | -1.58 | down |
| DW66_5244 | hypothetical protein | -1.05 | down |
| DW66_5246 | sterol-binding domain-containing protein | -1.26 | down |
| DW66_5247 | 2-polyprenylphenol 6-hydroxylase | -1.49 | down |
| DW66_5248 | phosphoribosyl-AMP cyclohydrolase | 1.27 | up |
| DW66_5250 | twin arginine translocase protein A | -2.66 | down |
| DW66_5251 | sec-independent translocase | -3.80 | down |
| DW66_5252 | sec-independent protein translocase, TatC subunit | -3.25 | down |
| DW66_5253 | 16S ribosomal RNA methyltransferase RsmE | -3.54 | down |
| DW66_5255 | methyl-accepting chemotaxis sensory transducer | -4.07 | down |
| DW66_5256 | amino acid ABC transporter ATP-binding protein | -3.18 | down |
| DW66_5257 | polar amino acid ABC transporter inner membrane subunit | -2.34 | down |
| DW66_5258 | amino acid ABC transporter periplasmic amino acid-binding protein | -1.00 | down |
| DW66_5260 | glucan biosynthesis protein G | -1.86 | down |
| DW66_5263 | hypothetical protein | -3.90 | down |
| DW66_5264 | hypothetical protein | -4.87 | down |
| DW66_5265 | hypothetical protein | -1.77 | down |
| DW66_5268 | amino acid ABC transporter permease | -2.32 | down |
| DW66_5269 | histidine ammonia-lyase | 1.08 | up |
| DW66_5270 | urocanate hydratase | 2.29 | up |
| DW66_5271 | hypothetical protein | -2.09 | down |
| DW66_5272 | histidine utilization repressor | -1.48 | down |
| DW66_5273 | N-formimino-L-glutamate deiminase | 2.64 | up |
| DW66_5274 | lipocalin | -1.48 | down |
| DW66_5279 | Fe-S-oxidoreductase | -3.13 | down |
| DW66_5280 | PhoP/Q-regulated protein PqaA | -1.41 | down |
| DW66_5281 | GTP-binding protein TypA | -1.71 | down |
| DW66_5282 | thiamine biosynthesis protein ThiI | -3.07 | down |
| DW66_5284 | chorismate mutase | -11.47 | down |
| DW66_5288 | RNA methyltransferase | -1.56 | down |
| DW66_5289 | IS111A/IS1328/IS1533, transposase | 1.27 | up |
| DW66_5290 | preprotein translocase subunit SecB | -2.13 | down |
| DW66_5291 | glutaredoxin 3 | -1.76 | down |
| DW66_5295 | carboxyl-terminal protease | -1.32 | down |
| DW66_5296 | divergent polysaccharide deacetylase | -3.17 | down |
| DW66_5297 | hypothetical protein | -2.08 | down |
| DW66_5298 | choline/carnitine/betaine transporter | -2.14 | down |
| DW66_5299 | transcriptional regulator BetI | -2.81 | down |
| DW66_5300 | betaine aldehyde dehydrogenase | -1.30 | down |
| DW66_5302 | potassium/proton antiporter | -1.49 | down |
| DW66_5303 | potassium efflux protein KefA | -1.07 | down |
| DW66_5307 | transcriptional regulator LysR | -2.35 | down |
| DW66_5308 | transporter protein | 1.00 | up |
| DW66_5309 | hypothetical protein | -2.92 | down |
| DW66_5314 | sporulation domain-containing protein | -1.81 | down |
| DW66_5315 | 3-dehydroquinate synthase | -1.86 | down |
| DW66_5317 | type IV pilus secretin PilQ | -2.79 | down |
| DW66_5318 | type IV pili biogenesis protein PilP | -4.55 | down |
| DW66_5319 | fimbrial assembly protein | -2.37 | down |
| DW66_5321 | penicillin-binding protein 1A | -1.41 | down |
| DW66_5322 | malate dehydrogenase | -1.85 | down |
| DW66_5323 | nuclease | -1.60 | down |
| DW66_5325 | primosome assembly protein PriA | -2.49 | down |
| DW66_5326 | arginyl-tRNA synthetase | -1.59 | down |
| DW66_5327 | sporulation domain-containing protein | -1.08 | down |
| DW66_5329 | NLP/P60 protein | -1.25 | down |
| DW66_5330 | twitching motility protein | -1.38 | down |
| DW66_5333 | Integral membrane protein YggT | 1.60 | up |
| DW66_5334 | homoserine O-acetyltransferase | -1.91 | down |
| DW66_5336 | hypothetical protein | 2.61 | up |
| DW66_5337 | dITP/XTP pyrophosphatase | 3.00 | up |
| DW66_5338 | coproporphyrinogen III oxidase | 1.16 | up |
| DW66_5339 | Phosphotransferase system IIC components, glucose/maltose/N-acetylglucosamine-specific | -2.89 | down |
| DW66_5340 | tRNA (guanine-N(7)-)-methyltransferase | -2.15 | down |
| DW66_5341 | thiazole synthase | -2.56 | down |
| DW66_5342 | sulfur carrier protein ThiS | -1.98 | down |
| DW66_5343 | hypothetical protein | -2.17 | down |
| DW66_5344 | peptidoglycan transglycosylase | -1.81 | down |
| DW66_5347 | cell division ATP-binding protein FtsE | -1.36 | down |
| DW66_5348 | signal recognition particle-docking protein FtsY | -2.00 | down |
| DW66_5349 | hypothetical protein | -1.58 | down |
| DW66_5350 | peptidase M16 domain-containing protein | -2.60 | down |
| DW66_5351 | peptidase M16 domain-containing protein | -2.12 | down |
| DW66_5353 | peptidase M16 domain-containing protein | -3.94 | down |
| DW66_5354 | peptidase M16 domain-containing protein | -1.82 | down |
| DW66_5358 | aldehyde dehydrogenase | 1.04 | up |
| DW66_5359 | hypothetical protein | 1.53 | up |
| DW66_5361 | phosphopantetheine adenylyltransferase | -1.36 | down |
| DW66_5362 | 4Fe-4S ferredoxin | -1.28 | down |
| DW66_5364 | signal transduction protein | -3.11 | down |
| DW66_5366 | LSU m5C1962 methyltransferase RlmI | -1.54 | down |
| DW66_5368 | hypothetical protein | -5.32 | down |
| DW66_5369 | NAD(P)H dehydrogenase (quinone) | -1.34 | down |
| DW66_5370 | Phosphoserine phosphatase | -1.81 | down |
| DW66_5371 | sodium:dicarboxylate symporter | -4.94 | down |
| DW66_5373 | integral membrane protein | -1.32 | down |
| DW66_5375 | metal dependent phosphohydrolase | -2.11 | down |
| DW66_5377 | 2-aminoethylphosphonate ABC transporter permease | -5.83 | down |
| DW66_5378 | 2-aminoethylphosphonate ABC transporter ATP-binding protein | -15.38 | down |
| DW66_5379 | transcriptional regulator LysR | -1.73 | down |
| DW66_5383 | prolipoprotein diacylglyceryl transferase | -3.43 | down |
| DW66_5384 | hypothetical protein | -3.94 | down |
| DW66_5389 | integral membrane protein | 2.77 | up |
| DW66_5390 | threonine dehydratase | -2.62 | down |
| DW66_5392 | DNA-binding protein | -2.06 | down |
| DW66_5394 | SdiA-regulated domain-containing protein | -3.35 | down |
| DW66_5400 | periplasmic binding protein from ABC-type transporter | -2.65 | down |
| DW66_5401 | Purine nucleoside phosphorylase | -2.33 | down |
| DW66_5402 | 2OG-Fe(II) oxygenase | -3.44 | down |
| DW66_5403 | hypothetical protein | -1.47 | down |
| DW66_5404 | lipase | -2.15 | down |
| DW66_5405 | Thiol-disulfide isomerase and thioredoxins | -3.43 | down |
| DW66_5410 | NLPA lipoprotein | -5.64 | down |
| DW66_5411 | *sigma*-54 dependent transcriptional regulator | -5.34 | down |
| DW66_5412 | hypothetical protein | -4.64 | down |
| DW66_5414 | sulfate ABC transporter permease | -2.26 | down |
| DW66_5415 | sulfate ABC transporter permease | -3.76 | down |
| DW66_5416 | sulfate ABC transporter | -4.28 | down |
| DW66_5417 | hypothetical protein | -6.14 | down |
| DW66_5418 | acriflavin resistance protein | 1.35 | up |
| DW66_5423 | ornithine carbamoyltransferase | 1.38 | up |
| DW66_5426 | extracellular solute-binding protein | -1.57 | down |
| DW66_5429 | glutamate-putrescine ligase | -1.13 | down |
| DW66_5435 | hypothetical protein | 4.03 | up |
| DW66_5436 | type II secretion system protein E | -2.41 | down |
| DW66_5437 | Ribonucleotide reductase, alpha subunit | -1.39 | down |
| DW66_5438 | glycine dehydrogenase | 3.92 | up |
| DW66_5439 | IS111A/IS1328/IS1533, transposase | 1.23 | up |
| DW66_5561 | glycine cleavage system aminomethyltransferase T | -1.43 | down |
| DW66_5562 | hypothetical protein | -11.64 | down |
| DW66_5563 | binding-protein-dependent transport system inner membrane protein | -3.15 | down |
| DW66_5564 | extracellular solute-binding protein | -2.67 | down |
| DW66_5565 | 2-octaprenyl-3-methyl-6-methoxy-1,4-benzoquinol hydroxylase | -1.02 | down |
| DW66_5566 | hypothetical protein | -1.54 | down |
| DW66_5567 | 2-octaprenyl-6-methoxyphenyl hydroxylase | -1.28 | down |
| DW66_5568 | peptidase M24 | -1.84 | down |
| DW66_5569 | hypothetical protein | -1.45 | down |
| DW66_5571 | Z-ring-associated protein ZapA | -1.01 | down |
| DW66_5574 | hypothetical protein | -1.74 | down |
| DW66_5576 | ABC transporter ATP-binding protein/permease | -1.26 | down |
| DW66_5579 | alcohol dehydrogenase | 1.60 | up |
| DW66_5581 | CDP-6-deoxy-delta-3,4-glucoseen reductase | -2.07 | down |
| DW66_5582 | decarboxylase UbiD | -2.26 | down |
| DW66_5583 | transcription termination factor Rho | -3.02 | down |
| DW66_5584 | thioredoxin | 1.34 | up |
| DW66_5591 | nucleoside diphosphate kinase regulator | 1.09 | up |
| DW66_5600 | hypothetical protein | 2.16 | up |
| DW66_5601 | ammonium transporter | 1.74 | up |
| DW66_5602 | nitrogen regulatory protein P-II 1 | 2.44 | up |
| DW66_5604 | protein AIG2 | -1.36 | down |
| DW66_5605 | Mg chelatase, ChlI subunit | -3.01 | down |
| DW66_5606 | recombinase-related protein | -1.05 | down |
| DW66_5607 | hypothetical protein | -2.80 | down |
| DW66_5608 | ISPpu15, transposase | -1.36 | down |
| DW66_5609 | ISPpu15, transposase | -4.29 | down |
| DW66_5610 | hypothetical protein | -6.16 | down |
| DW66_5611 | hypothetical protein | -4.75 | down |
| DW66_5612 | Mg chelatase, ChlI subunit | -1.10 | down |
| DW66_5616 | sulfate transporter | -1.65 | down |
| DW66_5617 | hypothetical protein | -1.72 | down |
| DW66_5619 | hypothetical protein | -1.68 | down |
| DW66_5621 | integrase | -2.61 | down |
| DW66_5622 | hypothetical protein | -2.85 | down |
| DW66_5623 | Transcriptional regulator | -4.35 | down |
| DW66_5624 | hypothetical protein | -8.46 | down |
| DW66_5625 | hypothetical protein | -6.59 | down |
| DW66_5626 | Phage capsid and scaffold protein | -13.38 | down |
| DW66_5627 | hypothetical protein | -12.60 | down |
| DW66_5628 | hypothetical protein | -3.43 | down |
| DW66_5629 | hypothetical protein | -11.73 | down |
| DW66_5630 | hypothetical protein | -12.46 | down |
| DW66_5631 | hypothetical protein | -5.37 | down |
| DW66_5632 | Phage terminase, small subunit | -4.23 | down |
| DW66_5633 | Phage terminase, large subunit | -4.49 | down |
| DW66_5634 | Mu-like prophage FluMu protein gp29 | -2.73 | down |
| DW66_5635 | virion morphogenesis protein | -3.29 | down |
| DW66_5636 | phage virion morphogenesis protein | -4.70 | down |
| DW66_5637 | Phage protein | -4.49 | down |
| DW66_5638 | hypothetical protein | -3.87 | down |
| DW66_5639 | hypothetical protein | -10.48 | down |
| DW66_5641 | membrane protein | -3.34 | down |
| DW66_5642 | Mu-like prophage FluMu protein gp36 | -3.38 | down |
| DW66_5643 | hypothetical protein | -4.22 | down |
| DW66_5644 | tail protein | -2.13 | down |
| DW66_5645 | tail protein | 1.46 | up |
| DW66_5646 | hypothetical protein | -1.92 | down |
| DW66_5647 | hypothetical protein | -3.58 | down |
| DW66_5648 | hypothetical protein | -1.89 | down |
| DW66_5652 | baseplate protein | -1.05 | down |
| DW66_5653 | baseplate protein | -2.47 | down |
| DW66_5654 | Phage-related baseplate assembly protein | -3.87 | down |
| DW66_5655 | tail protein I | -3.35 | down |
| DW66_5656 | hypothetical protein | -2.53 | down |
| DW66_5657 | phage-related hypothetical protein | -2.10 | down |
| DW66_5658 | hypothetical protein | -3.04 | down |
| DW66_5660 | response regulator receiver protein | -2.38 | down |
| DW66_5664 | transcriptional regulator AraC | -11.25 | down |
| DW66_5665 | transcriptional regulator AraC | -2.27 | down |
| DW66_5666 | potassium efflux system protein | -1.28 | down |
| DW66_5667 | hypothetical protein | 1.89 | up |
| DW66_5669 | transcriptional regulator LysR | -2.35 | down |
| DW66_5670 | transcriptional regulator LysR | -1.18 | down |
| DW66_5671 | outer membrane porin | -2.82 | down |
| DW66_5672 | transporter | -1.37 | down |
| DW66_5674 | hypothetical protein | -2.44 | down |
| DW66_5676 | membrane protein | -4.68 | down |
| DW66_5677 | isochorismatase hydrolase | 1.22 | up |
| DW66_5679 | FAD dependent oxidoreductase | 2.96 | up |
| DW66_5680 | aldehyde dehydrogenase | 5.37 | up |
| DW66_5682 | hypothetical protein | 3.44 | up |
| DW66_5683 | transcriptional regulator LysR | -1.02 | down |
| DW66_5684 | multidrug efflux protein NorA | -1.54 | down |
| DW66_5685 | diguanylate cyclase/phosphodiesterase | -2.77 | down |
| DW66_5686 | ATP-dependent DNA helicase Rep | -2.86 | down |
| DW66_5689 | cytochrome c5 | 2.87 | up |
| DW66_5690 | transcriptional regulator XRE | -1.21 | down |
| DW66_5691 | alanine racemase | 1.63 | up |
| DW66_5692 | D-amino acid dehydrogenase small subunit | 2.06 | up |
| DW66_5695 | FAD dependent oxidoreductase | -1.56 | down |
| DW66_5699 | transporter protein | -3.01 | down |
| DW66_5700 | aldehyde dehydrogenase | -2.06 | down |
| DW66_5701 | hypothetical protein | 1.07 | up |
| DW66_5702 | 50S ribosomal protein L33 | -1.51 | down |
| DW66_5703 | 50S ribosomal protein L28 | -2.16 | down |
| DW66_5706 | bifunctional phosphopantothenoylcysteine decarboxylase/phosphopantothenate synthase | -1.46 | down |
| DW66_5707 | deoxyuridine 5'-triphosphate nucleotidohydrolase | -1.46 | down |
| DW66_5711 | exodeoxyribonuclease III | 1.12 | up |
| DW66_5712 | hypothetical protein | -1.06 | down |
| DW66_5714 | Protein YicC | -1.36 | down |
| DW66_5715 | guanylate kinase | -1.14 | down |
| DW66_5716 | amino acid permease-associated protein | -1.05 | down |
| DW66_5719 | transcriptional regulator LysR | -1.04 | down |
| DW66_5720 | DNA-directed RNA polymerase subunit omega | -1.07 | down |
| DW66_5721 | (p)ppGpp synthetase I SpoT/RelA | -1.50 | down |
| DW66_5723 | lipoprotein | -2.43 | down |
| DW66_5725 | TonB-system energizer ExbB type-1 | -3.93 | down |
| DW66_5726 | biopolymer transport protein ExbD | -4.04 | down |
| DW66_5727 | TonB protein | -3.62 | down |
| DW66_5729 | ATP-dependent DNA helicase RecG | -1.89 | down |
| DW66_5731 | DNA and RNA helicases | -1.59 | down |
| DW66_5733 | FAD-dependent pyridine nucleotide-disulfide oxidoreductase | -1.45 | down |
| DW66_5735 | chorismate lyase | -2.64 | down |
| DW66_5736 | 4-hydroxybenzoate octaprenyltransferase | -2.18 | down |
| DW66_5737 | hypothetical protein | 5.41 | up |
| DW66_5738 | transcriptional regulator | 1.26 | up |
| DW66_5739 | PAS/PAC sensor signal transduction histidine kinase | -1.62 | down |
| DW66_5740 | metal ion transporter | -1.15 | down |
| DW66_5741 | peptidase | 2.85 | up |
| DW66_5742 | response regulator receiver protein | -1.96 | down |
| DW66_5743 | phosphate uptake regulator PhoU | -1.64 | down |
| DW66_5744 | phosphate ABC transporter ATP-binding protein | -1.15 | down |
| DW66_5745 | phosphate ABC transporter permease | -1.23 | down |
| DW66_5746 | binding-protein-dependent transport system inner membrane protein | -1.83 | down |
| DW66_5747 | phosphate binding protein | -1.25 | down |
| DW66_5748 | transporter protein | -2.24 | down |
| DW66_5752 | transglycosylase-associated protein | 1.69 | up |
| DW66_5753 | phosphoribosylaminoimidazole carboxylase ATPase subunit | -2.26 | down |
| DW66_5754 | phosphoribosylaminoimidazole carboxylase catalytic subunit | -2.08 | down |
| DW66_5755 | Ribosomal protein S8 | -2.00 | down |
| DW66_5757 | hypothetical protein | -1.43 | down |
| DW66_5758 | aspartate ammonia-lyase | 3.79 | up |
| DW66_5760 | histone deacetylase protein | -1.05 | down |
| DW66_5763 | FMN-binding negative transcriptional regulator | 2.63 | up |
| DW66_5764 | acetyltransferase | 2.51 | up |
| DW66_5765 | acetyltransferase | 1.71 | up |
| DW66_5766 | pyruvate carboxylase subunit B | 1.58 | up |
| DW66_5767 | pyruvate carboxylase subunit A | 2.96 | up |
| DW66_5773 | DNA-dependent helicase II | -1.60 | down |
| DW66_5775 | hypothetical protein | 1.87 | up |
| DW66_5776 | sodium/hydrogen exchanger | -2.42 | down |
| DW66_5777 | hypothetical protein | -1.13 | down |
| DW66_5779 | pyridoxamine kinase | -1.09 | down |
| DW66_5781 | cobalamin synthesis protein P47K | -2.74 | down |
| DW66_5782 | hypothetical protein | -5.31 | down |
| DW66_5783 | cobalamin synthesis protein P47K | -5.07 | down |
| DW66_5784 | GTP cyclohydrolase | -6.79 | down |
| DW66_5787 | cyclopropane-fatty-acyl-phospholipid synthase | 1.31 | up |
| DW66_5788 | dihydrolipoamide dehydrogenase | 1.10 | up |
| DW66_5790 | copper resistance protein B | 1.74 | up |
| DW66_5791 | Multicopper oxidase | 3.03 | up |
| DW66_5793 | hypothetical protein | 3.84 | up |
| DW66_5795 | ATPase | -1.18 | down |
| DW66_5796 | cytochrome C | -3.15 | down |
| DW66_5797 | cytochrome C biogenesis protein | -3.66 | down |
| DW66_5799 | cation transporter | -3.40 | down |
| DW66_5801 | isoprenylcysteine carboxyl methyltransferase | 2.11 | up |
| DW66_5802 | hypothetical protein | 2.57 | up |
| DW66_5803 | hypothetical protein | 3.55 | up |
| DW66_5804 | hypothetical protein | 3.82 | up |
| DW66_5805 | heavy metal transport/detoxification protein | 5.72 | up |
| DW66_5807 | Mobile element protein | -2.56 | down |
| DW66_5809 | sugar transferase | -2.65 | down |
| DW66_5810 | ribonuclease III | -6.03 | down |
| DW66_5811 | transcriptional regulator LysR | -2.46 | down |
| DW66_5812 | phosphate-selective porin O and P | -1.29 | down |
| DW66_5814 | hypothetical protein | -4.35 | down |
| DW66_5816 | cadmium-transporting P-type ATPase CadA1 | -1.01 | down |
| DW66_5817 | hypothetical protein | 3.61 | up |
| DW66_5818 | cobalt/zinc/cadmium efflux transporter permease | -2.92 | down |
| DW66_5819 | cobalt/zinc/cadmium efflux transporter membrane fusion protein | -3.11 | down |
| DW66_5820 | cobalt/zinc/cadmium efflux transporter outer membrane protein | -3.69 | down |
| DW66_5821 | porin | -3.28 | down |
| DW66_5822 | IS4, transposase | -1.36 | down |
| DW66_5823 | porin | -2.35 | down |
| DW66_5824 | DNA-binding heavy metal response regulator | -1.20 | down |
| DW66_5826 | ATPase AAA | -3.00 | down |
| DW66_5827 | ISPsy14, transposase | -2.79 | down |
| DW66_5828 | peptidase | -2.06 | down |
| DW66_5829 | hypothetical protein | -1.29 | down |
| DW66_5830 | chromate transport protein | -12.61 | down |
| DW66_5832 | transposase | -1.38 | down |
| DW66_5836 | transcriptional regulator LysR | -1.05 | down |
| DW66_5837 | endoribonuclease L-PSP | 2.34 | up |
| DW66_5838 | transposase | 3.14 | up |
| DW66_5840 | mercuric reductase | 1.18 | up |
| DW66_5841 | mercury transporter MerC | 1.11 | up |
| DW66_5842 | mercury transporter | 1.03 | up |
| DW66_5843 | mercuric ion transport protein | -1.40 | down |
| DW66_5845 | transposase | -3.93 | down |
| DW66_5846 | Fe-S cluster assembly protein SufE | 1.77 | up |
| DW66_5847 | Protein YidD | -2.44 | down |
| DW66_5848 | MerD2 protein | -1.20 | down |
| DW66_5850 | mercuric ion reductase | 2.72 | up |
| DW66_5851 | mercury transporter | 3.40 | up |
| DW66_5852 | mercuric ion transport protein | 2.66 | up |
| DW66_5854 | addiction module antitoxin RelB | -2.76 | down |
| DW66_5855 | ISPsy5, transposase | -4.51 | down |
| DW66_5857 | transposase | -12.18 | down |
| DW66_5858 | hypothetical protein | -1.56 | down |
| DW66_5860 | MerT | -2.64 | down |
| DW66_5864 | Signal recognition particle receptor protein FtsY | -4.03 | down |
| DW66_5865 | transcriptional regulator MerR | -4.07 | down |
| DW66_5866 | Transposon Tn21 resolvase | -2.54 | down |
| DW66_5867 | transposase for Tn21 | -2.04 | down |
| DW66_5868 | TnpT protein | 2.04 | up |
| DW66_5869 | Tn4652, cointegrate resolution protein S | 2.40 | up |
| DW66_5870 | hypothetical protein | -4.27 | down |
| DW66_5871 | hypothetical protein | -1.43 | down |
| DW66_5872 | hypothetical protein | -3.51 | down |
| DW66_5873 | hypothetical protein | -9.74 | down |
| DW66_5874 | ISPsy14, transposase | -2.79 | down |
| DW66_5875 | ATPase AAA | -2.98 | down |
| DW66_5876 | hypothetical protein | -10.64 | down |
| DW66_5877 | hypothetical protein | 1.74 | up |
| DW66_5879 | chromosome segregation protein SMC | -1.14 | down |
| DW66_5882 | hypothetical protein | 2.29 | up |
| DW66_5883 | DNA-binding protein | 1.37 | up |
| DW66_5884 | Ser/Thr protein phosphatase | -1.85 | down |
| DW66_5885 | integrase | -2.24 | down |
| DW66_5889 | glutathione S-transferase | 1.03 | up |
| DW66_5890 | ThiJ/PfpI domain protein | 2.07 | up |
| DW66_5893 | ATPase AAA | -3.00 | down |
| DW66_5894 | ISPsy14, transposase | -2.79 | down |
