# Supplementary material for: The Regulation of para-Nitrophenol Degradation in Pseudomonas putida DLL-E4
Source: PLoS One. 2016 May 18;11(5):e0155485. doi: 10.1371/journal.pone.0155485 (PMC4871426; doi:10.1371/journal.pone.0155485)
Supplement: S5 Table — (DOCX) [file pone.0155485.s006.docx]

**Table S5.** **The proportion of two ncRNAs in the transcribed intergenic sequences.**

| **Gene ID** | **Gene name** | **E4-G^a^** | **E4-GP^b^** | **R-G^c^** | **R-GP^d^** |
| --- | --- | --- | --- | --- | --- |
| **ncRNA-214** | *ins1* | 12.26% | 14.60% | 7.47% | 15.56% |
| **ncRNA-223** | *ins2* | 36.31% | 61.70% | 31.86% | 54.72% |

^a^E4-G represents strain DLL-E4 grown on 0.25% glucose.

^b^E4-GP represents strain DLL-E4 grown on 0.25% glucose plus 0.5 mM PNP.

^c^R-G represents strain DLL-△*pnpR* grown on 0.25% glucose.

^d^R-GP represents strain DLL-△*pnpR* grown on 0.25% glucose plus 0.5 mM PNP.
