# Supplementary material for: The Regulation of para-Nitrophenol Degradation in Pseudomonas putida DLL-E4
Source: PLoS One. 2016 May 18;11(5):e0155485. doi: 10.1371/journal.pone.0155485 (PMC4871426; doi:10.1371/journal.pone.0155485)
Supplement: S6 Table — The fold changes are reported in log2-based format. (DOCX) [file pone.0155485.s007.docx]

**Table S6. Differentially expressed genes related to ribosomal proteins synthesis, rRNA synthesis, and RNA polymerase sigma factors in *P. putida* DLL-E4 and DLL-△*pnpR*. The fold changes are reported in log_2_-based format.**

| **Gene ID** | **Function** | **Fold change (log_2_)** | |
| --- | --- | --- | --- |
|  |  | **E4-GP vs E4-G^a^** | **R-GP vs R-G^b^** |
| **DW66_0020** | 50S ribosomal protein L34 | -4.16 | -3.35 |
| **DW66_0338** | Ribosomal protein S6 glutaminyl transferase | -1.72 | -1.33 |
| **DW66_0398** | 30S ribosomal protein S21 | -1.52 | -1.18 |
| **DW66_0452** | 50S ribosomal protein L11 | -3.99 | -3.12 |
| **DW66_0453** | 50S ribosomal protein L1 | -3.78 | -2.83 |
| **DW66_0454** | 50S ribosomal protein L10 | -4.68 | -3.54 |
| **DW66_0455** | 50S ribosomal protein L7/L12 | -2.18 | -1.63 |
| **DW66_0458** | 30S ribosomal protein S12 | -2.29 | -1.44 |
| **DW66_0463** | 30S ribosomal protein S10 | -3.38 | -2.52 |
| **DW66_0464** | ribosomal protein L3 | -4.12 | -3.15 |
| **DW66_0465** | 50S ribosomal protein L4 | -3.79 | -2.51 |
| **DW66_0466** | 50S ribosomal protein L23 | -3.48 | -1.94 |
| **DW66_0467** | 50S ribosomal protein L2 | -3.44 | -2.00 |
| **DW66_0468** | 30S ribosomal protein S19 | -3.15 | -1.62 |
| **DW66_0469** | 50S ribosomal protein L22 | -3.49 | -1.85 |
| **DW66_0470** | ribosomal protein S3 | -3.40 | -1.39 |
| **DW66_0471** | 50S ribosomal protein L16 | -3.14 | -1.61 |
| **DW66_0472** | 50S ribosomal protein L29 | -2.63 | -1.60 |
| **DW66_0478** | 30S ribosomal protein S8 | -3.06 | -2.92 |
| **DW66_0479** | 50S ribosomal protein L6 | -3.07 | -2.93 |
| **DW66_0480** | 50S ribosomal protein L18 | -3.02 | -2.61 |
| **DW66_0481** | 30S ribosomal protein S5 | -3.30 | -2.67 |
| **DW66_0482** | 50S ribosomal protein L30 | -3.54 | -2.39 |
| **DW66_0483** | 50S ribosomal protein L15 | -3.40 | -2.32 |
| **DW66_0486** | 30S ribosomal protein S13 | -1.77 | -1.31 |
| **DW66_0487** | 30S ribosomal protein S11 | -2.13 | -1.24 |
| **DW66_0618** | 30S ribosomal protein S20 | -2.79 | -2.34 |
| **DW66_0692** | ribosomal protein L21 | -2.96 | -2.30 |
| **DW66_0693** | 50S ribosomal protein L27 | -2.72 | -1.46 |
| **DW66_0741** | ribose-phosphate pyrophosphokinase | -2.36 | -2.95 |
| **DW66_1062** | 30S ribosomal protein S16 | -2.59 | -2.63 |
| **DW66_1063** | 16S rRNA-processing protein RimM | -3.67 | -3.37 |
| **DW66_1065** | 50S ribosomal protein L19 | -2.43 | -1.50 |
| **DW66_1301** | ribosomal large subunit pseudouridine synthase A | -2.97 | -2.63 |
| **DW66_1702** | 30S ribosomal protein S1 | -1.89 | -1.26 |
| **DW66_1831** | 50S ribosomal protein L32 | -3.10 | -2.93 |
| **DW66_2287** | ribosomal protein L35 | -2.39 | -2.14 |
| **DW66_2288** | 50S ribosomal protein L20 | -2.16 | -2.01 |
| **DW66_3689** | Ribosomal protein S3AE | -1.99 | -2.34 |
| **DW66_4767** | 30S ribosomal protein S9 | -2.62 | -2.36 |
| **DW66_4768** | 50S ribosomal protein L13 | -3.02 | -2.68 |
| **DW66_5050** | 50S ribosomal protein L11 methyltransferase | -1.60 | -1.96 |
| **DW66_5110** | 50S ribosomal protein L9 | -2.20 | -1.17 |
| **DW66_5220** | 16S ribosomal RNA methyltransferase RsmE | -1.29 | -1.07 |
| **DW66_5253** | 16S ribosomal RNA methyltransferase RsmE | -3.54 | -2.37 |
| **DW66_5703** | 50S ribosomal protein L28 | -2.16 | -1.67 |
| **DW66_5755** | Ribosomal protein S8 | -2.00 | -1.88 |
| **DW66_5288** | RNA methyltransferase | -1.56 | -2.52 |
| **DW66_0868** | RNA methyltransferase | -1.30 | -1.11 |
| **DW66_1969** | RNA 2'-O-ribose methyltransferase | -3.90 | -4.41 |
| **DW66_0702** | ribosomal-protein-alanine acetyltransferase | -1.62 | -1.03 |
| **DW66_4756** | ribosomal RNA small subunit methyltransferase I | -1.05 | -1.17 |
| **DW66_1661**  **DW66_3791**  **DW66_3634**  **DW66_4825**  **DW66_3343**  **DW66_0996**  **DW66_0895**  **DW66_0141**  **DW66_4613**  **DW66_4051**  **DW66_0362** | ribosomal pseudouridine synthase A, large subunit  RNA polymerase sigma-71 factor  RNA polymerase sigma-70 factor  RNA polymerase sigma-30 factor  RNA polymerase sigma-29 factor  RNA polymerase sigma-26 factor  RNA polymerase sigma-25 factor  RNA polymerase sigma-24 factor  RNA polymerase sigma factor AlgU  RNA polymerase sigma factor  RNA polymerase sigma factor | -2.91  -4.87  -4.87  -7.06  -3.16  -5.67  -3.97  -2.55  -1.39  -3.30  -3.00 | -2.76  -5.11  -5.11  -5.53  -3.55  -4.74  -5.38  -1.52  -2.56  -3.53  -2.63 |

^a^Fold changes in expression levels in strain DLL-E4 grown on 0.25% glucose plus 0.5 mM PNP compared to 0.25% glucose.

^b^Fold changes in expression levels in strain DLL-△*pnpR* grown on 0.25% glucose plus 0.5 mM PNP compared to 0.25% glucose.

Values below −1 represent downregulation between the tested conditions, values above 1 represent upregulation between the tested conditions, and values between −1 and 1 indicate no differential expression between the tested conditions.
