# Supplementary material for: The Regulation of para-Nitrophenol Degradation in Pseudomonas putida DLL-E4
Source: PLoS One. 2016 May 18;11(5):e0155485. doi: 10.1371/journal.pone.0155485 (PMC4871426; doi:10.1371/journal.pone.0155485)
Supplement: S7 Table — The fold changes are reported in log2-based format. (DOCX) [file pone.0155485.s008.docx]

**Table S7. Differentially expressed genes related to glucose transport and oxidization in *P. putida* DLL-E4 and DLL-△*pnpR*. The fold changes are reported in log_2_-based format.**

| **Gene ID** | **Gene name** | **Function** | **Fold change (log_2_)** | |
| --- | --- | --- | --- | --- |
|  |  |  | **E4-GP vs E4-G^a^** | **R-GP vs R-G^b^** |
| **DW66_1007** | *oprB* | outer membrane porin, uptake glucose from outer membrane to periplasmic | 0.35 | 4.05 |
| **DW66_1003** | *gtsA* | sugar ABC transporter, periplasmic sugar-binding protein | -0.76 | 2.11 |
| **DW66_1004** | *gtsB* | sugar ABC transporter, permease protein | -2.87 | 1.29 |
| **DW66_1005** | *gtsC* | sugar ABC transporter, permease protein | -2.76 | 2.33 |
| **DW66_1006** | *gtsD* | sugar ABC transporter, ATP-binding subunit | -1.76 | 3.81 |
| **DW66_1009** | *hexR* | transcriptional regulator glucose metabolism | -1.48 | -1.62 |
| **DW66_1000** | *gltR* | glucose transporter activator | -1.60 | -0.11 |
| **DW66_3152** | *gntP* | gluconate transporter, transport gluconate from periplamic to cytoplasm | 0.43 | 1.75 |
| **DW66_3113** | *kguT* | 2-keto-gluconate transporter, transport 2-keto-gluconate from periplasmic to cytoplasm | -0.99 | 3.36 |
| **DW66_4595** | *gcd* | glucose dehydrogenase, oxidize glucose to gluconate | 1.03 | 1.84 |

^a^Fold changes in expression levels in strain DLL-E4 grown on 0.25% glucose plus 0.5 mM PNP compared to 0.25% glucose.

^b^Fold changes in expression levels in strain DLL-△*pnpR* grown on 0.25% glucose plus 0.5 mM PNP compared to 0.25% glucose.

Values below −1 represent downregulation between the tested conditions, values above 1 represent upregulation between the tested conditions, and values between −1 and 1 indicate no differential expression between the tested conditions.
